# Insights into the genome structure and copy-number variation of *Eimeria tenella*

**DOI:** 10.1186/1471-2164-13-389

**Published:** 2012-08-13

**Authors:** Lik-Sin Lim, Yea-Ling Tay, Halimah Alias, Kiew-Lian Wan, Paul H Dear

**Affiliations:** 1 School of Biosciences and Biotechnology, Faculty of Science and Technology, Universiti Kebangsaan Malaysia, 43600, UKM Bangi, Selangor DE, Malaysia; 2 Malaysia Genome Institute, Jalan Bangi, 43000, Kajang, Selangor DE, Malaysia; 3 MRC Laboratory of Molecular Biology, Hills Road, Cambridge, CB2 0QH, United Kingdom

## Abstract

**Background:**

*Eimeria* is a genus of parasites in the same phylum (Apicomplexa) as human parasites such as *Toxoplasma*, *Cryptosporidium* and the malaria parasite *Plasmodium.* As an apicomplexan whose life-cycle involves a single host, *Eimeria* is a convenient model for understanding this group of organisms. Although the genomes of the Apicomplexa are diverse, that of *Eimeria* is unique in being composed of large alternating blocks of sequence with very different characteristics - an arrangement seen in no other organism. This arrangement has impeded efforts to fully sequence the genome of *Eimeria*, which remains the last of the major apicomplexans to be fully analyzed. In order to increase the value of the genome sequence data and aid in the effort to gain a better understanding of the *Eimeria tenella* genome, we constructed a whole genome map for the parasite.

**Results:**

A total of 1245 contigs representing 70.0% of the whole genome assembly sequences (Wellcome Trust Sanger Institute) were selected and subjected to marker selection. Subsequently, 2482 HAPPY markers were developed and typed. Of these, 795 were considered as usable markers, and utilized in the construction of a HAPPY map. Markers developed from chromosomally-assigned genes were then integrated into the HAPPY map and this aided the assignment of a number of linkage groups to their respective chromosomes. BAC-end sequences and contigs from whole genome sequencing were also integrated to improve and validate the HAPPY map. This resulted in an integrated HAPPY map consisting of 60 linkage groups that covers approximately half of the estimated 60 Mb genome. Further analysis suggests that the segmental organization first seen in Chromosome 1 is present throughout the genome, with repeat-poor (P) regions alternating with repeat-rich (R) regions. Evidence of copy-number variation between strains was also uncovered.

**Conclusions:**

This paper describes the application of a whole genome mapping method to improve the assembly of the genome of *E. tenella* from shotgun data, and to help reveal its overall structure. A preliminary assessment of copy-number variation (extra or missing copies of genomic segments) between strains of *E. tenella* was also carried out. The emerging picture is of a very unusual genome architecture displaying inter-strain copy-number variation. We suggest that these features may be related to the known ability of this parasite to rapidly develop drug resistance.

## Background

The phylum Apicomplexa contains a diverse range of parasites including *Plasmodium*, *Cryptosporidium, Babesia, Toxoplasma* and others that cause disease in both humans and animals. The genomes of several apicomplexans have been extensively studied [1-5] in an attempt to understand these organisms and to gain insights into potential new methods of control and treatment. Although a few common features have emerged, the apicomplexan genomes studied to date have presented a remarkable diversity of genomic organization and gene content, with each genome having its own unique, and often unusual, characteristics. The genus *Eimeria*, belonging to the family Eimeriidae within the order Eucoccidiorida (commonly known as the coccidians) represents the last major group of apicomplexans to be analyzed in detail. Because it is homoxenous, *Eimeria* can be propagated to high numbers in a single life-cycle with relative ease, making it a potential model for aspects of apicomplexan biology that are difficult to study in other genera.

*Eimeria* is also significant in its own right, as the causative agent of the intestinal disease coccidiosis in poultry [6]. *Eimeria* species are both site- and host-specific, and are transmitted through the ingestion of sporulated oocysts - resistant, hardy, thick-walled spores that contain infective sporozoites [7]. Seven species infect chickens and *Eimeria tenella* is among the most pathogenic [8] causing weight loss, reduced feed efficiency, reduced egg production and death. The total loss including the costs of control and prevention worldwide is estimated at around USD2.4 billion per annum, making this one of the most economically important diseases of domestic livestock [9].

The desire for a better understanding of the parasite and its interaction with the chicken, and a need for better disease control, had driven the *E. tenella* genome sequencing project [10]. A draft genome assembly (released by the Wellcome Trust Sanger Institute in May 2007) of ~8.3-fold sequence coverage contains 4707 contigs, ranging from thousands to half a million bases in length. The total size of these contigs is 47 Mb corresponding to ~78% of the estimated 60 Mb genome. A further assembly, incorporating second-generation sequencing data, was produced in 2010, but much of the genome remains unrepresented by large contigs.

The completion of eukaryotic genome sequencing projects depends heavily on having good genome maps to position contigs, give information on large-scale genome structure, and reveal errors in sequence assembly [11-13]. Such a map has hitherto been lacking in the case of *E. tenella*. The available genetic linkage map*,* which relies on polymorphic loci, is low in resolution and does not reflect the genome physically [14]. Furthermore, large insert clones are difficult to produce for *Eimeria* species as they are often unstable due to repetitive sequences [15], eliminating an effective method for physical mapping.

HAPPY mapping [16] is an *in vitro* physical mapping technique which analyses markers’ co-segregation amongst a pool of sub-genomic samples (each containing an approximately haploid amount of randomly sheared DNA). Markers that are close together tend to co-segregate strongly amongst the aliquots. Each marker will be typed by PCR to detect its presence in each aliquot and the probability of linkage between two markers is given as a logarithm of odds (LOD) score. Based on the LOD scores, a genome map can be constructed. This technique was successful in assisting the assembly of *E. tenella* chromosomes 1 and 2 with individual maps constructed respectively by Ling et al. [17] and Paul H. Dear (unpublished data).

Mapping also provides an opportunity to examine genomic variation. Comparisons between different strains of *E. tenella* have found only limited sequence variation [18,19]. Recent studies in several species (including human) have shown that structural variation (duplication or rearrangement) contributes more diversity than sequence variation [20], but there have been no efforts to look into structural variations in *Eimeria* or other Apicomplexa. HAPPY mapping can be extended to allow molecular copy-number counting (MCC), determining copy-numbers by counting the molecules present [21]. MCC has been used to screen variations in copy-number due to its speed, flexibility and sensitivity [22-24].

As HAPPY maps for *E. tenella* chromosomes 1 and 2 have previously been constructed, we demonstrate in this study the construction of an integrated map for the remainder of the genome in order to give an overview of genome organization and to aid sequence assembly. We managed to improve and validate the map by integrating BAC-end and contig sequences. We find that the striking segmentation of the genome into feature-poor (P) and feature-rich (R) regions (previously noted on Chromosome 1) is present throughout the genome, and that the P- and R-segments correspond to unique and non-unique regions, respectively. Investigation of copy-number variation using MCC has highlighted further structural perspectives on the *E. tenella* genome.

## Results

### Whole genome HAPPY map of *Eimeria tenella*

Markers were generated from the draft genome assembly (released in May 2007) obtained from the Wellcome Trust Sanger Institute (WTSI). Contigs similar to Chromosome 1 [17] and 2 (unpublished data) were filtered prior to marker generation. A total of 1245 of the largest contigs, representing 70% of the assembled sequence, were selected. In total, 2482 markers were developed and typed. Of these, 576 (23.2%) markers failed to show amplification and therefore were discarded. A total of 914 (36.8%) multi-copy markers (recognized by an excess number of aliquots scoring positive for the marker) were also set aside while 231 (9.3%) low-copy markers were further analyzed to screen for significant linkage. Overall, a total of 761 (30.7%) good markers were obtained (details in Additional file 1). This low proportion of usable markers was due largely to the presence of sequences which, though unique in the assembly, were present in multiple copies in the genome. There are 301 contigs that carry only non-multicopy markers (unique contigs) while 279 contigs contain only multi-copy markers (multi-copy contigs). In these latter cases, up to nine unsuccessful attempts were made to identify unique sequences elsewhere in the contigs. The multi-copy contigs are up to 196 kb in length.

Together with 34 low-copy markers which showed significant linkages, a total of 795 markers were used for the construction of the HAPPY map. Of these, 664 fell into 67 linkage groups of three or more markers, while 18 formed pairs of linked markers, at LOD ≥ 6 (odds of better than one million to one in favor of linkage), leaving 113 markers as singletons (details in Additional file 2). The relative physical size of the map was calculated to be 27.3 Mb, or almost half of the estimated 60 Mb genome. The HAPPY map linked 454 contigs with a total size of 18,224,408 bp, meaning that approximately 9 Mb of gaps between contigs were bridged by HAPPY linkages alone.

### Data integration

To produce a more comprehensive map, we integrated BAC-end sequences. The BAC clones of *E. tenella* have been end-sequenced in the WGS project, but were not used in the 2007 sequence assembly. We have also noticed that linkages by BAC clones alone can be unreliable, since many BAC-end sequences do not map uniquely to the genome, and some BACs show possible size discrepancies when both ends map to a single contig. However, the combination of BAC data with HAPPY map data is more robust.

Analysis of 9567 BAC-end sequences found a total of 2514 clones that aligned to different contigs of the assembly, including 277 linkages supported by at least two BACs (details in Additional file 3). Five new linkages (that is, linkages which had not already been made by HAPPY data alone) were identified by these clones, between the HAPPY linkage groups and large contigs that do not have any good markers. Most of the HAPPY linkages were supported by at least one BAC clone. Furthermore, 14 groups of multi-copy contigs were linked by BAC clones. The number of BAC clones that fall within these multi-copy contig groups often exceeds the number of clones within the HAPPY linkage group. The total size of all the contigs within the group ranged from about 100 kb to 400 kb. Two groups were found to have one of their end-markers originating from a contig that contains a good marker in one of the HAPPY linkage groups. The good markers are also situated at the ends of HAPPY linkage groups. Small contigs (unmapped in this study) are believed to lie in the gaps between contigs in the map, based on what was observed in the BAC-clone integration.

**Figure 1:**
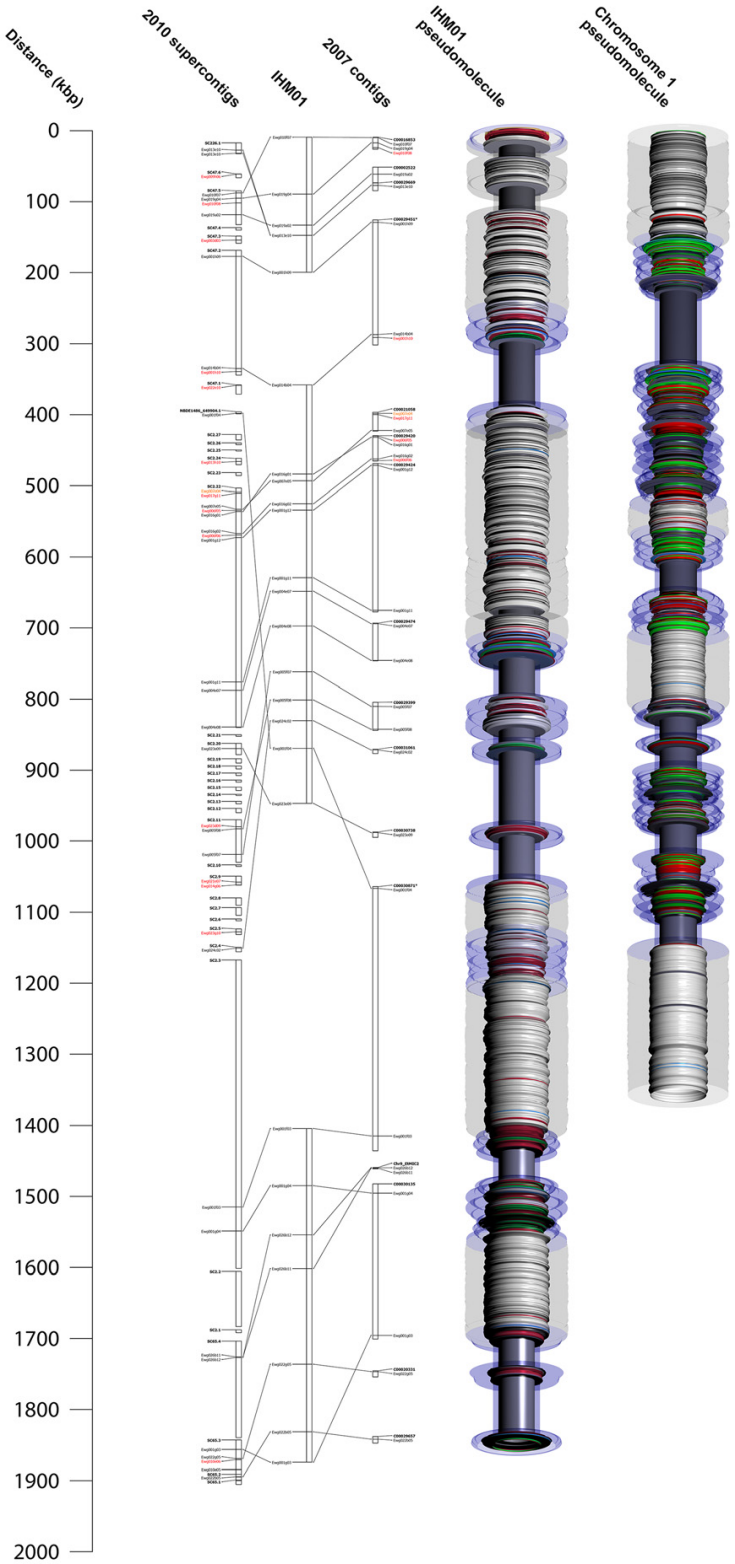
**Mapping and chromosomal segmentation in the*Eimeria tenella*genome.** One segment of the integrated map (IHM01) is shown, aligned with the supercontigs from the 2010 assembly and the contigs from the 2007 assembly. Labels “Cxxxxxx” are contig numbers in the 2007 assembly; labels “SCxx.y” indicate the supercontig (xx) and contig (y) of the 2010 assembly; HAPPY markers are named as “Ewgxxxxx”. To the right are shown the pseudomolecules for this map segment (built from the 2007 contigs) and for the previously-sequenced Chromosome 1 [17]. The diameter of the inner “spindle” indicates A/T content (5 kb sliding window; wider portions are higher in A/T), and colored bands indicate blocks of repeats of CAG (red), the telomere-like repeat unit AGGTTT (green) or other simple-sequence repeats (blue); sequence gaps are grey. The outer transparent “shell” indicates the information content (3^rd^ order Markov entropy) of the sequence (5 kb sliding window) – wider regions correspond to a higher information content, narrower regions to more repeat-rich sequence; the blue-tinted regions are R-segments, whilst the untinted regions are P-segments.

Because the HAPPY map is limited to a range of about 100 kb (due to the selected fragment size of DNA), marker-pairs from the ends of contigs longer than this are not linked by the HAPPY data, but are of course joined by the contig itself. Contig information was thus integrated to help further improve the map. Although the 2010 assembly contains larger contigs, we find conflicts between the assembly with HAPPY map (alignment between the integrated map and both of the assemblies is shown in Figure 1 and Additional file 4) and BAC linkage data. Therefore, we utilize contigs from the 2007 assembly for this purpose. Twenty pairs of markers are linked in this way. After integration, an additional nine linkages between linkage groups and 10 linkages to singletons were added to the map (details in Additional file 2).

### Simultaneous validation of contig quality and HAPPY map reliability

To monitor the quality and reliability of the HAPPY map, markers were designed at 20 kb intervals along the two largest contigs of the draft assembly. All the good markers from these two contigs were used in the subsequent map-making process and were successfully arranged into two linkage groups that correspond to their respective contigs (details in Additional file 5), and the HAPPY map arrangement of these markers corresponds well with their locations in the contigs. Similarly encouraging results come from HAPPY markers designed at opposite ends of contigs smaller than 100 kb (the maximum range of the HAPPY map): in all cases, these pairs of markers are linked, and the distance between them as estimated from the HAPPY data corresponds well with the contig length.

The BAC-end sequences also suggested that HAPPY mapping has correctly ordered the contigs, as most of the HAPPY linkages are supported by at least one BAC clone. All these results strongly suggest that both the HAPPY data and the 2007 assembly are of good quality.

### Chromosomal assignments of HAPPY linkage groups

Fifteen chromosomally-assigned genes representing ten chromosomes were used to help assign linkage groups to chromosomes (Table 1). Eleven of these genes provided good markers and, of these, ten showed linkage to other markers and were incorporated into the map. Together with the position information of the genes in the current assembly, we have successfully assigned eight linkage groups to eight different chromosomes. Among them, the largest linkage group was found to belong to Chromosome 9. None of the chromosomes is fully covered by linkage groups, but in most cases a small number of linkage groups represent each chromosome (details in Tables 1 and 2).

**Table 1:** Details of chromosomally-assigned genes

**Chr**	**Gene**	**Reference**	**Gene size (bp)**	**GenBank accession no.**	**Contig aligned**	**Gene HAPPY marker**
3	EtMIC3	[30]	3680	FJ374765	contig_00031447	NS
4	SAG 19	*	816	AJ586544	contig_00017931	S
					contig_00031465	
4	SAG 20	*	816	AJ586549	contig_00031465	S
5	EtMIC4	[30]	7766	AJ306453	contig_00029206	S
6 or 7	SAG 15	*	792	AJ586550	contig_00031359	S
6 or 7	SAG 18	*	807	AJ586548	contig_00031359	S
9	EtMIC2	[30]	1957	Z71755	contig_00030135	S
9	EtMIC5	[30]	3334	AJ245536	contig_00010048	NS
9 or 10	SAG 5	*	774	AJ586532	contig_00030134	S
9 or 10	SAG 7	*	762	AJ586533	contig_00030134	S
10	5 S rRNA	[31]	728	M86547	contig_00001191	NS
					contig_00012636	
11	SAG 1	*	762	AJ586531	contig_00031646	S
11	SAG 2	*	813	AJ586540	contig_00031646	S
12	18 S-5.8 S-28S_rDNA	[31]	1286	AY779514	contig_00004986	NS
13	EtMIC1	[30]	5990	AF032905	contig_00029639	S

*Tabares E and Tomley F, unpublished.

*S* - Successfully mapped.

*NS* - Not successfully mapped.

**Table 2:** Location of chromosomally-assigned genes or markers

**Chr**	Chromosome size* (Mb)	**Linkage group**	**Linkage group size (Mb)**
3	~2	IHM13	0.68
4	~2	IHM07	1.00
5	~2.5	IHM04	1.40
6 or 7	~3	IHM14	0.62
9	~4	IHM01	1.70
9 or 10	~4	IHM09	0.90
10	~4	Not identified	-
11	~4.5	IHM20	0.56
12	~5	Not identified	-
13	~6	IHM49	0.23

*Based on PFGE of the *Eimeria tenella* genome [32].

### The integrated HAPPY map

As a result of incorporating BAC-end and contig sequences, an integrated HAPPY map was constructed [Figure 1; Additional file 4]. The map consists of 59 linkage groups that range from 100 kb to 1.7 Mb in size, covering ~31.0 Mb. Eight of these groups are chromosomally assigned.

### Segmentation of the genome into P- and R-regions

**Figure 2:**
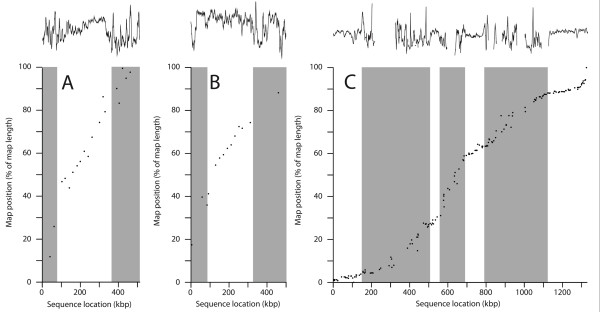
**Comparison between assembled sequence and map data.** Each graph shows the positions of mapped markers on the map, plotted against their positions in the assembled sequence, for the two largest sequence contigs of the 2007 assembly (**A**, **B**) and for the previously-reported Chromosome 1 sequence (**C**; [17]). Grey bars indicate R-regions. Above each graph is plotted the AT content (5 kb sliding window) of the sequence.

We also examined whether the segmentation of the genome into feature-rich (“R”) and feature-poor (“P”) segments, as noted on Chromosome 1 [17], could be seen on the whole genome map. An algorithm (see *Methods*) was used to partition larger contigs into P- and R-regions, and to identify smaller contigs as either P or R, based on their content of simple-sequence repeats. The overall pattern of large, alternating P- and R-segments seems to be preserved throughout the genome [Figure 1; Figure 2, with about three or four R-segments per Mb, interspersed with P-segments of roughly similar size. Deviations from this pattern are probably due to the difficulty in accurately assigning small contigs as P or R, and in defining P/R boundaries which fall close to the ends of contigs. Most of the linkage groups and most of the larger sequence contigs end in R-regions, consistent with the expectation that most of the unmapped and unassembled parts of the genome are repeat-rich R-type sequence.

From earlier analysis of Chromosome 1 [17] and from analysis of markers on the largest two contigs of the 2007 assembly, it is obvious that P-regions have a higher density of good markers than R-regions [Figure 1; Figure 2. This is due largely to the non-unique nature of much of the R-region sequence (leading to multi-copy markers), and probably also to abundant simple-sequence repeats, which we find interfere with amplification of adjoining non-repetitive sequence.

The 661 HAPPY markers which fall into linkage groups cover ~31.0 Mb of the genome, giving an average spacing on mapped regions of ~46.9 kb. There are 105 singletons (unlinked HAPPY markers) in the integrated map. Assuming that these are distributed evenly across the unmapped areas, their average spacing is approximately 276 kb. This is more than double the range of the mapping panel (~100 kb), explaining why they do not link to other markers.

### Copy-number variation between strains of *E. tenella*

Although HAPPY mapping reveals an abundance of multi-copy sequences (which were represented only once in the assembly), the exact copy-numbers of these sequences in the genome is still unknown. In humans, multi-copy regions tend to vary in copy-number between individuals [25]. Furthermore, structural variations have never been studied in the *E. tenella* genome. Hence, we set out to investigate the HAPPY markers for variation in copy-number between strains of *E. tenella* using the molecular copy-number counting (MCC) method. We randomly selected 48 of the HAPPY markers for this analysis.

**Figure 3:**
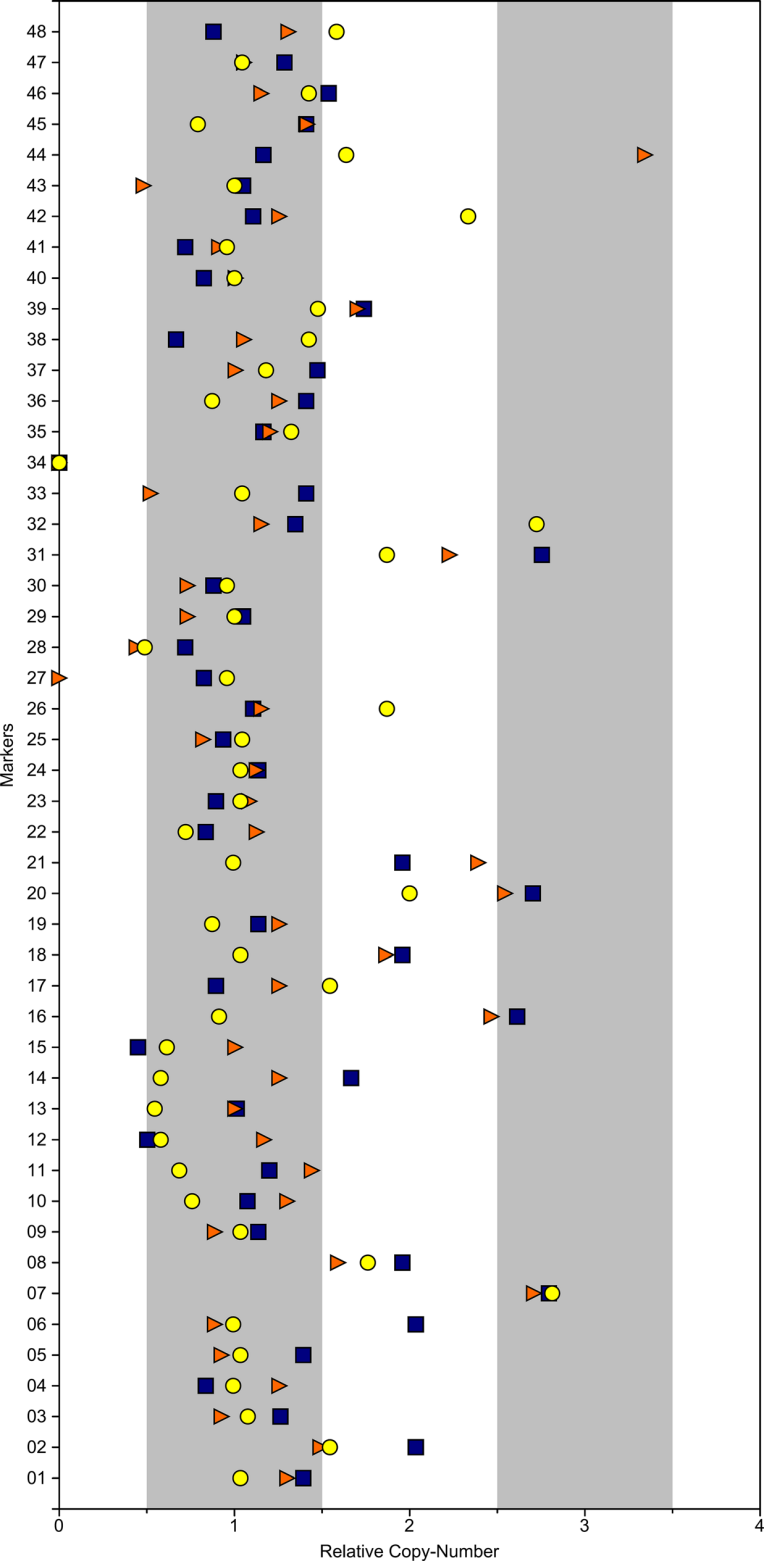
**Copy-number variations between*Eimeria tenella*strains.** For 48 markers (numbered at left) relative copy-number was measured on the Houghton (blue squares), Wisconsin (red triangles) and Weybridge (yellow circles) strains, as described in the text.

Of 48 markers tested on the reference Houghton (H) strain, one marker failed to amplify for unknown reasons. Twelve markers (25%) were detected to be present in multiple copies (relative copy-number > 1.5) in the genome [Figure 3]. Four of these twelve markers are present in more than two copies per haploid genome.

The analysis carried out on the Weybridge (Wey) strain also revealed eleven of the markers to be multi-copy while analysis on the Wisconsin (Wis) strain showed only nine multi-copy markers. Although the Wey strain had almost the same number of multi-copy markers as the H strain, most of them were lower in copy-number. The Wis strain has the lowest number of multi-copy markers but one of them (marker 44) is present in triple the normal copy-number. One marker, marker 27, did not show amplification in the Wis strain.

Overall, four markers were multi-copy in all three strains while sixteen markers were found to have differences in copy-number between the strains. We therefore estimate that, within any one strain, about 20% of sequences (other than short tandem repeats) are present in multiple copies (11, 12 and 9 out of the 47 markers, in the three strains analyzed), if our selection of markers is representative. Moreover, about a third of sequences (again, assuming our markers to be representative) differ in copy-number between strains. Further work is needed to clarify the extent of the duplicated sequence blocks, and their possible relation to genes.

## Discussion

The analysis of the Chromosome 1 sequence [17] showed a striking segmentation, with feature-poor (P) regions alternating with feature-rich (R) regions. The P-regions are slightly longer, have a higher and more uniform AT content than the R-regions and are almost free of simple repeats. In contrast, R-regions contain an abundance of simple repeats including tandemly repeated trinucleotides, the telomere-like AGGGTTT heptamer, the TGCATGCA palindromic octamer (which seems to be peculiar to a subset of apicomplexans; [26]), and also LINE transposons. It was speculated that the rest of the genome of *E. tenella* was organized in a similar segmented fashion.

The integrated map described here has revealed the structure for about half of the *E. tenella* genome based on the relative physical size of the map in comparison with the estimated genome size (60 Mb). Our analysis suggests that this half consists mainly of P-type sequence, largely because markers in R-regions are usually multicopy and therefore unmappable. If most R-regions are larger than 140 kb (the smallest R-region on Chromosome 1), then this would explain the inability of the map to connect across most R regions by means of HAPPY linkage or BAC clones.

Regarding the accuracy of the integrated map, we do see some conflicts in marker order when compared with the sequence assembly (see, for example, Additional file 4). For the most part, these are local inversions in line with the expected resolution of the HAPPY map; others, particularly where they involve small isolated sequence contigs, are likely to represent errors in the sequence assembly. As with all complex genomes, mapping and sequencing are iterative and interdependent processes, and there is no universally agreed metric for measuring the goodness of a map, nor agreement on how to weight common local errors against rarer large-scale errors. However, it is generally agreed that maps which integrate several datasets (in this case, HAPPY data, BAC-end data and contig data) are more robust than those which depend on a single method.

If we are correct in assuming that most of the unmappable multicopy markers originate from R-regions, this suggests that the R-regions are rich in repeated sequences (in addition to the simple-sequence repeats which are avoided in marker selection), and that a segment from an R-region on one chromosome may be duplicated at an R-region of another chromosome. About 20% of genome may be repeated in this way, in addition to the 14% made up of simple-sequence repeats (based on Chromosomes 1 and 2). This may account for the fact that the draft sequence assembly represents only about 78% of the genome.

Comparisons between *E. tenella* strains are scarce but have generally shown moderate variation at the sequence level. For instance, the recent comparison of the ~9 kb glucose-6-phosphate isomerase genomic locus between the H, Wis and Wey strains revealed 33 SNPs and 14 indels [19], or a variation of around half of one percent of nucleotides. In contrast, our preliminary study based on CNV analysis showed the possibility of much more widespread structural variation between these three strains.

## Conclusions

The integrated HAPPY map has revealed the probable structure of the *E. tenella* genome, and explains why the ongoing sequencing program has encountered difficulties. It suggests that the genome is architectured segmentally, alternating between P- and R-regions, with an average of about four or five P-regions per Mb. R-regions are likely to contain tracts of repeated sequence amounting to >20% of the genome, as well as a further 14% of simple-sequence repeats. This segmental structure and the repetitive nature of the R-segments probably explain both the gaps in the integrated map and the incompleteness of the draft sequence assembly.

There are also indications that much of the genome displays copy-number variation between *E. tenella* strains. Given that parasites must constantly adapt to oppose emerging resistance in the host, it is tempting to speculate that the segmental architecture contributes to a structurally dynamic genome, lubricated by the repeat-rich R-regions. This, in turn, may play an important role in the rapid emergence of drug resistance which is known to be a feature of *Eimeria*.

## Methods

### HAPPY mapping

The HAPPY mapping was performed essentially as described previously [26-28] on a mapping panel containing fragments selected at approximately 100 kb, with customization in the primer design and marker selection process. The draft genome assembly contigs were divided into fragments of ~2 kb in length. All of these 2 kb fragments were subjected to primer design using UniversalPrimerDesigner (Paul H. Dear, unpublished). This software was set to find the optimum set of hemi-nested primers (forward external, forward internal and reverse) for each 2 kb fragment. The designed primers had a predicted melting temperature of 55–62°C, with two G or C nucleotides at the 3’ end and one G or C nucleotide at the 5’ end, and length 18–22 bases. Each set of primers was designed to produce an internal amplicon of 100–200 bp (external amplicon length 150–350 bp) with an A + T content not exceeding 80%. Contigs similar to the Chromosome 1 and 2 sequences were filtered. The uniqueness of each primer set was then checked against the genome assembly. Markers were picked at contig ends for contigs larger than 17 kb, and one marker for contigs smaller than 17 kb. Where markers failed (for example, were found to be multi-copy), further markers were chosen from nearby sequences in an attempt to find successful markers.

### P/R region assignment

Contigs larger than 5 kb were divided into P- and R-segments using custom software, (Paul H. Dear, unpublished). Briefly, all of the simple-sequence repeats (of [CAG]n or [AGGGTTT]n, where n ≥ 3) which pepper the R-regions were identified, and both they and any intervening stretches of sequence shorter than 4 kb were marked as putative R-segments, with the remainder (stretches of >4 kb lacking simple-sequence repeats) being marked as putative P-segments. Based on analysis of Chromosomes 1 and 2, we assumed that genuine P- or R-segments would each be larger than 30 kb. Therefore, the shortest putative P- or R-segment was eliminated (so that, for example, a 5 kb putative R-region flanked by larger P-regions would be merged to become a single contiguous P-region); this was done iteratively until no segment smaller than 30 kb remained, apart from the first and last segments of the contig.

Contigs smaller than 5 kb were not divided into segments, but were instead classified as entirely P-type, R-type or unclassifiable based on their overall content of simple-sequence motifs (less than 0.5 motifs, more than 1 motif, or between 0.5 and 1 motifs per kilobase, respectively).

The parameters for this analysis are somewhat arbitrary, but were chosen such that they accurately identified the P/R segmentation that had been previously noted for Chromosomes 1 and 2. Moreover, slight variation in these parameters, or the use of analyses based on sequence information content and base composition (which also distinguish P- from R-segments) gave essentially similar results (not shown).

### Data integration

BAC-end sequences were mapped to the draft genome using ssahaEST [29] then processed to remove unpaired sequences and sequences that did not map uniquely to a single region. For the chromosomal assignment of map segments, HAPPY markers were designed for fifteen chromosomally-assigned genes and mapped as described.

### Copy-number variation analysis

Forty-eight randomly selected markers (as described in primer design) were typed on panels of sub-genomic aliquots of sheared DNA from three different *E. tenella* strains (Houghton, Weybridge and Wisconsin) using the MCC approach [21]. Panels of 96 aliquots containing approximately 0.3 genomes of sheared DNA were constructed for each strain to detect up to 3-fold change in copy-number variation. The selected markers were then typed using similar hemi-nested PCR following the same protocol as for the HAPPY mapping. The proportion of aliquots positive for any marker allows one to calculate its abundance in the panel (Poisson distribution), and hence its copy-number relative to other markers. Further details are given in reference [21].

## Competing interests

The authors declare that they have no competing interests.

## Authors' contributions

K-LW and PHD conceptualized the research plan. L-SL constructed the HAPPY map, and together with Y-LT, HA and PHD analyzed the data. L-SL drafted the manuscript. K-LW and PHD critically revised the manuscript. K-LW and PHD supervised and coordinated the study. All authors read and approved the final manuscript.

## Supplementary Material

Additional file 1 **Marker typing results according to stages.** Summary of marker typing results for the initial (“First”) set of HAPPY markers, for redesigned markers (“Second” and “Third”), for markers designed against chromosomally-assigned genes (“Chr”), and for all markers combined ("Overall"). Based on the average DNA content of each aliquot in the mapping panel, and on the Poisson distribution, we expect a single-copy sequence to be represented in 30-55 of the aliquots (mean ±2 s.d.); markers in this range are classified as good (green). Markers giving <30 positives, probably due to poor amplification, are classified as low copy (yellow), whilst those giving >55 positives are presumed to represent multi-copy sequences (blue). Markers that did not amplify are considered failed (red). Click here for file

Additional file 2 **Details of *Eimeria tenella *HAPPY map markers.** List of *Eimeria tenella* HAPPY map markers together with relevant details including mapping information, and primers and amplicon sequences. Click here for file

Additional file 3 **Analysis of BAC-end sequences.** Summary of results of BAC-end sequence analysis. Click here for file

Additional file 4 **Alignment of the integrated HAPPY map with WGS assemblies of the *E. tenella*genome.** Graphical representation of the alignment of the HAPPY map and sequence of the *Eimeria tenella* draft genome assembly contigs. For each map segment, labels “Cxxxxxxxx” are contig numbers in the 2007 assembly; labels “SCxx.y” indicate the supercontig (xx) and contig (y) of the 2010 assembly; HAPPY markers are named as “Ewgxxxxxx”; asterisks in 2010 assembly denote markers that mapped to more than one locus in the assembly. Click here for file

Additional file 5 **The comparison of the HAPPY map and sequence of (A) contig _00031646 and (B) contig_00031359.** Graphical representation of the alignment of the HAPPY map and sequence of the two largest *Eimeria tenella* draft genome assembly contigs. Click here for file
