# Supplementary figures and images for: Insights into the genome structure and copy-number variation of Eimeria tenella
Source: BMC Genomics. 2012 Aug 13;13:389. doi: 10.1186/1471-2164-13-389 (PMC3505466; doi:10.1186/1471-2164-13-389)

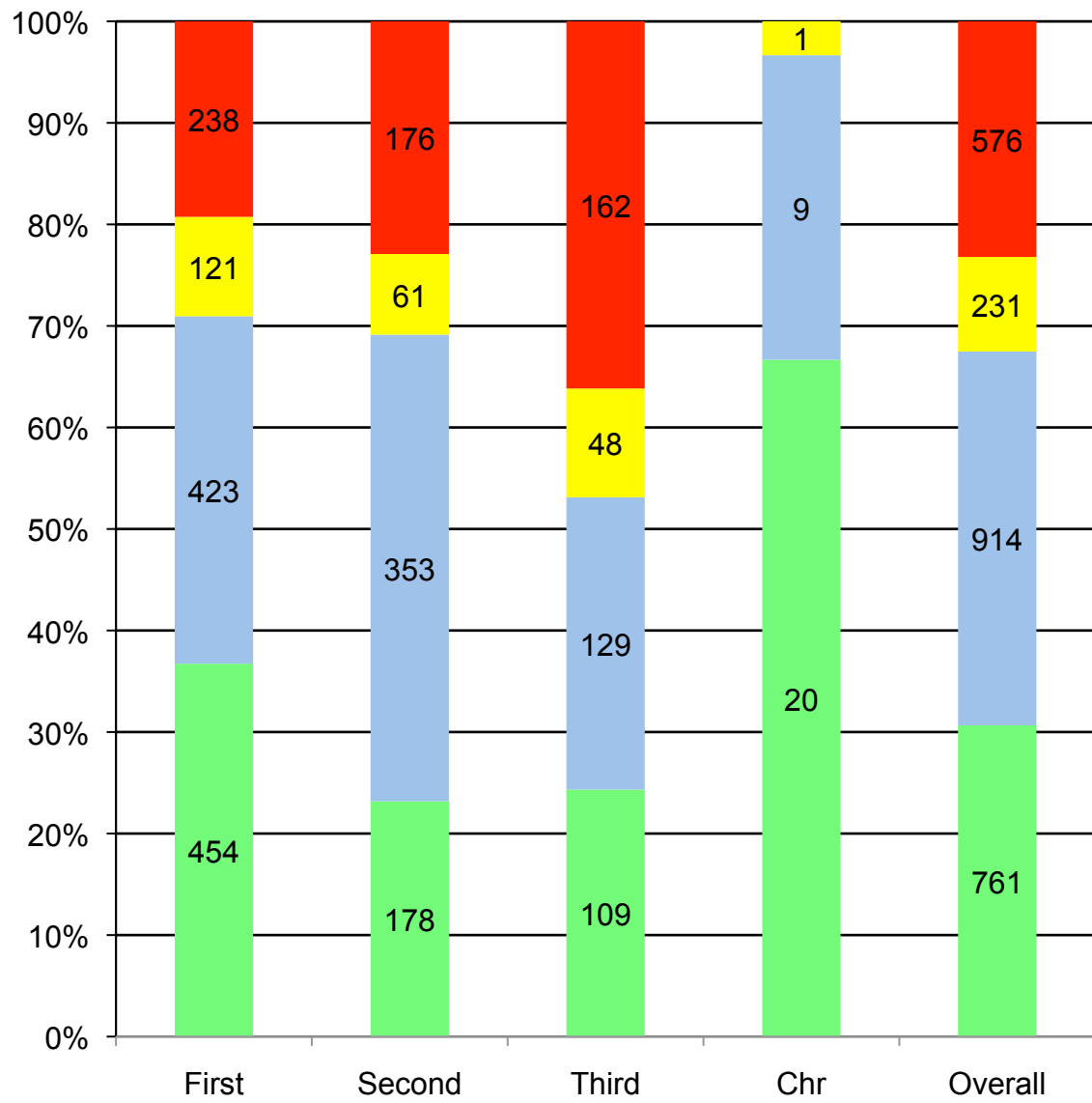

Supplement: Additional file 1 — Marker typing results according to stages. Summary of marker typing results for the initial (“First”) set of HAPPY markers, for redesigned markers (“Second” and “Third”), for markers designed against chromosomally-assigned genes (“Chr”), and for all markers combined ("Overall"). Based on the average DNA content of each aliquot in the mapping panel, and on the Poisson distribution, we expect a single-copy sequence to be represented in 30-55 of the aliquots (mean ±2 s.d.); markers in this range are classified as good (green). Markers giving <30 positives, probably due to poor amplification, are classified as low copy (yellow), whilst those giving >55 positives are presumed to represent multi-copy sequences (blue). Markers that did not amplify are considered failed (red). [file 1471-2164-13-389-S1.pdf]

A

HAPPY Map

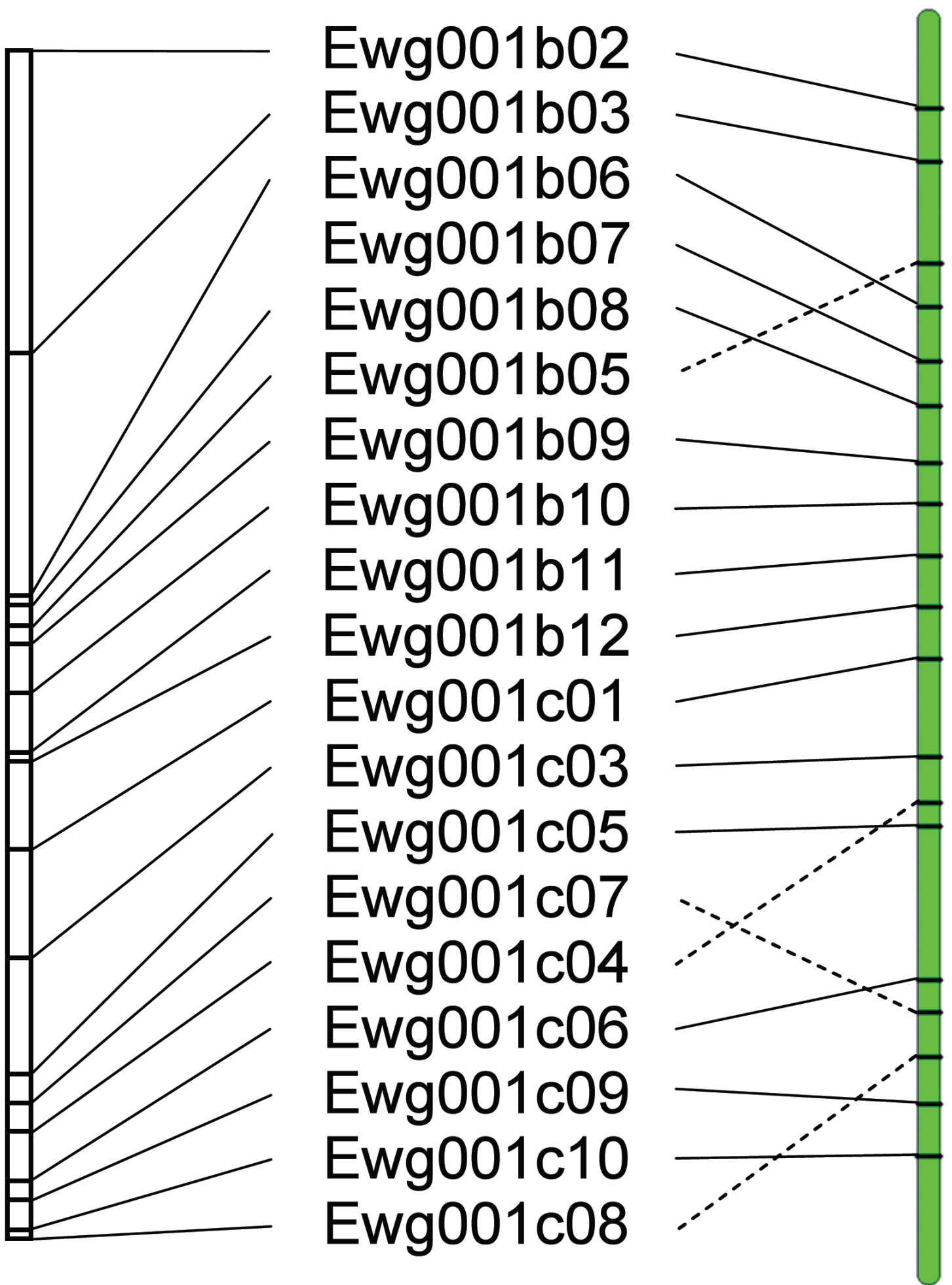

Contig\_00031646

B

HAPPY Map

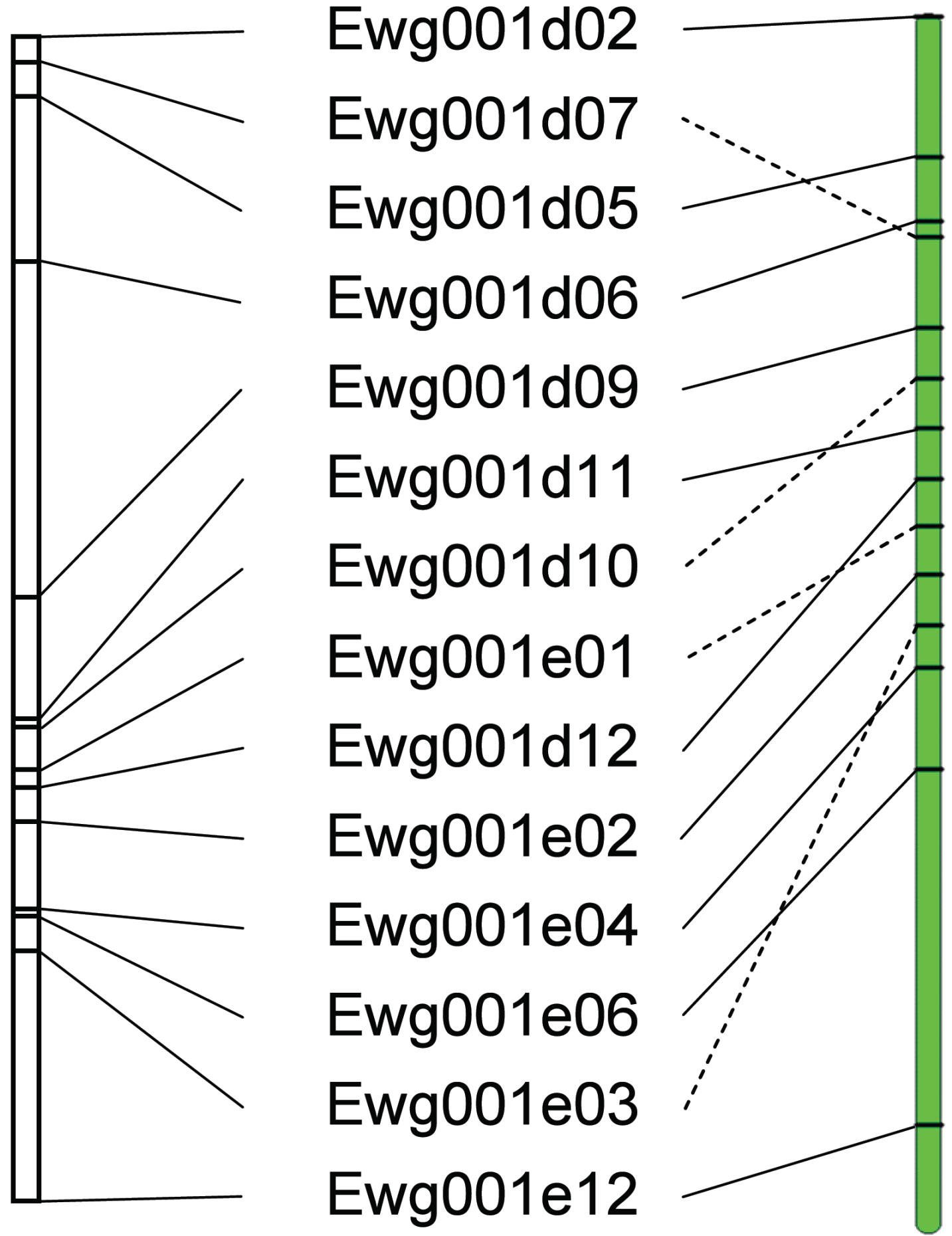

Contig\_00031359

Supplement: Additional file 5 — The comparison of the HAPPY map and sequence of (A) contig _00031646 and (B) contig_00031359. Graphical representation of the alignment of the HAPPY map and sequence of the two largest Eimeria tenella draft genome assembly contigs. [file 1471-2164-13-389-S5.pdf]
