# Supplementary material for: Insights into the genome structure and copy-number variation of Eimeria tenella
Source: BMC Genomics. 2012 Aug 13;13:389. doi: 10.1186/1471-2164-13-389 (PMC3505466; doi:10.1186/1471-2164-13-389)
Supplement: Additional file 3 — Analysis of BAC-end sequences. Summary of results of BAC-end sequence analysis. [file 1471-2164-13-389-S3.pdf]

Additional file 3. Analysis of BAC-end sequences.

Total BAC-end reads aligned >200bp on contigs of genome assembly	8917
Total BAC-end reads with uniquely aligned to a contig	8851
Number of BAC paired ends with unique alignment	3124
Number of BAC paired ends that aligned to different contig	2514
Total contigs involved	1948
Total size of contig involved (bp)	35,817,858
Number of unique linkage	2138
Number of unique linkage supported by at least two BACs	277
