# Supplementary material for: Insights into the genome structure and copy-number variation of Eimeria tenella
Source: BMC Genomics. 2012 Aug 13;13:389. doi: 10.1186/1471-2164-13-389 (PMC3505466; doi:10.1186/1471-2164-13-389)
Supplement: Additional file 4 — Alignment of the integrated HAPPY map with WGS assemblies of the E. tenellagenome. Graphical representation of the alignment of the HAPPY map and sequence of the Eimeria tenella draft genome assembly contigs. For each map segment, labels “Cxxxxxxxx” are contig numbers in the 2007 assembly; labels “SCxx.y” indicate the supercontig (xx) and contig (y) of the 2010 assembly; HAPPY markers are named as “Ewgxxxxxx”; asterisks in 2010 assembly denote markers that mapped to more than one locus in the assembly. [file 1471-2164-13-389-S4.pdf]

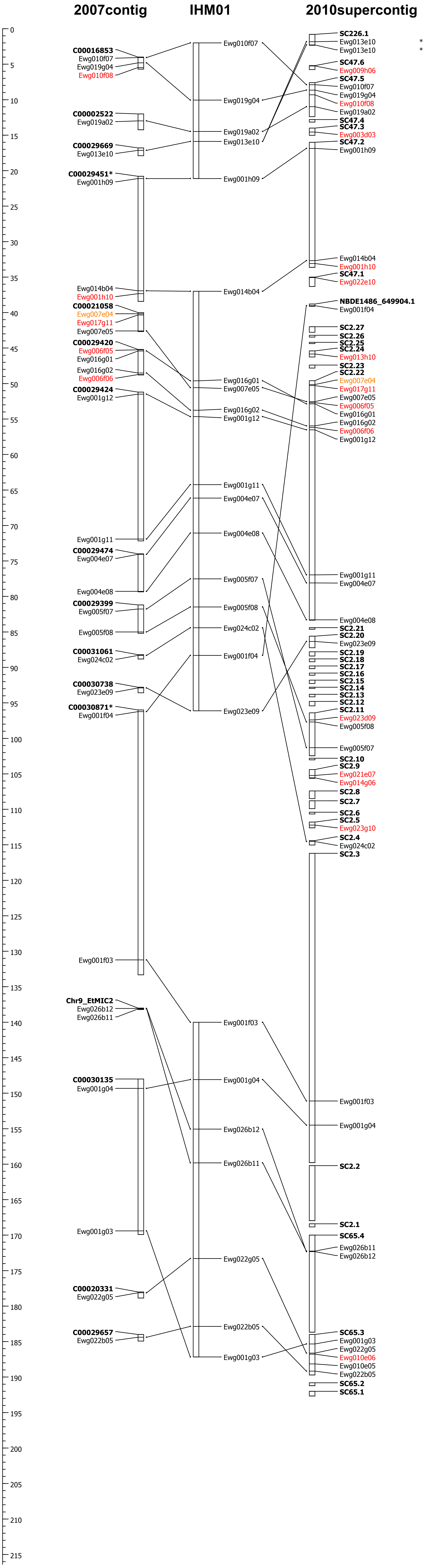
2007contig
IHM01
2010supercontig
0 5 10 15 20 25 30 35 40 45 50 55 60 65 70 75 80 85 90 95 100 105 110 115 120 125 130 135 140 145 150 155 160 165 170 175 180 185 190 195 200 205 210 215
C00016853
Ewg010f07
Ewg019g04
Ewg010f08
C00002522
Ewg019a02
C00029669
Ewg013e10
C00029451*
Ewg001h09
Ewg014b04
Ewg001h10
C00021058
Ewg007e04
Ewg017g11
Ewg007e05
C00029420
Ewg006f05
Ewg016g01
Ewg016g02
Ewg006f06
C00029424
Ewg001g12
Ewg001g11
C00029474
Ewg004e07
Ewg004e08
C00029399
Ewg005f07
Ewg005f08
C00031061
Ewg024c02
C00030738
Ewg023e09
C00030871*
Ewg001f04
Ewg001f03
Chr9_EtMIC2
Ewg026b12
Ewg026b11
C00030135
Ewg001g04
Ewg001g03
C00020331
Ewg022g05
C00029657
Ewg022b05
Ewg010f07
Ewg019g04
Ewg019a02
Ewg013e10
Ewg001h09
Ewg014b04
Ewg016g01
Ewg007e05
Ewg016g02
Ewg001g12
Ewg001g11
Ewg004e07
Ewg004e08
Ewg005f07
Ewg005f08
Ewg024c02
Ewg001f04
Ewg023e09
Ewg001f03
Ewg001g04
Ewg026b12
Ewg026b11
Ewg022g05
Ewg022b05
Ewg001g03
SC226.1
Ewg013e10 *
Ewg013e10 *
SC47.6
Ewg009h06
SC47.5
Ewg010f07
Ewg019g04
Ewg010f08
Ewg019a02
SC47.4
SC47.3
Ewg003d03
SC47.2
Ewg001h09
Ewg014b04
Ewg001h10
SC47.1
Ewg022e10
NBDE1486_649904.1
Ewg001f04
SC2.27
SC2.26
SC2.25
SC2.24
Ewg013h10
SC2.23
SC2.22
Ewg007e04
Ewg017g11
Ewg007e05
Ewg006f05
Ewg016g01
Ewg016g02
Ewg006f06
Ewg001g12
Ewg001g11
Ewg004e07
Ewg004e08
SC2.21
SC2.20
Ewg023e09
SC2.19
SC2.18
SC2.17
SC2.16
SC2.15
SC2.14
SC2.13
SC2.12
SC2.11
Ewg023d09
Ewg005f08
Ewg005f07
SC2.10
SC2.9
Ewg021e07
Ewg014g06
SC2.8
SC2.7
SC2.6
SC2.5
Ewg023g10
SC2.4
Ewg024c02
SC2.3
Ewg001f03
Ewg001g04
SC2.2
SC2.1
SC65.4
Ewg026b11
Ewg026b12
SC65.3
Ewg001g03
Ewg022g05
Ewg010e06
Ewg010e05
Ewg022b05
SC65.2
SC65.1

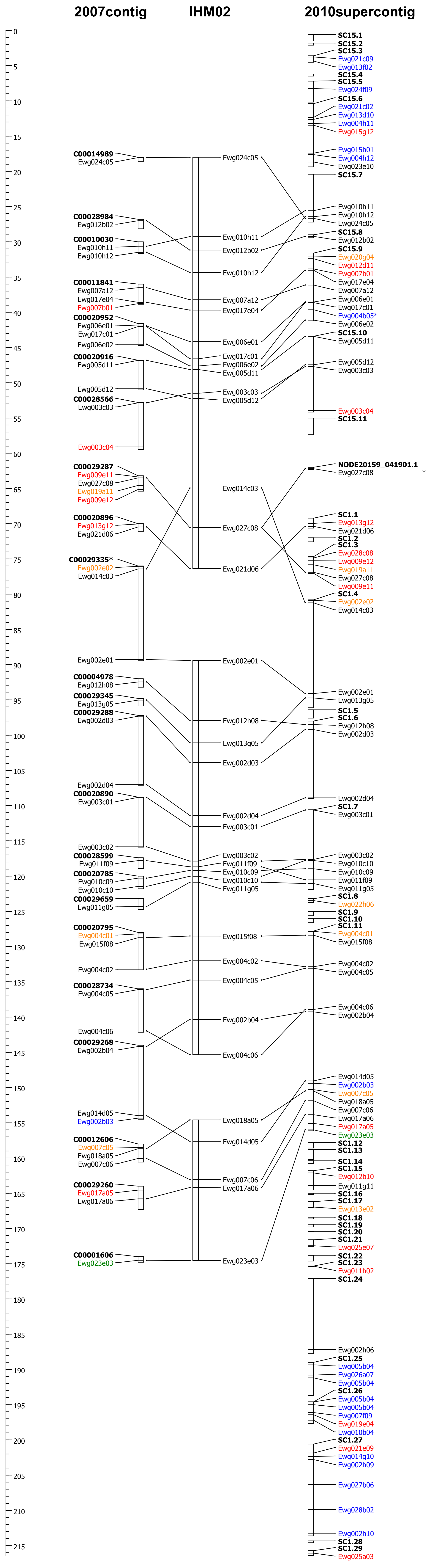
2007contig
IHM02
2010supercontig
C00014989
C00028984
C00010030
C00011841
C00020952
C00020916
C00028566
C00029287
C00020896
C00029335*
C00004978
C00029345
C00029288
C00020890
C00028599
C00020785
C00029659
C00020795
C00028734
C00029268
C00012606
C00029260
C00001606
NODE20159_041901.1

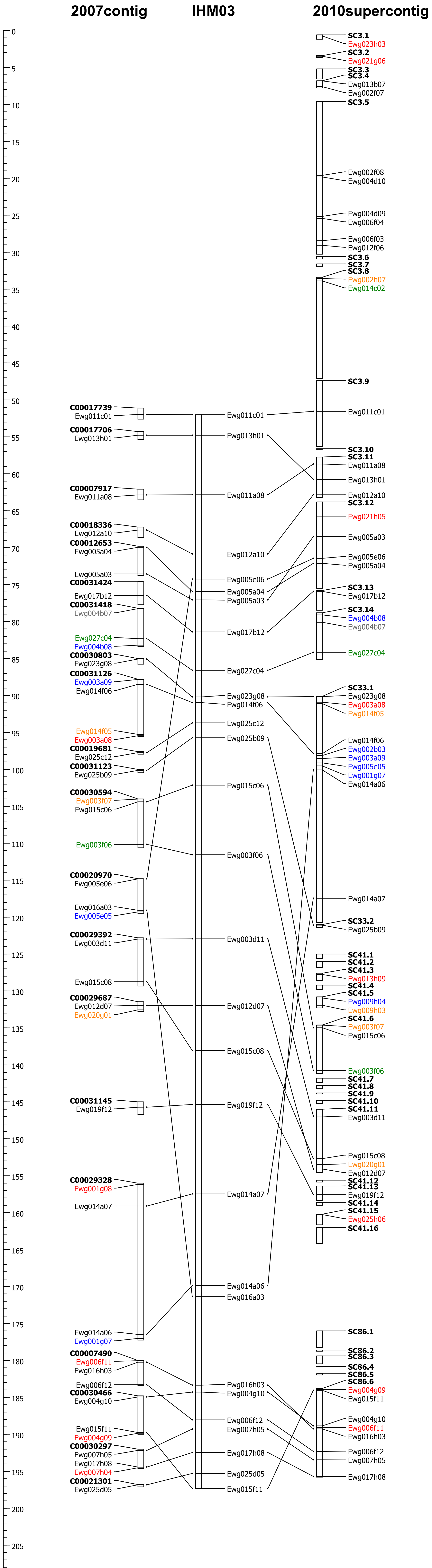

2007contig
IHM03
2010supercontig

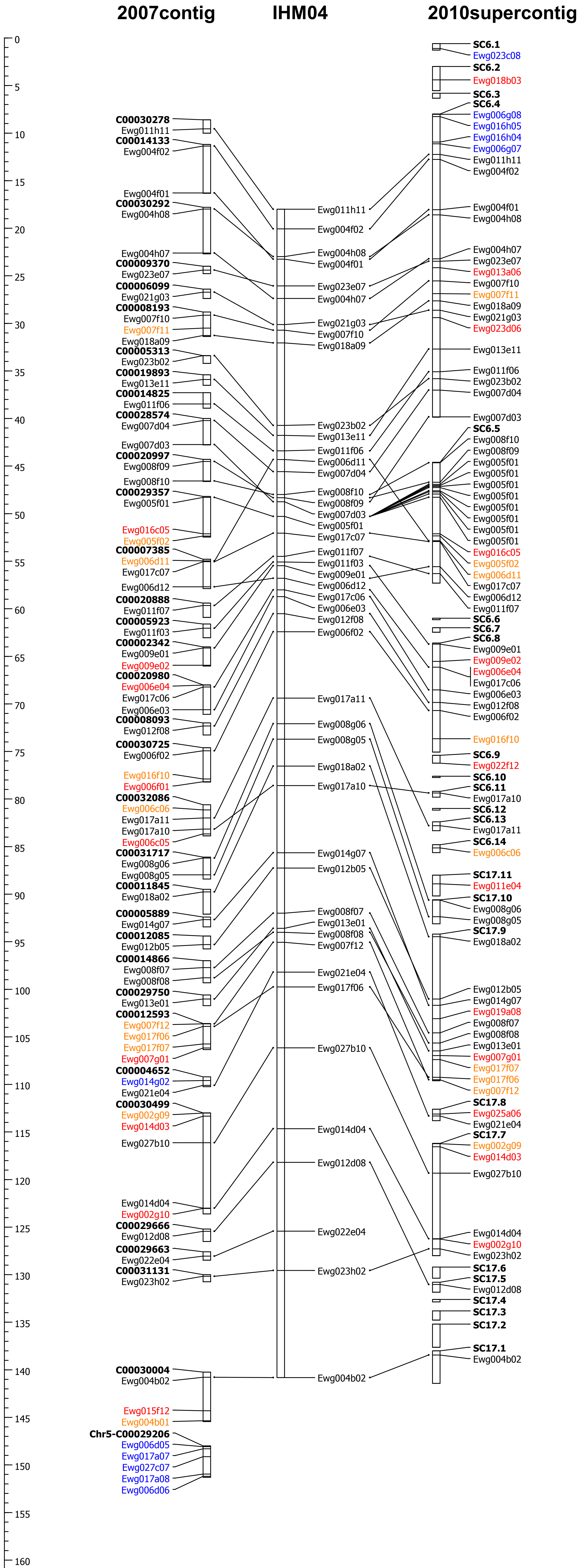

2007contig
IHM04
2010supercontig

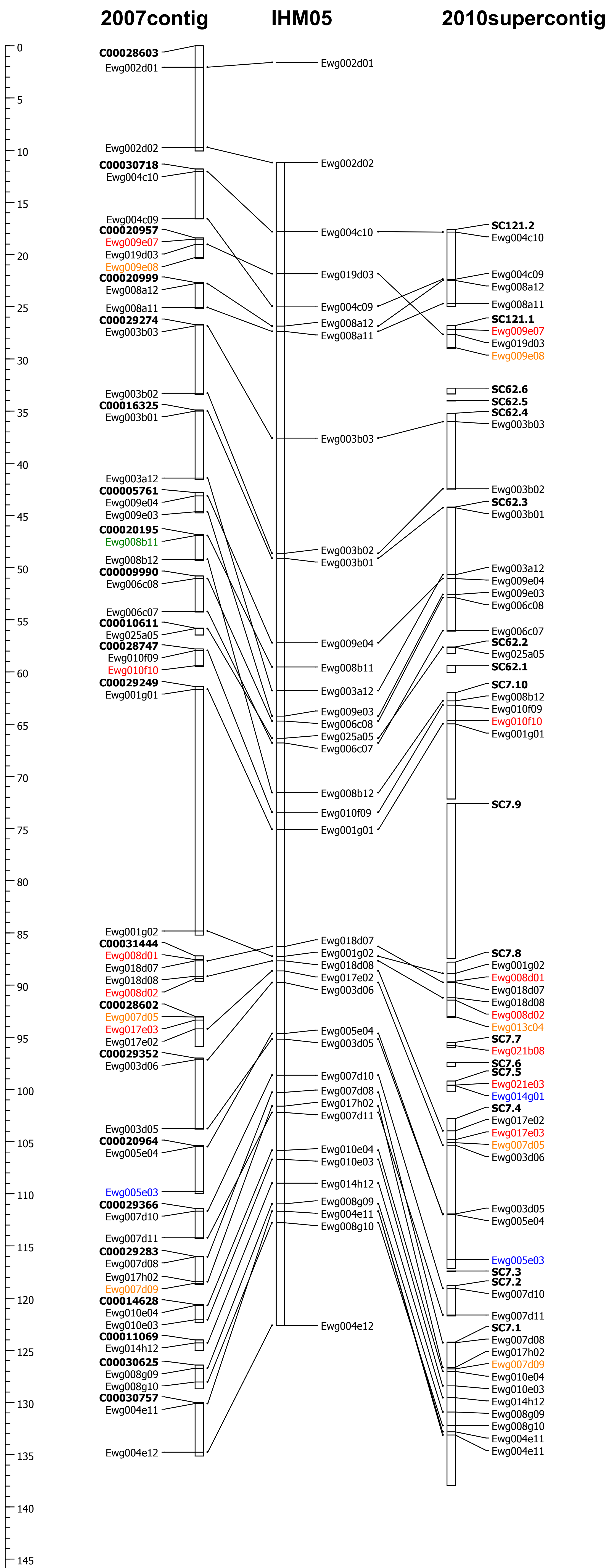
2007contig
IHM05
2010supercontig
C00028603
Ewg002d01
Ewg002d02
C00030718
Ewg004c10
Ewg004c09
C00020957
Ewg009e07
Ewg019d03
Ewg009e08
C00020999
Ewg008a12
Ewg008a11
C00029274
Ewg003b03
Ewg003b02
C00016325
Ewg003b01
Ewg003a12
C00005761
Ewg009e04
Ewg009e03
C00020195
Ewg008b11
Ewg008b12
C00009990
Ewg006c08
Ewg006c07
C00010611
Ewg025a05
C00028747
Ewg010f09
Ewg010f10
C00029249
Ewg001g01
Ewg001g02
C00031444
Ewg008d01
Ewg018d07
Ewg018d08
Ewg008d02
C00028602
Ewg007d05
Ewg017e03
Ewg017e02
C00029352
Ewg003d06
Ewg003d05
C00020964
Ewg005e04
Ewg005e03
C00029366
Ewg007d10
Ewg007d11
C00029283
Ewg007d08
Ewg017h02
Ewg007d09
C00014628
Ewg010e04
Ewg010e03
C00011069
Ewg014h12
C00030625
Ewg008g09
Ewg008g10
C00030757
Ewg004e11
Ewg004e12
SC121.2
SC121.1
SC62.6
SC62.5
SC62.4
SC62.3
SC62.2
SC62.1
SC7.10
SC7.9
SC7.8
Ewg013c04
SC7.7
Ewg021b08
SC7.6
SC7.5
Ewg021e03
Ewg014g01
SC7.4
SC7.3
SC7.2
SC7.1
0
5
10
15
20
25
30
35
40
45
50
55
60
65
70
75
80
85
90
95
100
105
110
115
120
125
130
135
140
145

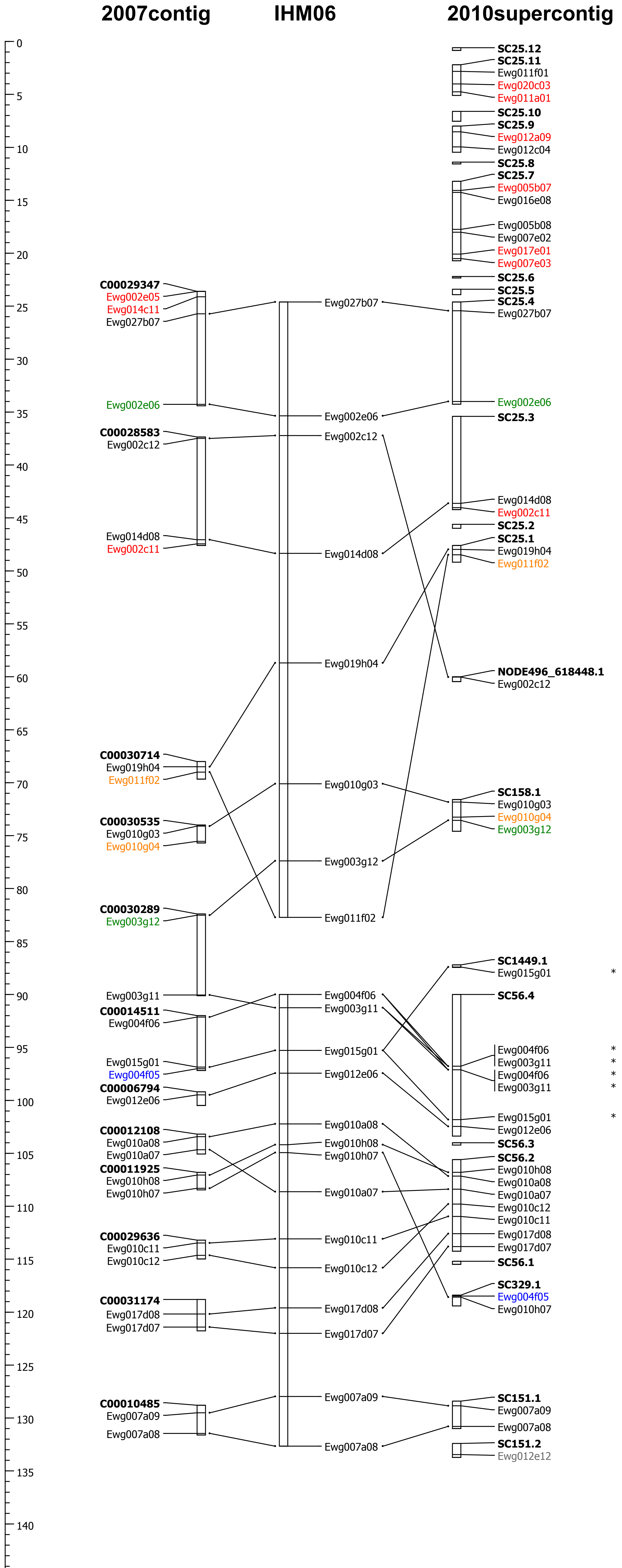

2007contig
IHM06
2010supercontig
0
5
10
15
20
25
30
35
40
45
50
55
60
65
70
75
80
85
90
95
100
105
110
115
120
125
130
135
140
C00029347
Ewg002e05
Ewg014c11
Ewg027b07
Ewg002e06
C00028583
Ewg002c12
Ewg014d08
Ewg002c11
C00030714
Ewg019h04
Ewg011f02
C00030535
Ewg010g03
Ewg010g04
C00030289
Ewg003g12
Ewg003g11
C00014511
Ewg004f06
Ewg015g01
Ewg004f05
C00006794
Ewg012e06
C00012108
Ewg010a08
Ewg010a07
C00011925
Ewg010h08
Ewg010h07
C00029636
Ewg010c11
Ewg010c12
C00031174
Ewg017d08
Ewg017d07
C00010485
Ewg007a09
Ewg007a08
Ewg027b07
Ewg002e06
Ewg002c12
Ewg014d08
Ewg019h04
Ewg010g03
Ewg003g12
Ewg011f02
Ewg004f06
Ewg003g11
Ewg015g01
Ewg012e06
Ewg010a08
Ewg010h08
Ewg010h07
Ewg010a07
Ewg010c11
Ewg010c12
Ewg017d08
Ewg017d07
Ewg007a09
Ewg007a08
SC25.12
SC25.11
Ewg011f01
Ewg020c03
Ewg011a01
SC25.10
SC25.9
Ewg012a09
Ewg012c04
SC25.8
SC25.7
Ewg005b07
Ewg016e08
Ewg005b08
Ewg007e02
Ewg017e01
Ewg007e03
SC25.6
SC25.5
SC25.4
Ewg027b07
Ewg002e06
SC25.3
Ewg014d08
Ewg002c11
SC25.2
SC25.1
Ewg019h04
Ewg011f02
NODE496_618448.1
Ewg002c12
SC158.1
Ewg010g03
Ewg010g04
Ewg003g12
SC1449.1
Ewg015g01 *
SC56.4
Ewg004f06 *
Ewg003g11 *
Ewg004f06 *
Ewg003g11 *
Ewg015g01 *
Ewg012e06
SC56.3
SC56.2
Ewg010h08
Ewg010a08
Ewg010a07
Ewg010c12
Ewg010c11
Ewg017d08
Ewg017d07
SC56.1
SC329.1
Ewg004f05
Ewg010h07
SC151.1
Ewg007a09
Ewg007a08
SC151.2
Ewg012e12

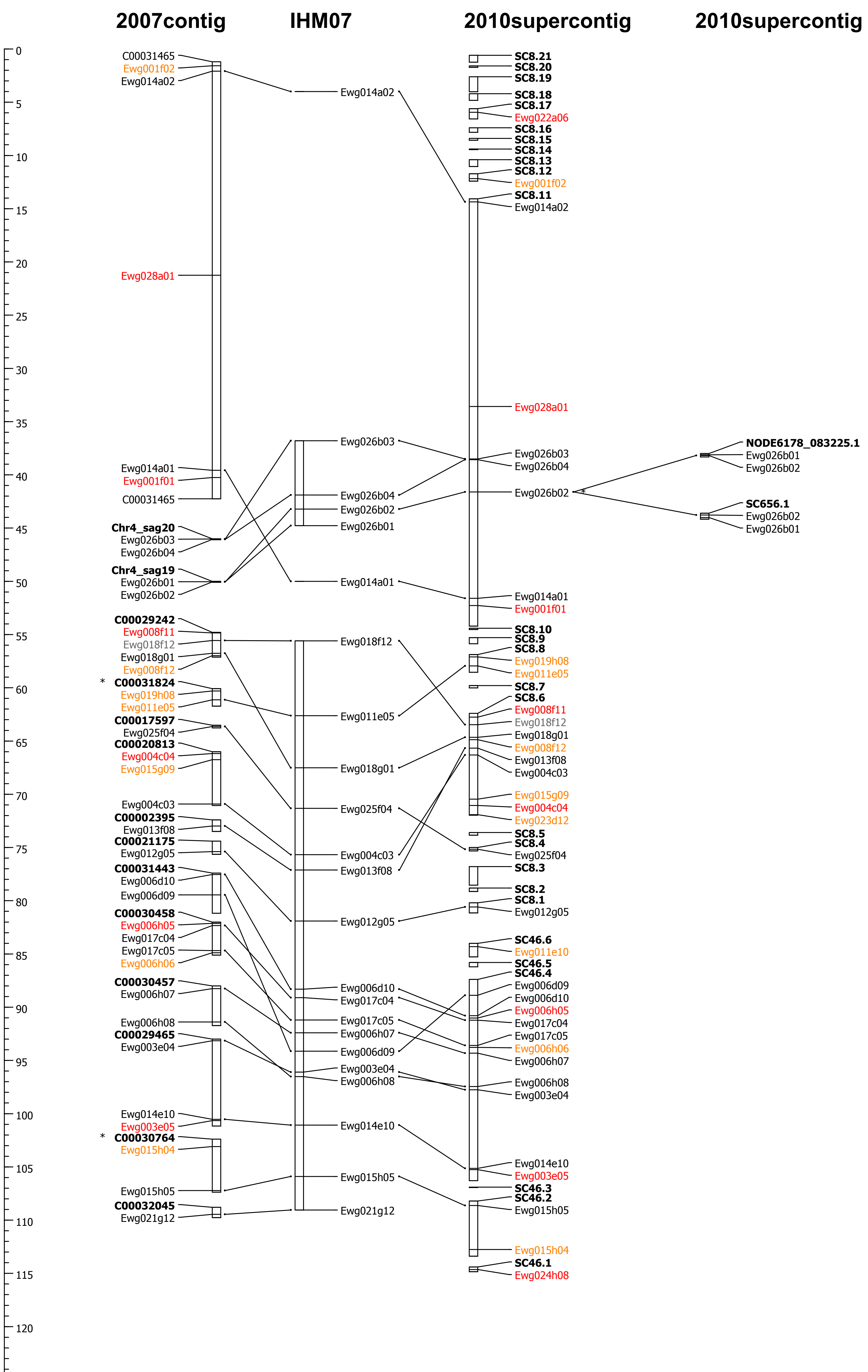

2007contig
IHM07
2010supercontig
2010supercontig
C00031465
Ewg001f02
Ewg014a02
Ewg028a01
Ewg014a01
Ewg001f01
C00031465
Chr4_sag20
Ewg026b03
Ewg026b04
Chr4_sag19
Ewg026b01
Ewg026b02
C00029242
Ewg008f11
Ewg018f12
Ewg018g01
Ewg008f12
C00031824
Ewg019h08
Ewg011e05
C00017597
Ewg025f04
C00020813
Ewg004c04
Ewg015g09
Ewg004c03
C00002395
Ewg013f08
C00021175
Ewg012g05
C00031443
Ewg006d10
Ewg006d09
C00030458
Ewg006h05
Ewg017c04
Ewg017c05
Ewg006h06
C00030457
Ewg006h07
Ewg006h08
C00029465
Ewg003e04
Ewg014e10
Ewg003e05
C00030764
Ewg015h04
Ewg015h05
C00032045
Ewg021g12
SC8.21
SC8.20
SC8.19
SC8.18
SC8.17
Ewg022a06
SC8.16
SC8.15
SC8.14
SC8.13
SC8.12
SC8.11
SC8.10
SC8.9
SC8.8
Ewg011e05
SC8.7
SC8.6
Ewg023d12
SC8.5
SC8.4
SC8.3
SC8.2
SC8.1
SC46.6
Ewg011e10
SC46.5
SC46.4
SC46.3
SC46.2
SC46.1
Ewg024h08
NODE6178_083225.1
SC656.1

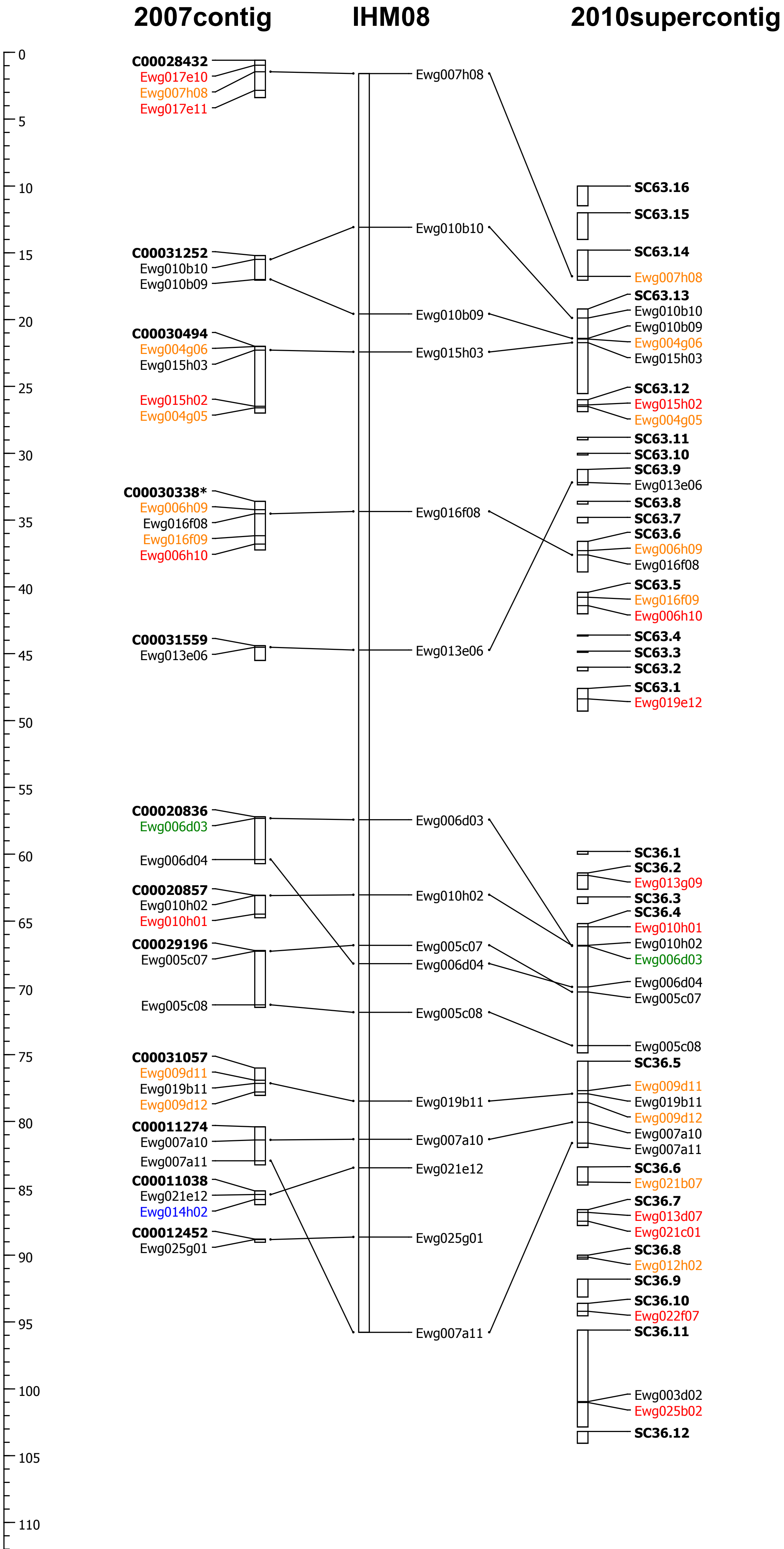
2007contig
IHM08
2010supercontig
0
5
10
15
20
25
30
35
40
45
50
55
60
65
70
75
80
85
90
95
100
105
110
C00028432
Ewg017e10
Ewg007h08
Ewg017e11
C00031252
Ewg010b10
Ewg010b09
C00030494
Ewg004g06
Ewg015h03
Ewg015h02
Ewg004g05
C00030338*
Ewg006h09
Ewg016f08
Ewg016f09
Ewg006h10
C00031559
Ewg013e06
C00020836
Ewg006d03
Ewg006d04
C00020857
Ewg010h02
Ewg010h01
C00029196
Ewg005c07
Ewg005c08
C00031057
Ewg009d11
Ewg019b11
Ewg009d12
C00011274
Ewg007a10
Ewg007a11
C00011038
Ewg021e12
Ewg014h02
C00012452
Ewg025g01
Ewg007h08
Ewg010b10
Ewg010b09
Ewg015h03
Ewg016f08
Ewg013e06
Ewg006d03
Ewg010h02
Ewg005c07
Ewg006d04
Ewg005c08
Ewg019b11
Ewg007a10
Ewg021e12
Ewg025g01
Ewg007a11
SC63.16
SC63.15
SC63.14
Ewg007h08
SC63.13
Ewg010b10
Ewg010b09
Ewg004g06
Ewg015h03
SC63.12
Ewg015h02
Ewg004g05
SC63.11
SC63.10
SC63.9
Ewg013e06
SC63.8
SC63.7
SC63.6
Ewg006h09
Ewg016f08
SC63.5
Ewg016f09
Ewg006h10
SC63.4
SC63.3
SC63.2
SC63.1
Ewg019e12
SC36.1
SC36.2
Ewg013g09
SC36.3
SC36.4
Ewg010h01
Ewg010h02
Ewg006d03
Ewg006d04
Ewg005c07
Ewg005c08
SC36.5
Ewg009d11
Ewg019b11
Ewg009d12
Ewg007a10
Ewg007a11
SC36.6
Ewg021b07
SC36.7
Ewg013d07
Ewg021c01
SC36.8
Ewg012h02
SC36.9
SC36.10
Ewg022f07
SC36.11
Ewg003d02
Ewg025b02
SC36.12

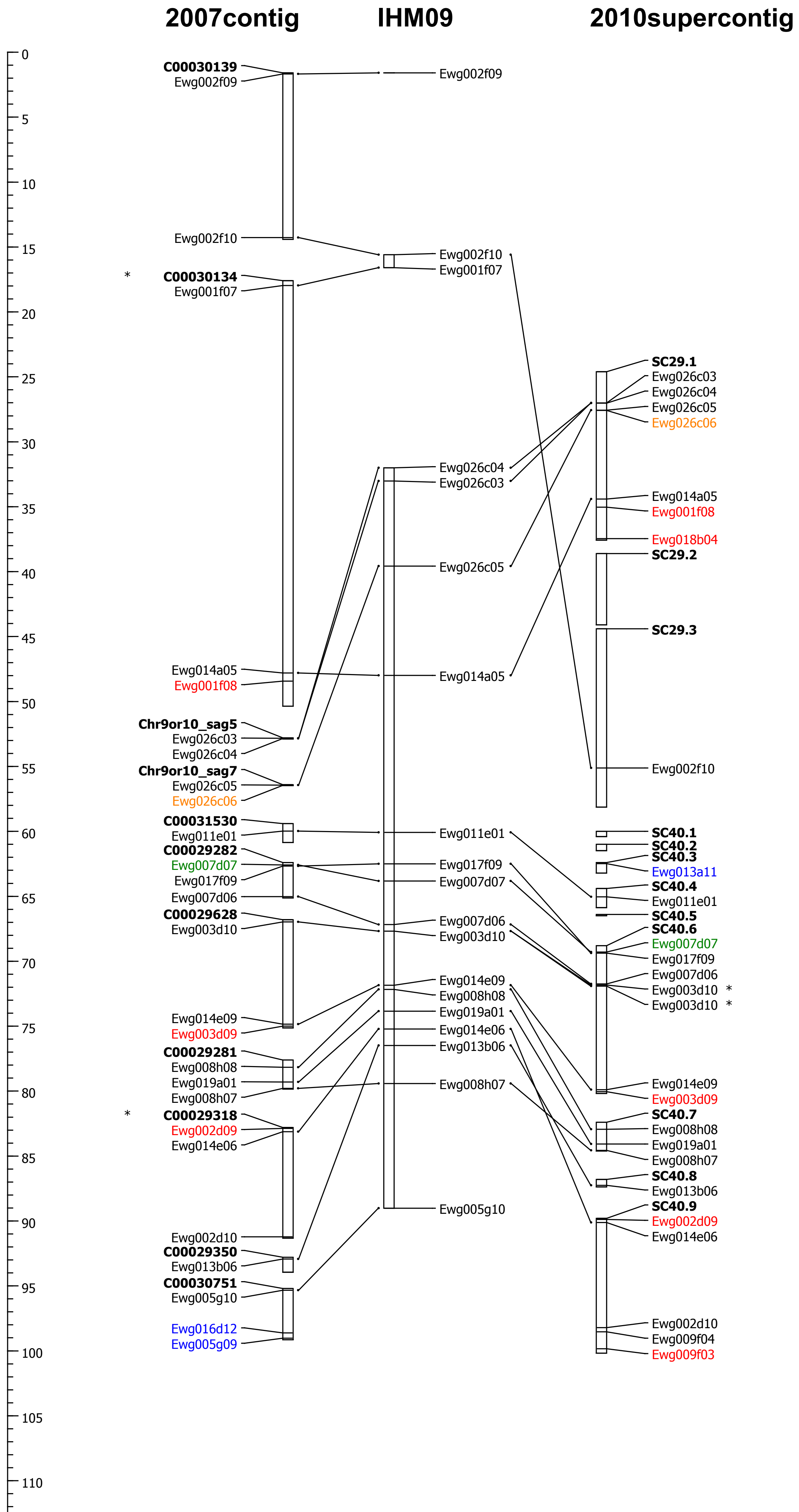

2007contig
IHM09
2010supercontig
C00030139
Ewg002f09
Ewg002f10
C00030134
Ewg001f07
Ewg014a05
Ewg001f08
Chr9or10_sag5
Ewg026c03
Ewg026c04
Chr9or10_sag7
Ewg026c05
Ewg026c06
C00031530
Ewg011e01
C00029282
Ewg007d07
Ewg017f09
Ewg007d06
C00029628
Ewg003d10
Ewg014e09
Ewg003d09
C00029281
Ewg008h08
Ewg019a01
Ewg008h07
C00029318
Ewg002d09
Ewg014e06
Ewg002d10
C00029350
Ewg013b06
C00030751
Ewg005g10
Ewg016d12
Ewg005g09
Ewg002f09
Ewg002f10
Ewg001f07
Ewg026c04
Ewg026c03
Ewg026c05
Ewg014a05
Ewg011e01
Ewg017f09
Ewg007d07
Ewg007d06
Ewg003d10
Ewg014e09
Ewg008h08
Ewg019a01
Ewg014e06
Ewg013b06
Ewg008h07
Ewg005g10
SC29.1
Ewg026c03
Ewg026c04
Ewg026c05
Ewg026c06
Ewg014a05
Ewg001f08
Ewg018b04
SC29.2
SC29.3
Ewg002f10
SC40.1
SC40.2
SC40.3
Ewg013a11
SC40.4
Ewg011e01
SC40.5
SC40.6
Ewg007d07
Ewg017f09
Ewg007d06
Ewg003d10 *
Ewg003d10 *
Ewg014e09
Ewg003d09
SC40.7
Ewg008h08
Ewg019a01
Ewg008h07
SC40.8
Ewg013b06
SC40.9
Ewg002d09
Ewg014e06
Ewg002d10
Ewg009f04
Ewg009f03
0
5
10
15
20
25
30
35
40
45
50
55
60
65
70
75
80
85
90
95
100
105
110

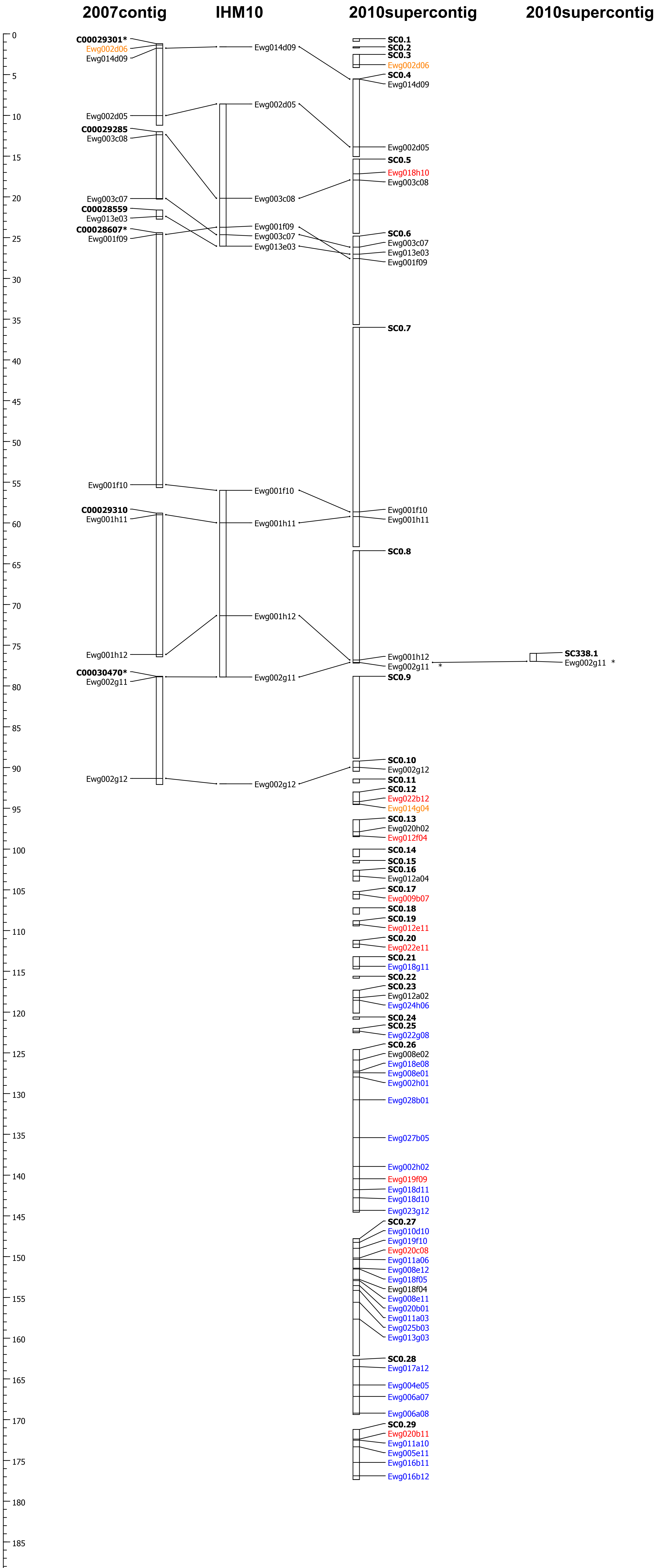
2007contig
IHM10
2010supercontig
2010supercontig
0
5
10
15
20
25
30
35
40
45
50
55
60
65
70
75
80
85
90
95
100
105
110
115
120
125
130
135
140
145
150
155
160
165
170
175
180
185
C00029301*
Ewg002d06
Ewg014d09
Ewg002d05
C00029285
Ewg003c08
Ewg003c07
C00028559
Ewg013e03
C00028607*
Ewg001f09
Ewg001f10
C00029310
Ewg001h11
Ewg001h12
C00030470*
Ewg002g11
Ewg002g12
Ewg014d09
Ewg002d05
Ewg003c08
Ewg001f09
Ewg003c07
Ewg013e03
Ewg001f10
Ewg001h11
Ewg001h12
Ewg002g11
Ewg002g12
SC0.1
SC0.2
SC0.3
Ewg002d06
SC0.4
Ewg014d09
Ewg002d05
SC0.5
Ewg018h10
Ewg003c08
SC0.6
Ewg003c07
Ewg013e03
Ewg001f09
SC0.7
Ewg001f10
Ewg001h11
SC0.8
Ewg001h12
Ewg002g11 *
SC0.9
SC0.10
Ewg002g12
SC0.11
SC0.12
Ewg022b12
Ewg014g04
SC0.13
Ewg020h02
Ewg012f04
SC0.14
SC0.15
SC0.16
Ewg012a04
SC0.17
Ewg009b07
SC0.18
SC0.19
Ewg012e11
SC0.20
Ewg022e11
SC0.21
Ewg018g11
SC0.22
SC0.23
Ewg012a02
Ewg024h06
SC0.24
SC0.25
Ewg022g08
SC0.26
Ewg008e02
Ewg018e08
Ewg008e01
Ewg002h01
Ewg028b01
Ewg027b05
Ewg002h02
Ewg019f09
Ewg018d11
Ewg018d10
Ewg023g12
SC0.27
Ewg010d10
Ewg019f10
Ewg020c08
Ewg011a06
Ewg008e12
Ewg018f05
Ewg018f04
Ewg008e11
Ewg020b01
Ewg011a03
Ewg025b03
Ewg013g03
SC0.28
Ewg017a12
Ewg004e05
Ewg006a07
Ewg006a08
SC0.29
Ewg020b11
Ewg011a10
Ewg005e11
Ewg016b11
Ewg016b12
SC338.1
Ewg002g11 *

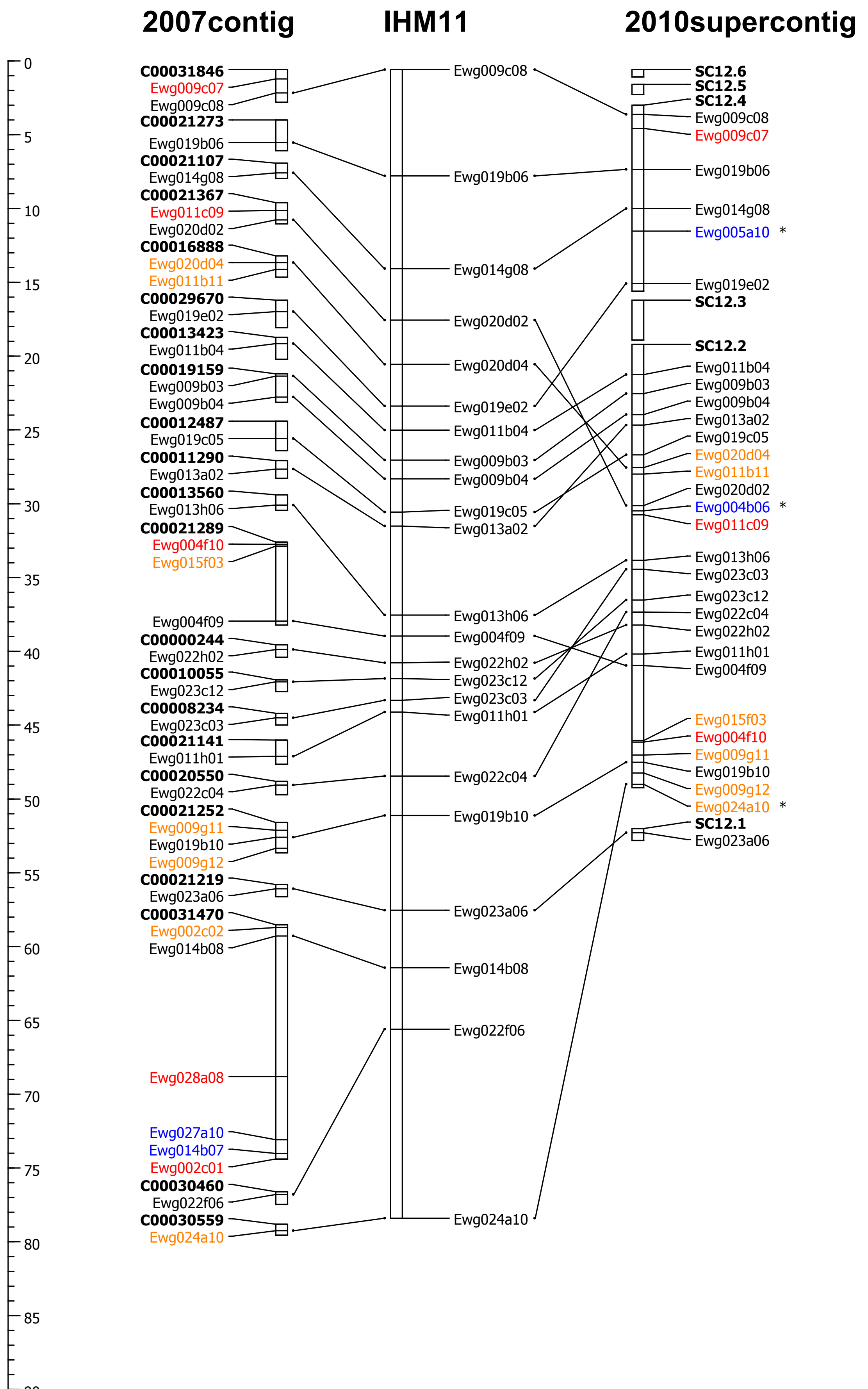

2007contig
IHM11
2010supercontig
0
5
10
15
20
25
30
35
40
45
50
55
60
65
70
75
80
85
C00031846
Ewg009c07
Ewg009c08
C00021273
Ewg019b06
C00021107
Ewg014g08
C00021367
Ewg011c09
Ewg020d02
C00016888
Ewg020d04
Ewg011b11
C00029670
Ewg019e02
C00013423
Ewg011b04
C00019159
Ewg009b03
Ewg009b04
C00012487
Ewg019c05
C00011290
Ewg013a02
C00013560
Ewg013h06
C00021289
Ewg004f10
Ewg015f03
Ewg004f09
C00000244
Ewg022h02
C00010055
Ewg023c12
C00008234
Ewg023c03
C00021141
Ewg011h01
C00020550
Ewg022c04
C00021252
Ewg009g11
Ewg019b10
Ewg009g12
C00021219
Ewg023a06
C00031470
Ewg002c02
Ewg014b08
Ewg028a08
Ewg027a10
Ewg014b07
Ewg002c01
C00030460
Ewg022f06
C00030559
Ewg024a10
Ewg009c08
Ewg019b06
Ewg014g08
Ewg020d02
Ewg020d04
Ewg019e02
Ewg011b04
Ewg009b03
Ewg009b04
Ewg019c05
Ewg013a02
Ewg013h06
Ewg004f09
Ewg022h02
Ewg023c12
Ewg023c03
Ewg011h01
Ewg022c04
Ewg019b10
Ewg023a06
Ewg014b08
Ewg022f06
Ewg024a10
SC12.6
SC12.5
SC12.4
Ewg009c08
Ewg009c07
Ewg019b06
Ewg014g08
Ewg005a10 *
Ewg019e02
SC12.3
SC12.2
Ewg011b04
Ewg009b03
Ewg009b04
Ewg013a02
Ewg019c05
Ewg020d04
Ewg011b11
Ewg020d02
Ewg004b06 *
Ewg011c09
Ewg013h06
Ewg023c03
Ewg023c12
Ewg022c04
Ewg022h02
Ewg011h01
Ewg004f09
Ewg015f03
Ewg004f10
Ewg009g11
Ewg019b10
Ewg009g12
Ewg024a10 *
SC12.1
Ewg023a06

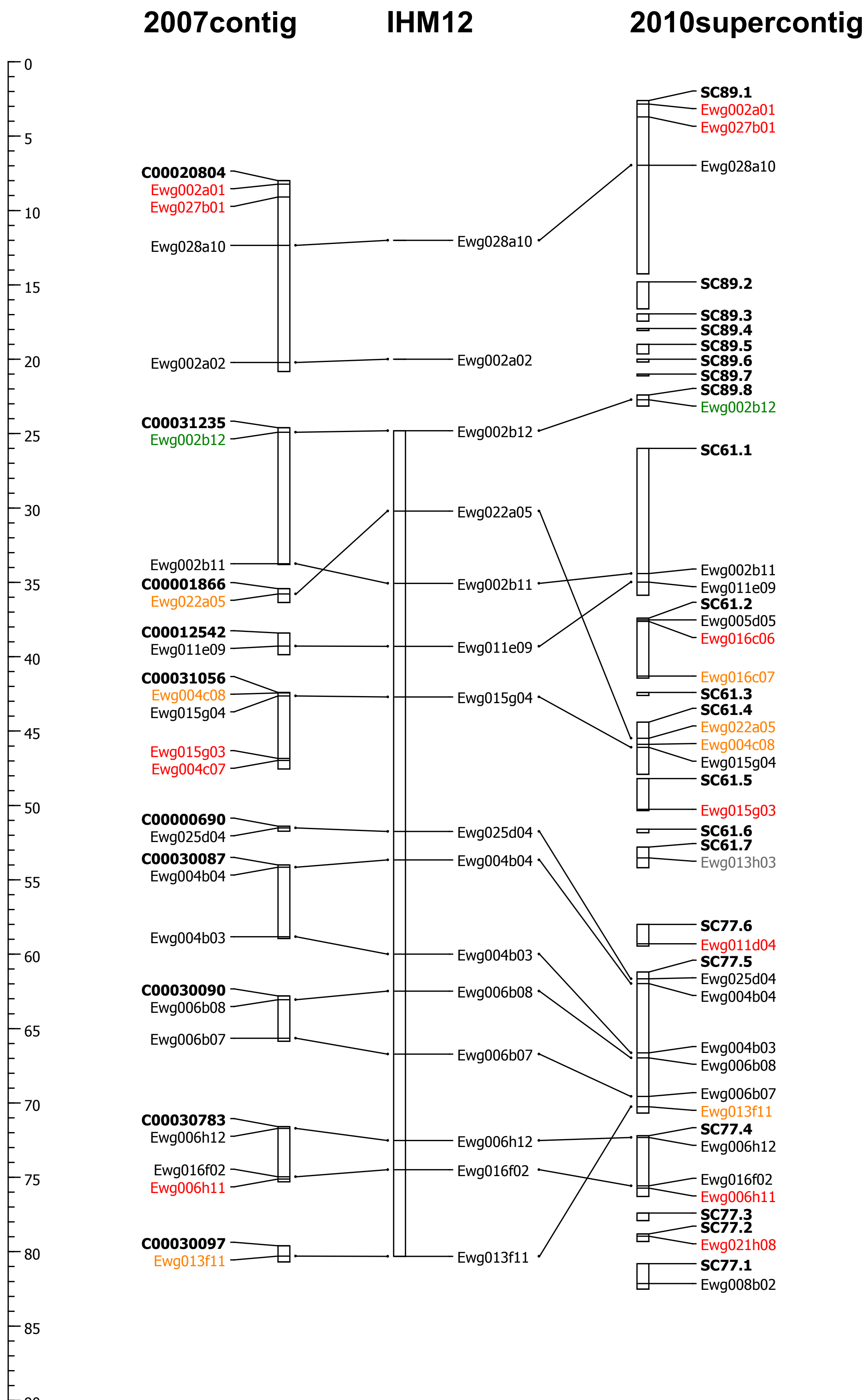

2007contig
IHM12
2010supercontig
0
5
10
15
20
25
30
35
40
45
50
55
60
65
70
75
80
85
C00020804
Ewg002a01
Ewg027b01
Ewg028a10
Ewg002a02
C00031235
Ewg002b12
Ewg002b11
C00001866
Ewg022a05
C00012542
Ewg011e09
C00031056
Ewg004c08
Ewg015g04
Ewg015g03
Ewg004c07
C00000690
Ewg025d04
C00030087
Ewg004b04
Ewg004b03
C00030090
Ewg006b08
Ewg006b07
C00030783
Ewg006h12
Ewg016f02
Ewg006h11
C00030097
Ewg013f11
Ewg028a10
Ewg002a02
Ewg002b12
Ewg022a05
Ewg002b11
Ewg011e09
Ewg015g04
Ewg025d04
Ewg004b04
Ewg004b03
Ewg006b08
Ewg006b07
Ewg006h12
Ewg016f02
Ewg013f11
SC89.1
Ewg002a01
Ewg027b01
Ewg028a10
SC89.2
SC89.3
SC89.4
SC89.5
SC89.6
SC89.7
SC89.8
Ewg002b12
SC61.1
Ewg002b11
Ewg011e09
SC61.2
Ewg005d05
Ewg016c06
Ewg016c07
SC61.3
SC61.4
Ewg022a05
Ewg004c08
Ewg015g04
SC61.5
Ewg015g03
SC61.6
SC61.7
Ewg013h03
SC77.6
Ewg011d04
SC77.5
Ewg025d04
Ewg004b04
Ewg004b03
Ewg006b08
Ewg006b07
Ewg013f11
SC77.4
Ewg006h12
Ewg016f02
Ewg006h11
SC77.3
SC77.2
Ewg021h08
SC77.1
Ewg008b02

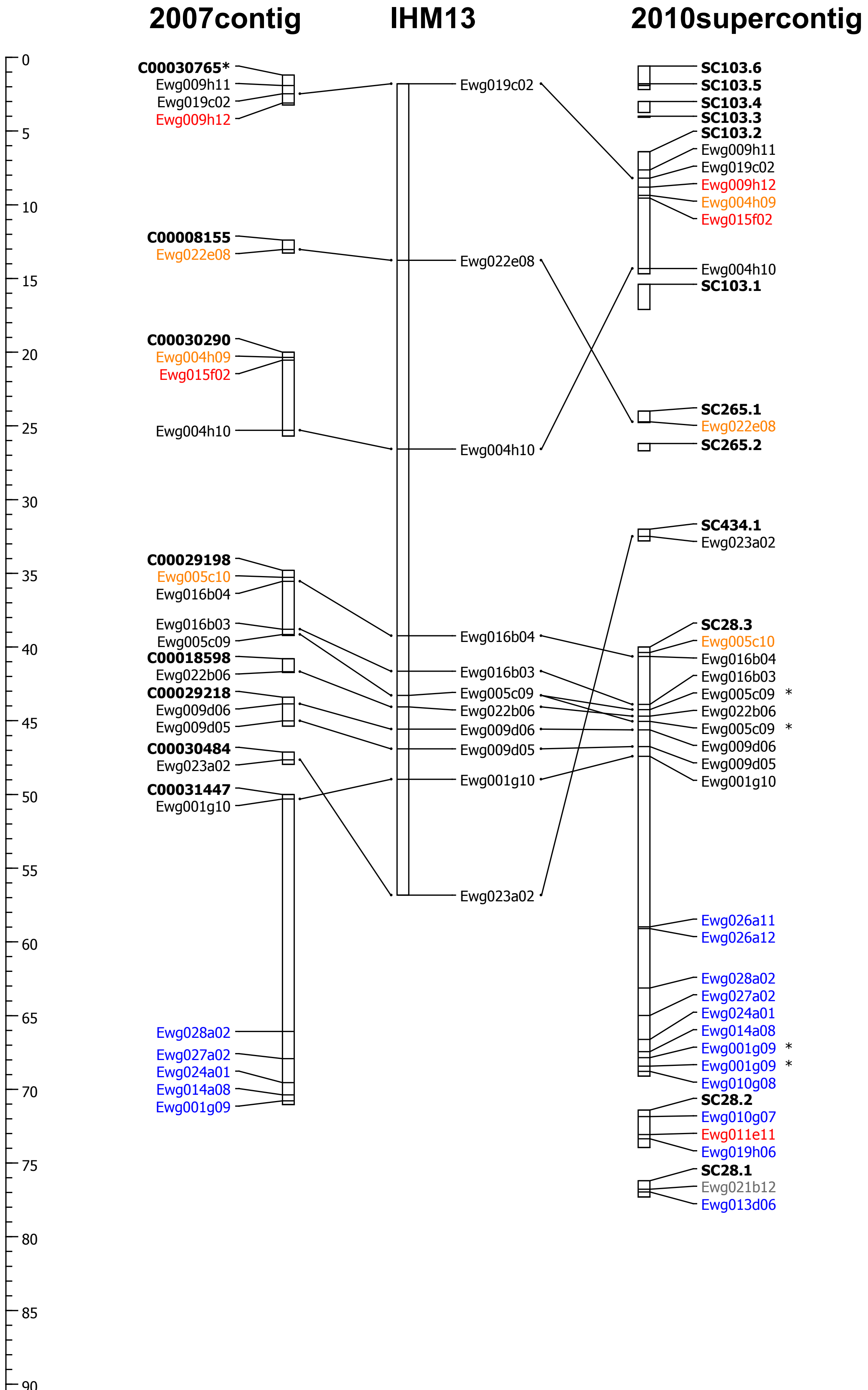

2007contig
IHM13
2010supercontig
0
5
10
15
20
25
30
35
40
45
50
55
60
65
70
75
80
85
90
C00030765*
Ewg009h11
Ewg019c02
Ewg009h12
C00008155
Ewg022e08
C00030290
Ewg004h09
Ewg015f02
Ewg004h10
C00029198
Ewg005c10
Ewg016b04
Ewg016b03
Ewg005c09
C00018598
Ewg022b06
C00029218
Ewg009d06
Ewg009d05
C00030484
Ewg023a02
C00031447
Ewg001g10
Ewg028a02
Ewg027a02
Ewg024a01
Ewg014a08
Ewg001g09
Ewg019c02
Ewg022e08
Ewg004h10
Ewg016b04
Ewg016b03
Ewg005c09
Ewg022b06
Ewg009d06
Ewg009d05
Ewg001g10
Ewg023a02
SC103.6
SC103.5
SC103.4
SC103.3
SC103.2
Ewg009h11
Ewg019c02
Ewg009h12
Ewg004h09
Ewg015f02
Ewg004h10
SC103.1
SC265.1
Ewg022e08
SC265.2
SC434.1
Ewg023a02
SC28.3
Ewg005c10
Ewg016b04
Ewg016b03
Ewg005c09 *
Ewg022b06
Ewg005c09 *
Ewg009d06
Ewg009d05
Ewg001g10
Ewg026a11
Ewg026a12
Ewg028a02
Ewg027a02
Ewg024a01
Ewg014a08
Ewg001g09 *
Ewg001g09 *
Ewg010g08
SC28.2
Ewg010g07
Ewg011e11
Ewg019h06
SC28.1
Ewg021b12
Ewg013d06

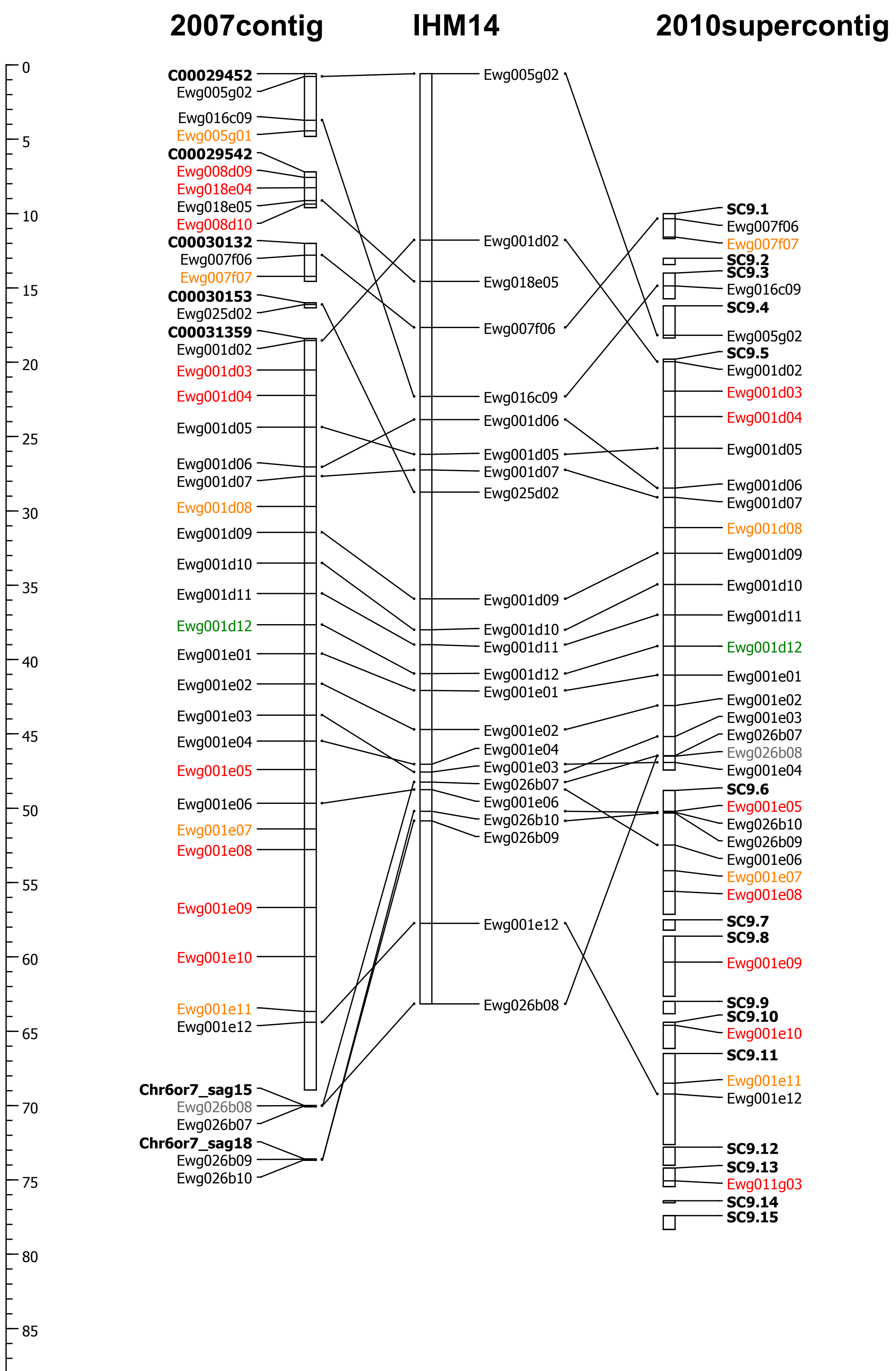

2007contig
IHM14
2010supercontig
0
5
10
15
20
25
30
35
40
45
50
55
60
65
70
75
80
85
C00029452
Ewg005g02
Ewg016c09
Ewg005g01
C00029542
Ewg008d09
Ewg018e04
Ewg018e05
Ewg008d10
C00030132
Ewg007f06
Ewg007f07
C00030153
Ewg025d02
C00031359
Ewg001d02
Ewg001d03
Ewg001d04
Ewg001d05
Ewg001d06
Ewg001d07
Ewg001d08
Ewg001d09
Ewg001d10
Ewg001d11
Ewg001d12
Ewg001e01
Ewg001e02
Ewg001e03
Ewg001e04
Ewg001e05
Ewg001e06
Ewg001e07
Ewg001e08
Ewg001e09
Ewg001e10
Ewg001e11
Ewg001e12
Chr6or7_sag15
Ewg026b08
Ewg026b07
Chr6or7_sag18
Ewg026b09
Ewg026b10
Ewg005g02
Ewg001d02
Ewg018e05
Ewg007f06
Ewg016c09
Ewg001d06
Ewg001d05
Ewg001d07
Ewg025d02
Ewg001d09
Ewg001d10
Ewg001d11
Ewg001d12
Ewg001e01
Ewg001e02
Ewg001e04
Ewg001e03
Ewg026b07
Ewg001e06
Ewg026b10
Ewg026b09
Ewg001e12
Ewg026b08
SC9.1
Ewg007f06
Ewg007f07
SC9.2
SC9.3
Ewg016c09
SC9.4
Ewg005g02
SC9.5
Ewg001d02
Ewg001d03
Ewg001d04
Ewg001d05
Ewg001d06
Ewg001d07
Ewg001d08
Ewg001d09
Ewg001d10
Ewg001d11
Ewg001d12
Ewg001e01
Ewg001e02
Ewg001e03
Ewg026b07
Ewg026b08
Ewg001e04
SC9.6
Ewg001e05
Ewg026b10
Ewg026b09
Ewg001e06
Ewg001e07
Ewg001e08
SC9.7
SC9.8
Ewg001e09
SC9.9
SC9.10
Ewg001e10
SC9.11
Ewg001e11
Ewg001e12
SC9.12
SC9.13
Ewg011g03
SC9.14
SC9.15

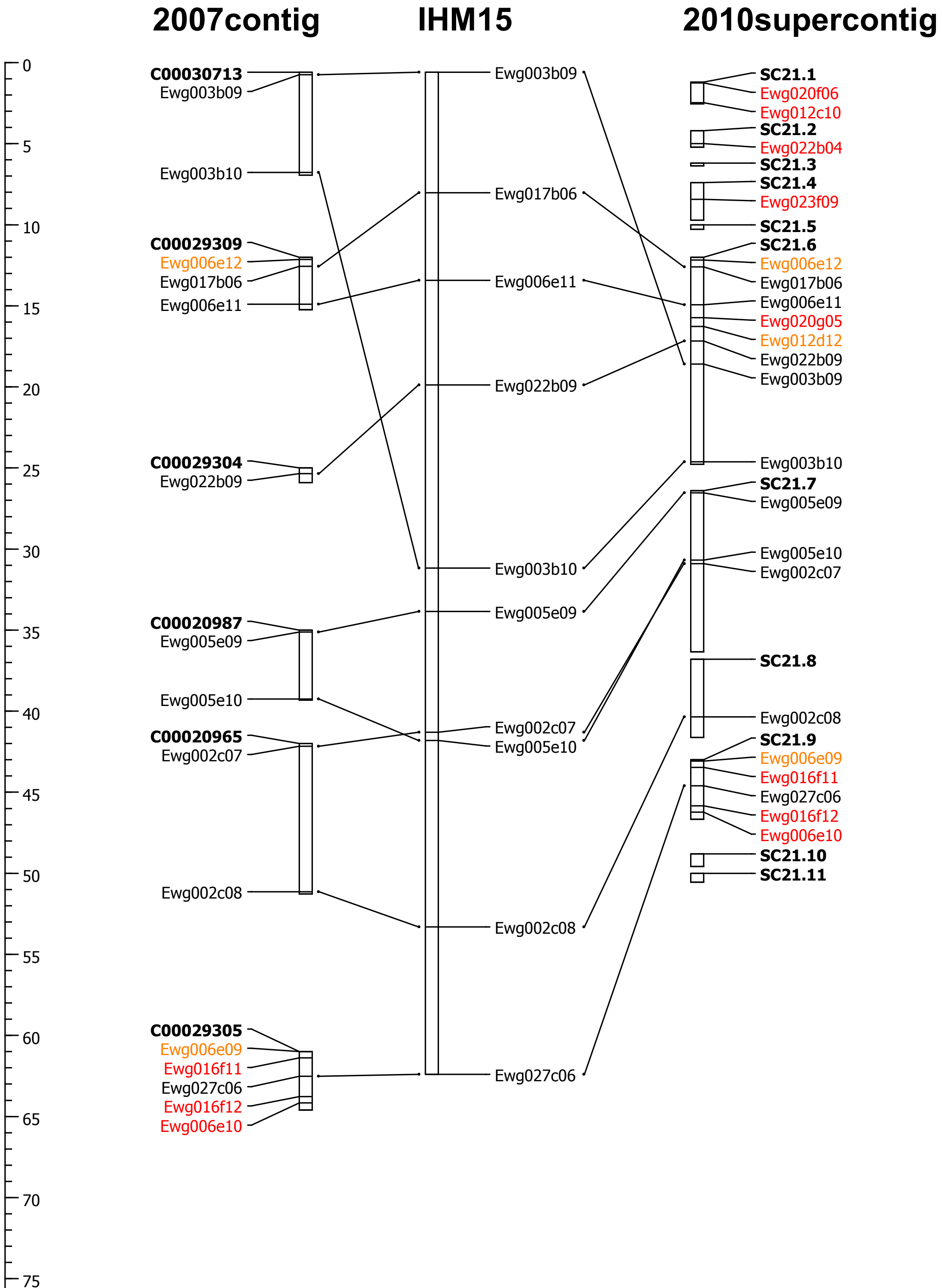
2007contig
IHM15
2010supercontig
0
5
10
15
20
25
30
35
40
45
50
55
60
65
70
75
C00030713
Ewg003b09
Ewg003b10
C00029309
Ewg006e12
Ewg017b06
Ewg006e11
C00029304
Ewg022b09
C00020987
Ewg005e09
Ewg005e10
C00020965
Ewg002c07
Ewg002c08
C00029305
Ewg006e09
Ewg016f11
Ewg027c06
Ewg016f12
Ewg006e10
Ewg003b09
Ewg017b06
Ewg006e11
Ewg022b09
Ewg003b10
Ewg005e09
Ewg002c07
Ewg005e10
Ewg002c08
Ewg027c06
SC21.1
Ewg020f06
Ewg012c10
SC21.2
Ewg022b04
SC21.3
SC21.4
Ewg023f09
SC21.5
SC21.6
Ewg006e12
Ewg017b06
Ewg006e11
Ewg020g05
Ewg012d12
Ewg022b09
Ewg003b09
Ewg003b10
SC21.7
Ewg005e09
Ewg005e10
Ewg002c07
SC21.8
Ewg002c08
SC21.9
Ewg006e09
Ewg016f11
Ewg027c06
Ewg016f12
Ewg006e10
SC21.10
SC21.11

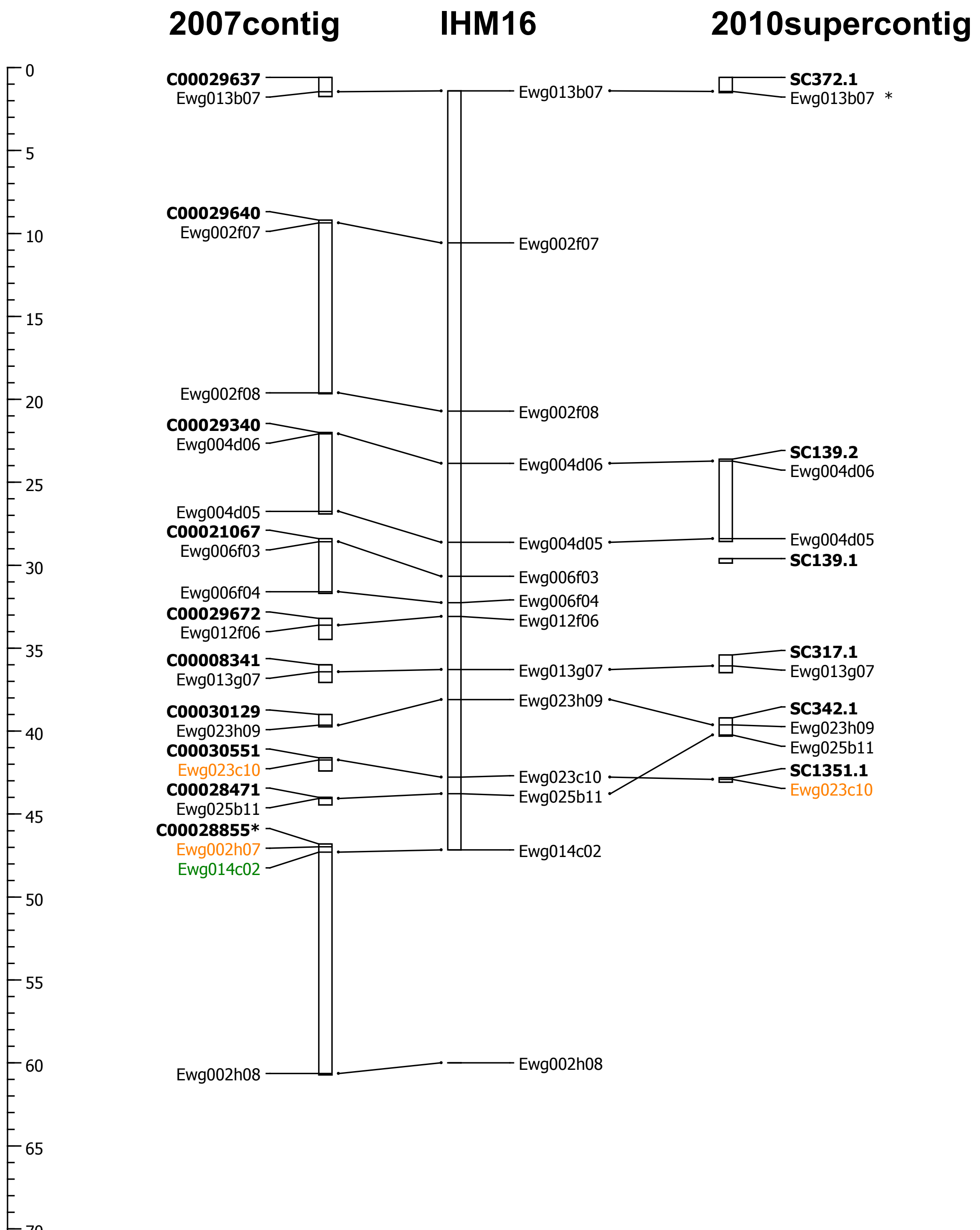
2007contig
IHM16
2010supercontig
0
5
10
15
20
25
30
35
40
45
50
55
60
65
70
C00029637
Ewg013b07
C00029640
Ewg002f07
Ewg002f08
C00029340
Ewg004d06
Ewg004d05
C00021067
Ewg006f03
Ewg006f04
C00029672
Ewg012f06
C00008341
Ewg013g07
C00030129
Ewg023h09
C00030551
Ewg023c10
C00028471
Ewg025b11
C00028855*
Ewg002h07
Ewg014c02
Ewg002h08
Ewg013b07
Ewg002f07
Ewg002f08
Ewg004d06
Ewg004d05
Ewg006f03
Ewg006f04
Ewg012f06
Ewg013g07
Ewg023h09
Ewg023c10
Ewg025b11
Ewg014c02
Ewg002h08
SC372.1
Ewg013b07 *
SC139.2
Ewg004d06
Ewg004d05
SC139.1
SC317.1
Ewg013g07
SC342.1
Ewg023h09
Ewg025b11
SC1351.1
Ewg023c10

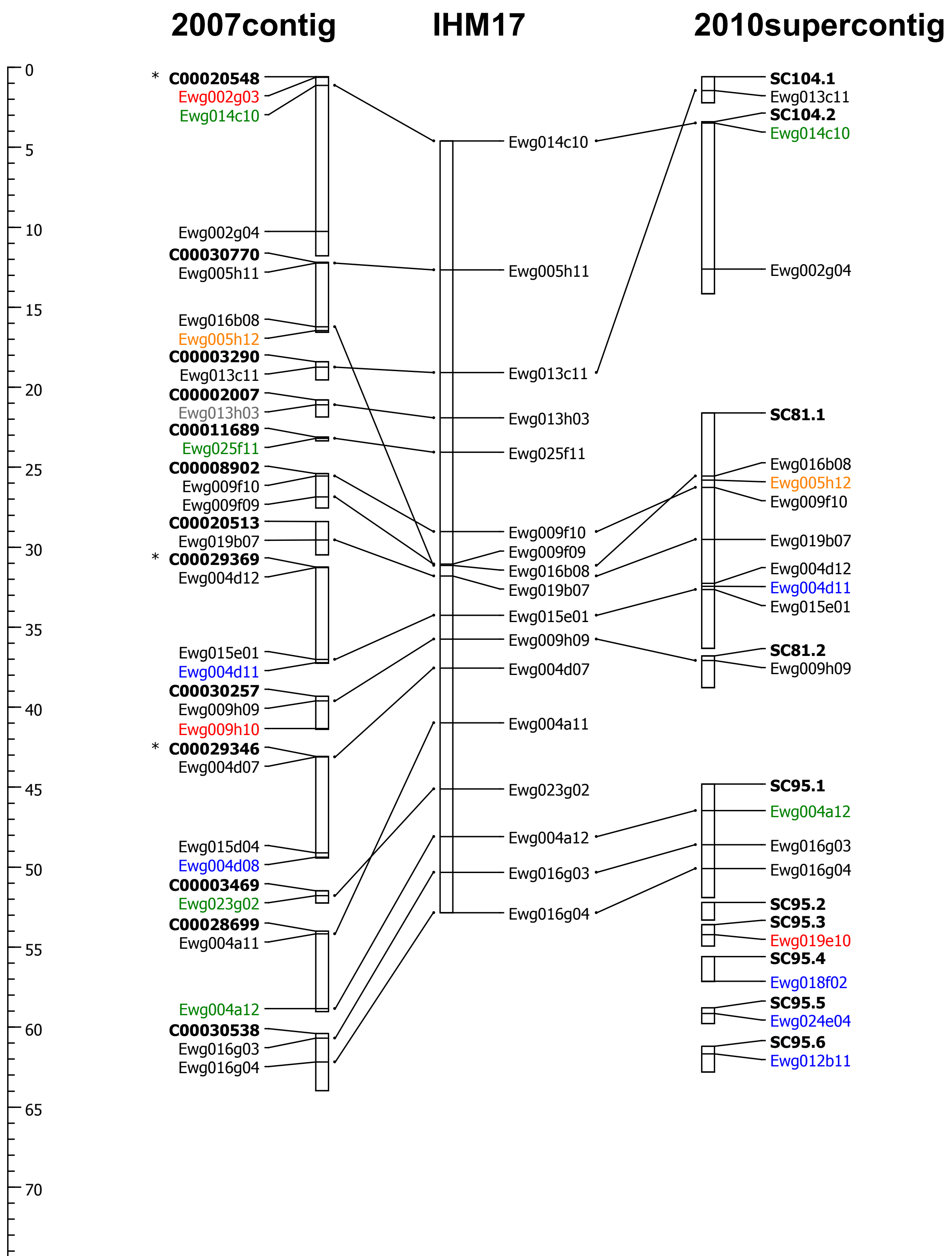
2007contig
IHM17
2010supercontig
0
5
10
15
20
25
30
35
40
45
50
55
60
65
70
* C00020548
Ewg002g03
Ewg014c10
Ewg002g04
C00030770
Ewg005h11
Ewg016b08
Ewg005h12
C00003290
Ewg013c11
C00002007
Ewg013h03
C00011689
Ewg025f11
C00008902
Ewg009f10
Ewg009f09
C00020513
Ewg019b07
* C00029369
Ewg004d12
Ewg015e01
Ewg004d11
C00030257
Ewg009h09
Ewg009h10
* C00029346
Ewg004d07
Ewg015d04
Ewg004d08
C00003469
Ewg023g02
C00028699
Ewg004a11
Ewg004a12
C00030538
Ewg016g03
Ewg016g04
Ewg014c10
Ewg005h11
Ewg013c11
Ewg013h03
Ewg025f11
Ewg009f10
Ewg009f09
Ewg016b08
Ewg019b07
Ewg015e01
Ewg009h09
Ewg004d07
Ewg004a11
Ewg023g02
Ewg004a12
Ewg016g03
Ewg016g04
SC104.1
Ewg013c11
SC104.2
Ewg014c10
Ewg002g04
SC81.1
Ewg016b08
Ewg005h12
Ewg009f10
Ewg019b07
Ewg004d12
Ewg004d11
Ewg015e01
SC81.2
Ewg009h09
SC95.1
Ewg004a12
Ewg016g03
Ewg016g04
SC95.2
SC95.3
Ewg019e10
SC95.4
Ewg018f02
SC95.5
Ewg024e04
SC95.6
Ewg012b11

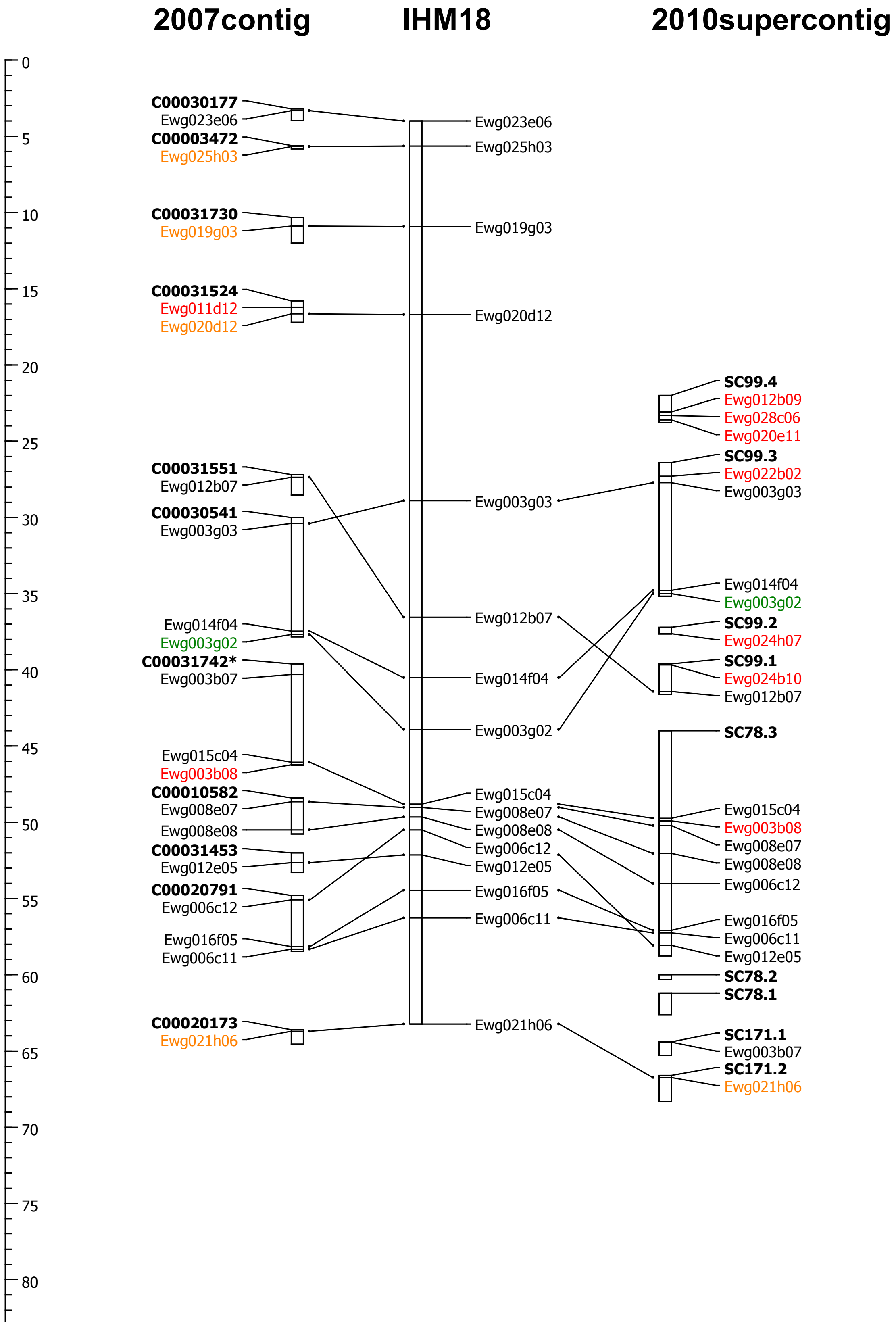

2007contig
IHM18
2010supercontig
C00030177
Ewg023e06
C00003472
Ewg025h03
C00031730
Ewg019g03
C00031524
Ewg011d12
Ewg020d12
C00031551
Ewg012b07
C00030541
Ewg003g03
Ewg014f04
Ewg003g02
C00031742*
Ewg003b07
Ewg015c04
Ewg003b08
C00010582
Ewg008e07
Ewg008e08
C00031453
Ewg012e05
C00020791
Ewg006c12
Ewg016f05
Ewg006c11
C00020173
Ewg021h06
Ewg023e06
Ewg025h03
Ewg019g03
Ewg020d12
Ewg003g03
Ewg012b07
Ewg014f04
Ewg003g02
Ewg015c04
Ewg008e07
Ewg008e08
Ewg006c12
Ewg012e05
Ewg016f05
Ewg006c11
Ewg021h06
SC99.4
Ewg012b09
Ewg028c06
Ewg020e11
SC99.3
Ewg022b02
Ewg003g03
Ewg014f04
Ewg003g02
SC99.2
Ewg024h07
SC99.1
Ewg024b10
Ewg012b07
SC78.3
Ewg015c04
Ewg003b08
Ewg008e07
Ewg008e08
Ewg006c12
Ewg016f05
Ewg006c11
Ewg012e05
SC78.2
SC78.1
SC171.1
Ewg003b07
SC171.2
Ewg021h06
0
5
10
15
20
25
30
35
40
45
50
55
60
65
70
75
80

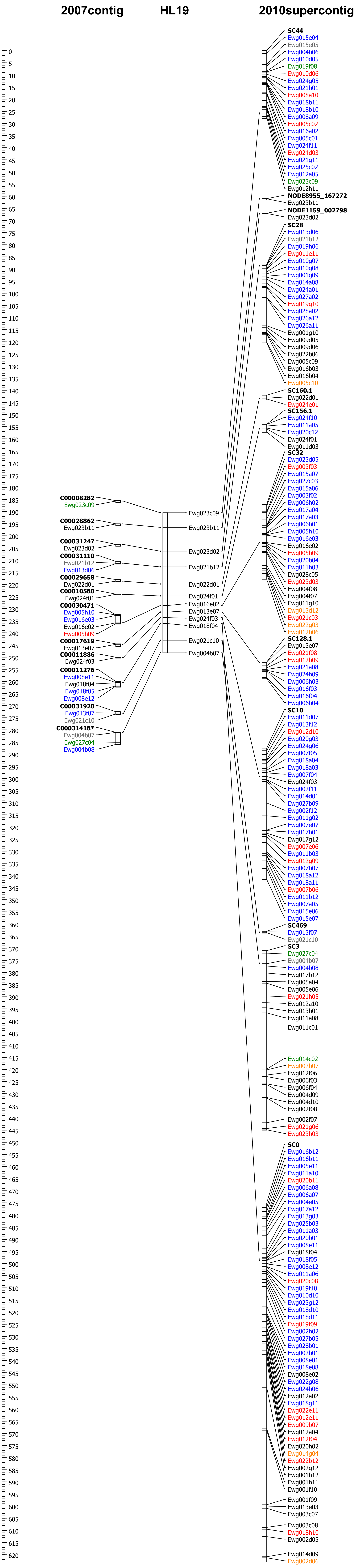

2007contig
HL19
2010supercontig
C00008282
Ewg023c09
C00028862
Ewg023b11
C00031247
Ewg023d02
C00031110
Ewg021b12
Ewg013d06
C00029658
Ewg022d01
C00010580
Ewg024f01
C00030471
Ewg005h10
Ewg016e03
Ewg016e02
Ewg005h09
C00017619
Ewg013e07
C00011886
Ewg024f03
C00011276
Ewg008e11
Ewg018f04
Ewg018f05
Ewg008e12
C00031920
Ewg013f07
Ewg021c10
C00031418*
Ewg004b07
Ewg027c04
Ewg004b08
Ewg023c09
Ewg023b11
Ewg023d02
Ewg021b12
Ewg022d01
Ewg024f01
Ewg016e02
Ewg013e07
Ewg024f03
Ewg018f04
Ewg021c10
Ewg004b07
SC44
Ewg015e04
Ewg015e05
Ewg004b06
Ewg010d05
Ewg019f08
Ewg010d06
Ewg024g05
Ewg021h01
Ewg008a10
Ewg018b11
Ewg018b10
Ewg008a09
Ewg005c02
Ewg016a02
Ewg005c01
Ewg024f11
Ewg024d03
Ewg021g11
Ewg025c02
Ewg012a05
Ewg023c09
Ewg012h11
NODE8955_167272
Ewg023b11
NODE1159_002798
Ewg023d02
SC28
Ewg013d06
Ewg021b12
Ewg019h06
Ewg011e11
Ewg010g07
Ewg010g08
Ewg001g09
Ewg014a08
Ewg024a01
Ewg027a02
Ewg019g10
Ewg028a02
Ewg026a12
Ewg026a11
Ewg001g10
Ewg009d05
Ewg009d06
Ewg022b06
Ewg005c09
Ewg016b03
Ewg016b04
Ewg005c10
SC160.1
Ewg022d01
Ewg024e01
SC156.1
Ewg024f10
Ewg011a05
Ewg020c12
Ewg024f01
Ewg011d03
SC32
Ewg023d05
Ewg003f03
Ewg015a07
Ewg027c03
Ewg015a06
Ewg003f02
Ewg006h02
Ewg017a04
Ewg017a03
Ewg006h01
Ewg005h10
Ewg016e03
Ewg016e02
Ewg005h09
Ewg020b04
Ewg011h03
Ewg028c05
Ewg023d03
Ewg004f08
Ewg004f07
Ewg011g10
Ewg013d12
Ewg021c03
Ewg022g03
Ewg012b06
SC128.1
Ewg013e07
Ewg021f08
Ewg012h09
Ewg021a08
Ewg024h09
Ewg006h03
Ewg016f03
Ewg016f04
Ewg006h04
SC10
Ewg011d07
Ewg013f12
Ewg012d10
Ewg020g03
Ewg024g06
Ewg007f05
Ewg018a04
Ewg018a03
Ewg007f04
Ewg024f03
Ewg002f11
Ewg014d01
Ewg027b09
Ewg002f12
Ewg011g02
Ewg007e07
Ewg017h01
Ewg017g12
Ewg007e06
Ewg011b03
Ewg012g09
Ewg007b07
Ewg018a12
Ewg018a11
Ewg007b06
Ewg011b12
Ewg007a05
Ewg015e06
Ewg015e07
SC469
Ewg013f07
Ewg021c10
SC3
Ewg027c04
Ewg004b07
Ewg004b08
Ewg017b12
Ewg005a04
Ewg005e06
Ewg021h05
Ewg012a10
Ewg013h01
Ewg011a08
Ewg011c01
Ewg014c02
Ewg002h07
Ewg012f06
Ewg006f03
Ewg006f04
Ewg004d09
Ewg004d10
Ewg002f08
Ewg002f07
Ewg021g06
Ewg023h03
SC0
Ewg016b12
Ewg016b11
Ewg005e11
Ewg011a10
Ewg020b11
Ewg006a08
Ewg006a07
Ewg004e05
Ewg017a12
Ewg013g03
Ewg025b03
Ewg011a03
Ewg020b01
Ewg008e11
Ewg018f04
Ewg018f05
Ewg008e12
Ewg011a06
Ewg020c08
Ewg019f10
Ewg010d10
Ewg023g12
Ewg018d10
Ewg018d11
Ewg019f09
Ewg002h02
Ewg027b05
Ewg028b01
Ewg002h01
Ewg008e01
Ewg018e08
Ewg008e02
Ewg022g08
Ewg024h06
Ewg012a02
Ewg018g11
Ewg022e11
Ewg012e11
Ewg009b07
Ewg012a04
Ewg012f04
Ewg020h02
Ewg014g04
Ewg022b12
Ewg002g12
Ewg001h12
Ewg001h11
Ewg001f10
Ewg001f09
Ewg013e03
Ewg003c07
Ewg003c08
Ewg018h10
Ewg002d05
Ewg014d09
Ewg002d06

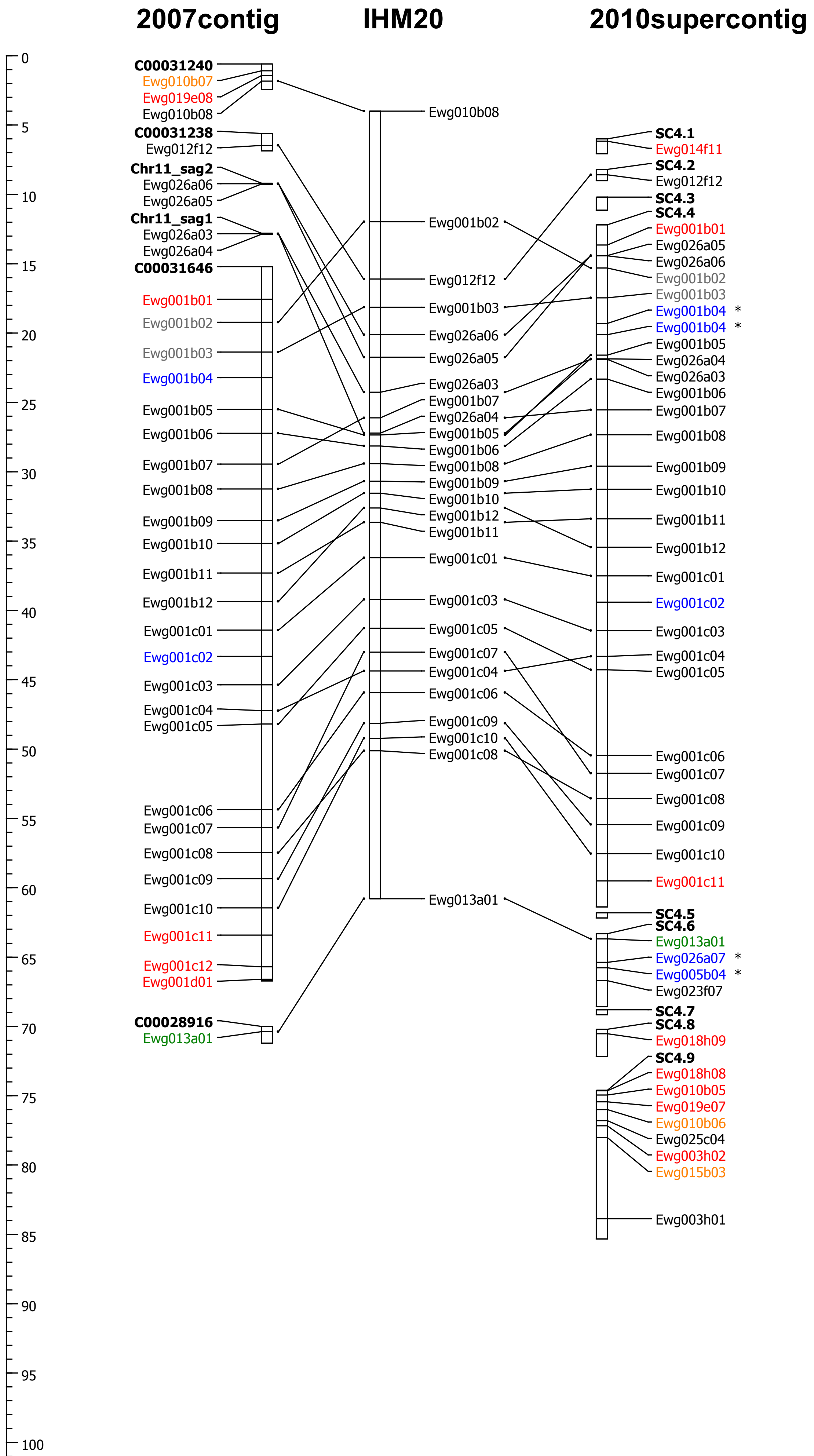
2007contig
IHM20
2010supercontig
0
5
10
15
20
25
30
35
40
45
50
55
60
65
70
75
80
85
90
95
100
C00031240
Ewg010b07
Ewg019e08
Ewg010b08
C00031238
Ewg012f12
Chr11_sag2
Ewg026a06
Ewg026a05
Chr11_sag1
Ewg026a03
Ewg026a04
C00031646
Ewg001b01
Ewg001b02
Ewg001b03
Ewg001b04
Ewg001b05
Ewg001b06
Ewg001b07
Ewg001b08
Ewg001b09
Ewg001b10
Ewg001b11
Ewg001b12
Ewg001c01
Ewg001c02
Ewg001c03
Ewg001c04
Ewg001c05
Ewg001c06
Ewg001c07
Ewg001c08
Ewg001c09
Ewg001c10
Ewg001c11
Ewg001c12
Ewg001d01
C00028916
Ewg013a01
Ewg010b08
Ewg001b02
Ewg012f12
Ewg001b03
Ewg026a06
Ewg026a05
Ewg026a03
Ewg001b07
Ewg026a04
Ewg001b05
Ewg001b06
Ewg001b08
Ewg001b09
Ewg001b10
Ewg001b12
Ewg001b11
Ewg001c01
Ewg001c03
Ewg001c05
Ewg001c07
Ewg001c04
Ewg001c06
Ewg001c09
Ewg001c10
Ewg001c08
Ewg013a01
SC4.1
Ewg014f11
SC4.2
Ewg012f12
SC4.3
SC4.4
Ewg001b01
Ewg026a05
Ewg026a06
Ewg001b02
Ewg001b03
Ewg001b04 *
Ewg001b04 *
Ewg001b05
Ewg026a04
Ewg026a03
Ewg001b06
Ewg001b07
Ewg001b08
Ewg001b09
Ewg001b10
Ewg001b11
Ewg001b12
Ewg001c01
Ewg001c02
Ewg001c03
Ewg001c04
Ewg001c05
Ewg001c06
Ewg001c07
Ewg001c08
Ewg001c09
Ewg001c10
Ewg001c11
SC4.5
SC4.6
Ewg013a01
Ewg026a07 *
Ewg005b04 *
Ewg023f07
SC4.7
SC4.8
Ewg018h09
SC4.9
Ewg018h08
Ewg010b05
Ewg019e07
Ewg010b06
Ewg025c04
Ewg003h02
Ewg015b03
Ewg003h01

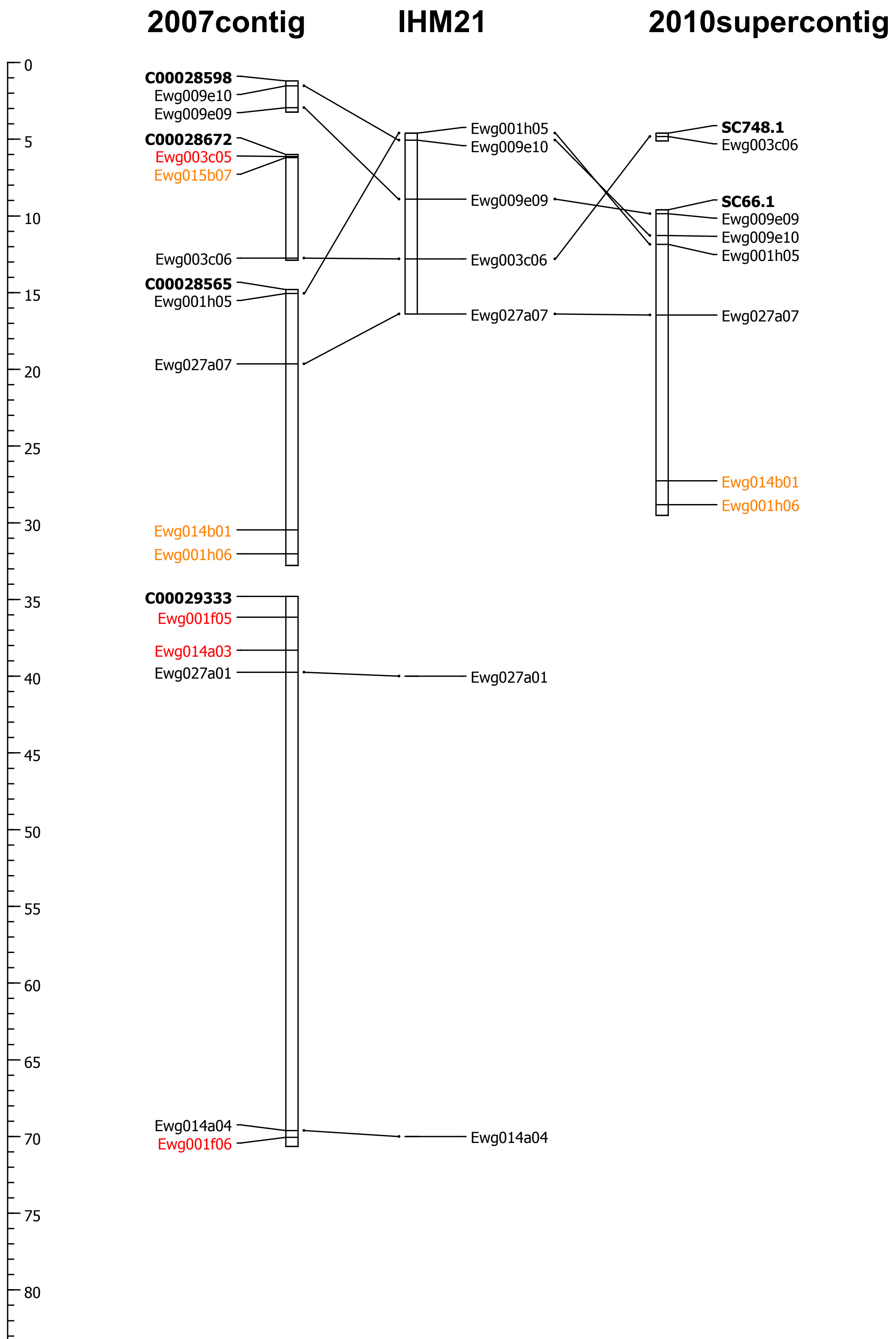

2007contig
IHM21
2010supercontig
0
5
10
15
20
25
30
35
40
45
50
55
60
65
70
75
80
C00028598
Ewg009e10
Ewg009e09
C00028672
Ewg003c05
Ewg015b07
Ewg003c06
C00028565
Ewg001h05
Ewg027a07
Ewg014b01
Ewg001h06
C00029333
Ewg001f05
Ewg014a03
Ewg027a01
Ewg014a04
Ewg001f06
Ewg001h05
Ewg009e10
Ewg009e09
Ewg003c06
Ewg027a07
Ewg027a01
Ewg014a04
SC748.1
Ewg003c06
SC66.1
Ewg009e09
Ewg009e10
Ewg001h05
Ewg027a07
Ewg014b01
Ewg001h06

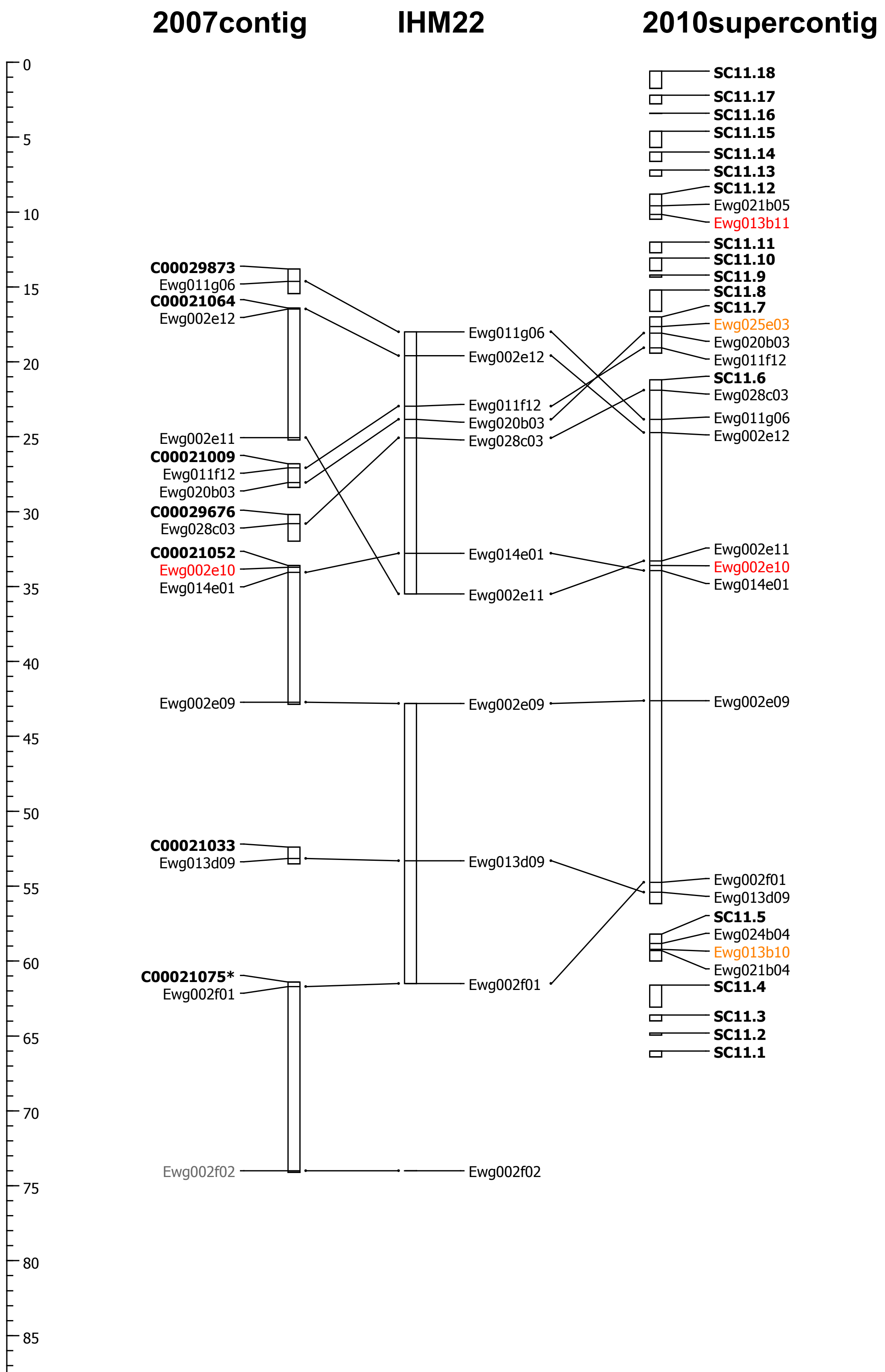
2007contig
IHM22
2010supercontig
0
5
10
15
20
25
30
35
40
45
50
55
60
65
70
75
80
85
C00029873
Ewg011g06
C00021064
Ewg002e12
Ewg002e11
C00021009
Ewg011f12
Ewg020b03
C00029676
Ewg028c03
C00021052
Ewg002e10
Ewg014e01
Ewg002e09
C00021033
Ewg013d09
C00021075*
Ewg002f01
Ewg002f02
Ewg011g06
Ewg002e12
Ewg011f12
Ewg020b03
Ewg028c03
Ewg014e01
Ewg002e11
Ewg002e09
Ewg013d09
Ewg002f01
Ewg002f02
SC11.18
SC11.17
SC11.16
SC11.15
SC11.14
SC11.13
SC11.12
Ewg021b05
Ewg013b11
SC11.11
SC11.10
SC11.9
SC11.8
SC11.7
Ewg025e03
Ewg020b03
Ewg011f12
SC11.6
Ewg028c03
Ewg011g06
Ewg002e12
Ewg002e11
Ewg002e10
Ewg014e01
Ewg002e09
Ewg002f01
Ewg013d09
SC11.5
Ewg024b04
Ewg013b10
Ewg021b04
SC11.4
SC11.3
SC11.2
SC11.1

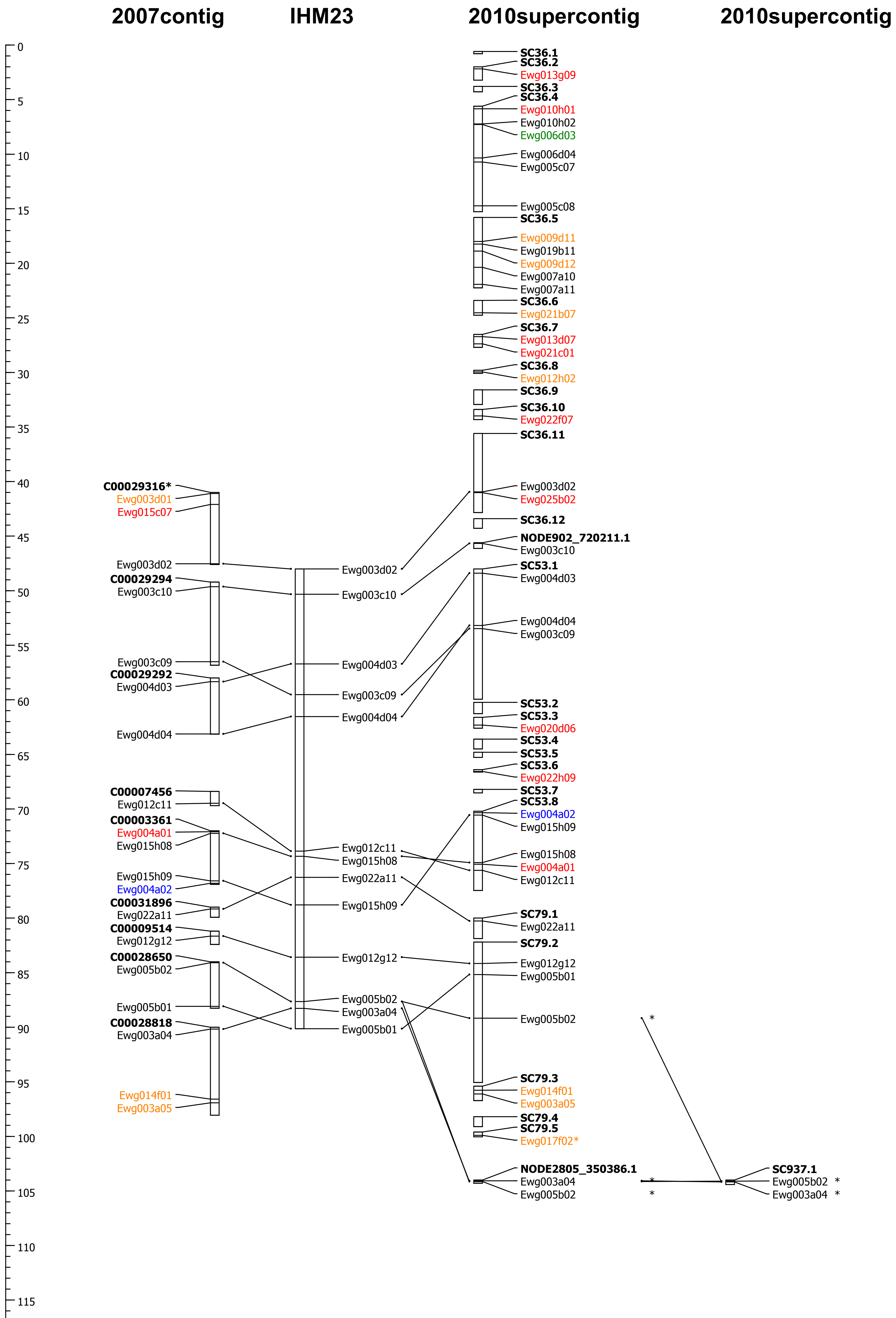
2007contig
IHM23
2010supercontig
2010supercontig
SC36.1
SC36.2
Ewg013g09
SC36.3
SC36.4
Ewg010h01
Ewg010h02
Ewg006d03
Ewg006d04
Ewg005c07
Ewg005c08
SC36.5
Ewg009d11
Ewg019b11
Ewg009d12
Ewg007a10
Ewg007a11
SC36.6
Ewg021b07
SC36.7
Ewg013d07
Ewg021c01
SC36.8
Ewg012h02
SC36.9
SC36.10
Ewg022f07
SC36.11
Ewg003d02
Ewg025b02
SC36.12
NODE902_720211.1
Ewg003c10
SC53.1
Ewg004d03
Ewg004d04
Ewg003c09
SC53.2
SC53.3
Ewg020d06
SC53.4
SC53.5
SC53.6
Ewg022h09
SC53.7
SC53.8
Ewg004a02
Ewg015h09
Ewg015h08
Ewg004a01
Ewg012c11
SC79.1
Ewg022a11
SC79.2
Ewg012g12
Ewg005b01
Ewg005b02
SC79.3
Ewg014f01
Ewg003a05
SC79.4
SC79.5
Ewg017f02*
NODE2805_350386.1
Ewg003a04
Ewg005b02
SC937.1
Ewg005b02 *
Ewg003a04 *
C00029316*
Ewg003d01
Ewg015c07
Ewg003d02
C00029294
Ewg003c10
Ewg003c09
C00029292
Ewg004d03
Ewg004d04
C00007456
Ewg012c11
C00003361
Ewg004a01
Ewg015h08
Ewg015h09
Ewg004a02
C00031896
Ewg022a11
C00009514
Ewg012g12
C00028650
Ewg005b02
Ewg005b01
C00028818
Ewg003a04
Ewg014f01
Ewg003a05
Ewg003d02
Ewg003c10
Ewg004d03
Ewg003c09
Ewg004d04
Ewg012c11
Ewg015h08
Ewg022a11
Ewg015h09
Ewg012g12
Ewg005b02
Ewg003a04
Ewg005b01
0
5
10
15
20
25
30
35
40
45
50
55
60
65
70
75
80
85
90
95
100
105
110
115

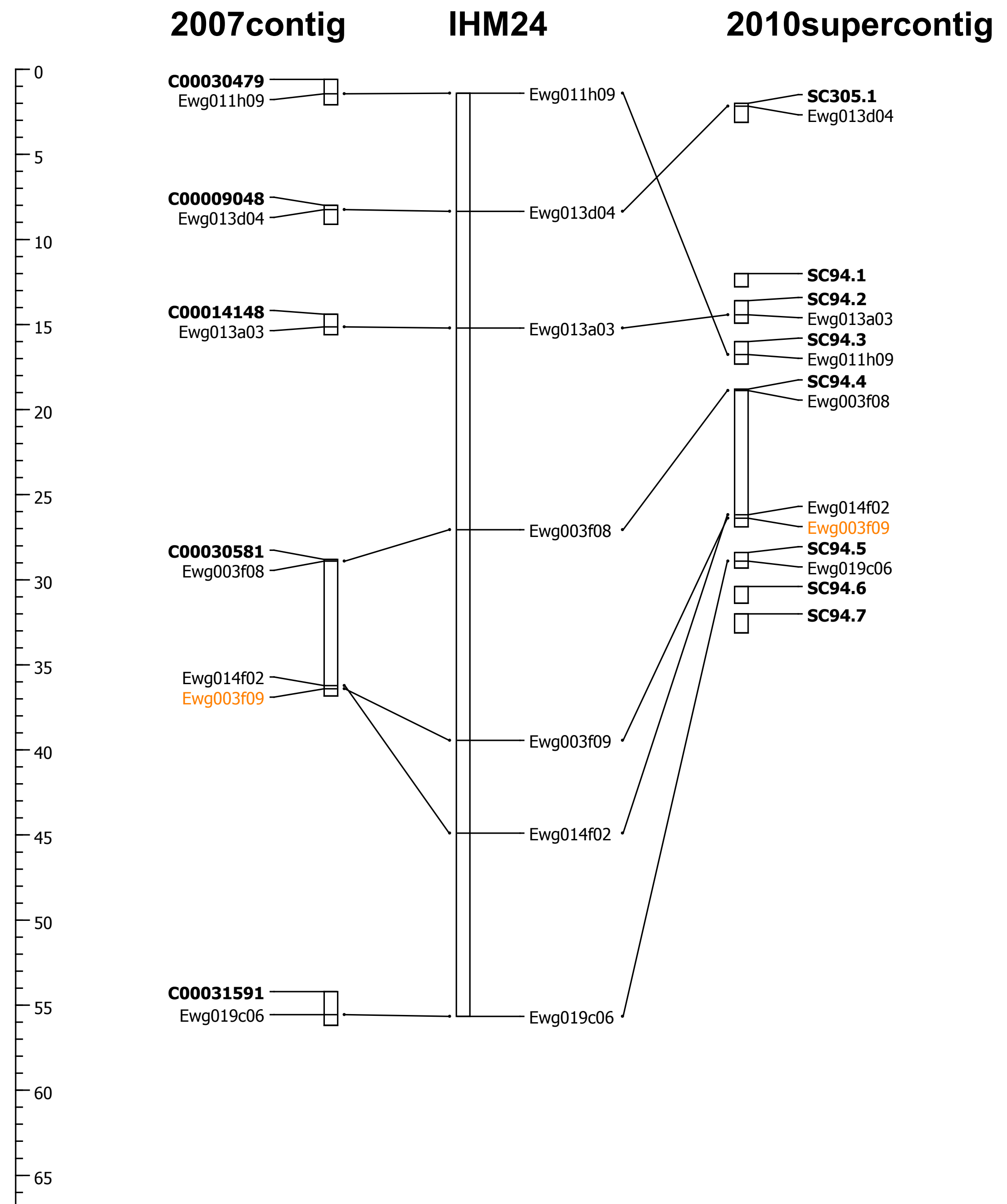

2007contig
IHM24
2010supercontig
0
5
10
15
20
25
30
35
40
45
50
55
60
65
C00030479
Ewg011h09
C00009048
Ewg013d04
C00014148
Ewg013a03
C00030581
Ewg003f08
Ewg014f02
Ewg003f09
C00031591
Ewg019c06
Ewg011h09
Ewg013d04
Ewg013a03
Ewg003f08
Ewg003f09
Ewg014f02
Ewg019c06
SC305.1
Ewg013d04
SC94.1
SC94.2
Ewg013a03
SC94.3
Ewg011h09
SC94.4
Ewg003f08
Ewg014f02
Ewg003f09
SC94.5
Ewg019c06
SC94.6
SC94.7

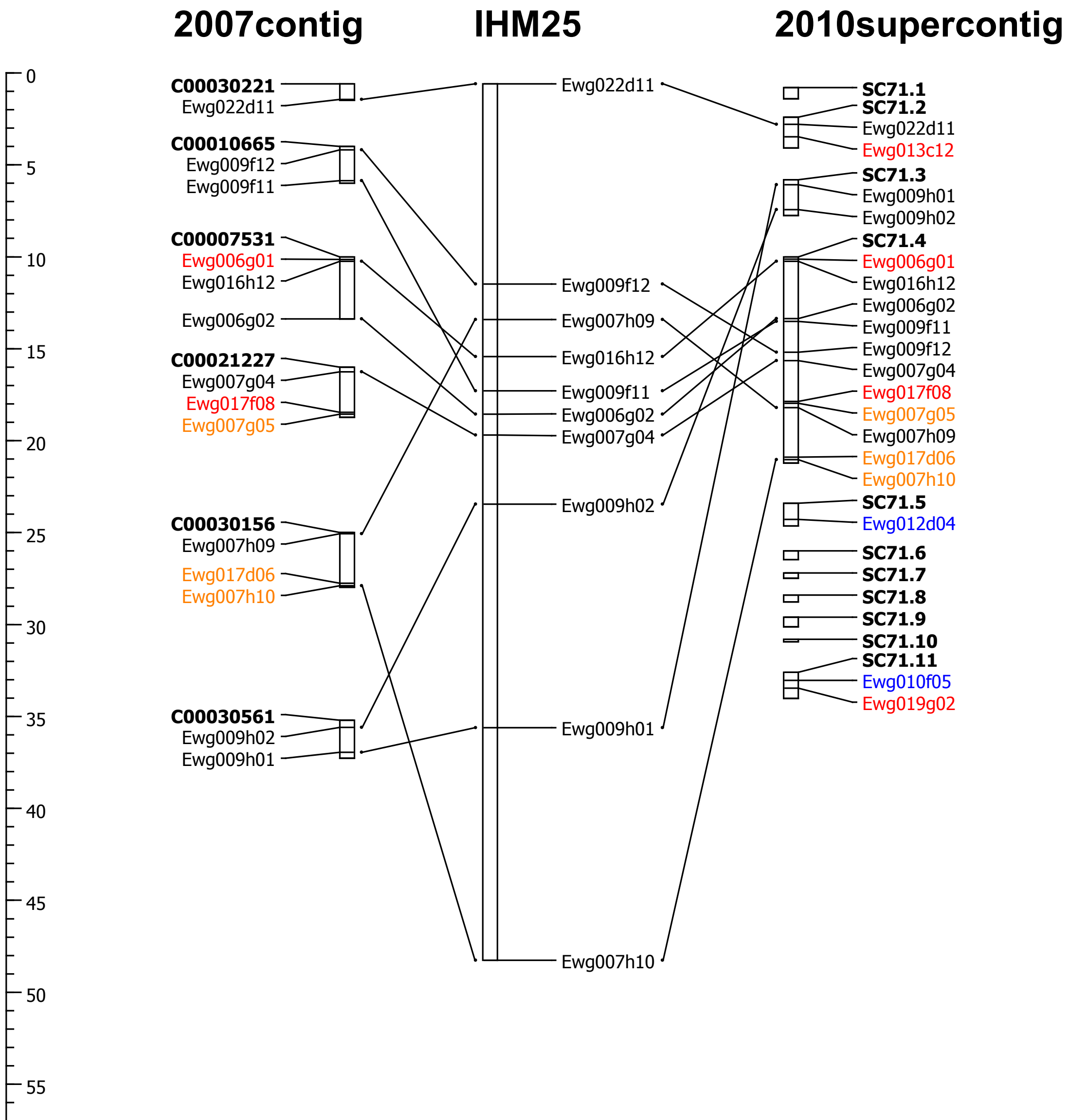

2007contig
IHM25
2010supercontig
0
5
10
15
20
25
30
35
40
45
50
55
C00030221
Ewg022d11
C00010665
Ewg009f12
Ewg009f11
C00007531
Ewg006g01
Ewg016h12
Ewg006g02
C00021227
Ewg007g04
Ewg017f08
Ewg007g05
C00030156
Ewg007h09
Ewg017d06
Ewg007h10
C00030561
Ewg009h02
Ewg009h01
Ewg022d11
Ewg009f12
Ewg007h09
Ewg016h12
Ewg009f11
Ewg006g02
Ewg007g04
Ewg009h02
Ewg009h01
Ewg007h10
SC71.1
SC71.2
Ewg022d11
Ewg013c12
SC71.3
Ewg009h01
Ewg009h02
SC71.4
Ewg006g01
Ewg016h12
Ewg006g02
Ewg009f11
Ewg009f12
Ewg007g04
Ewg017f08
Ewg007g05
Ewg007h09
Ewg017d06
Ewg007h10
SC71.5
Ewg012d04
SC71.6
SC71.7
SC71.8
SC71.9
SC71.10
SC71.11
Ewg010f05
Ewg019g02

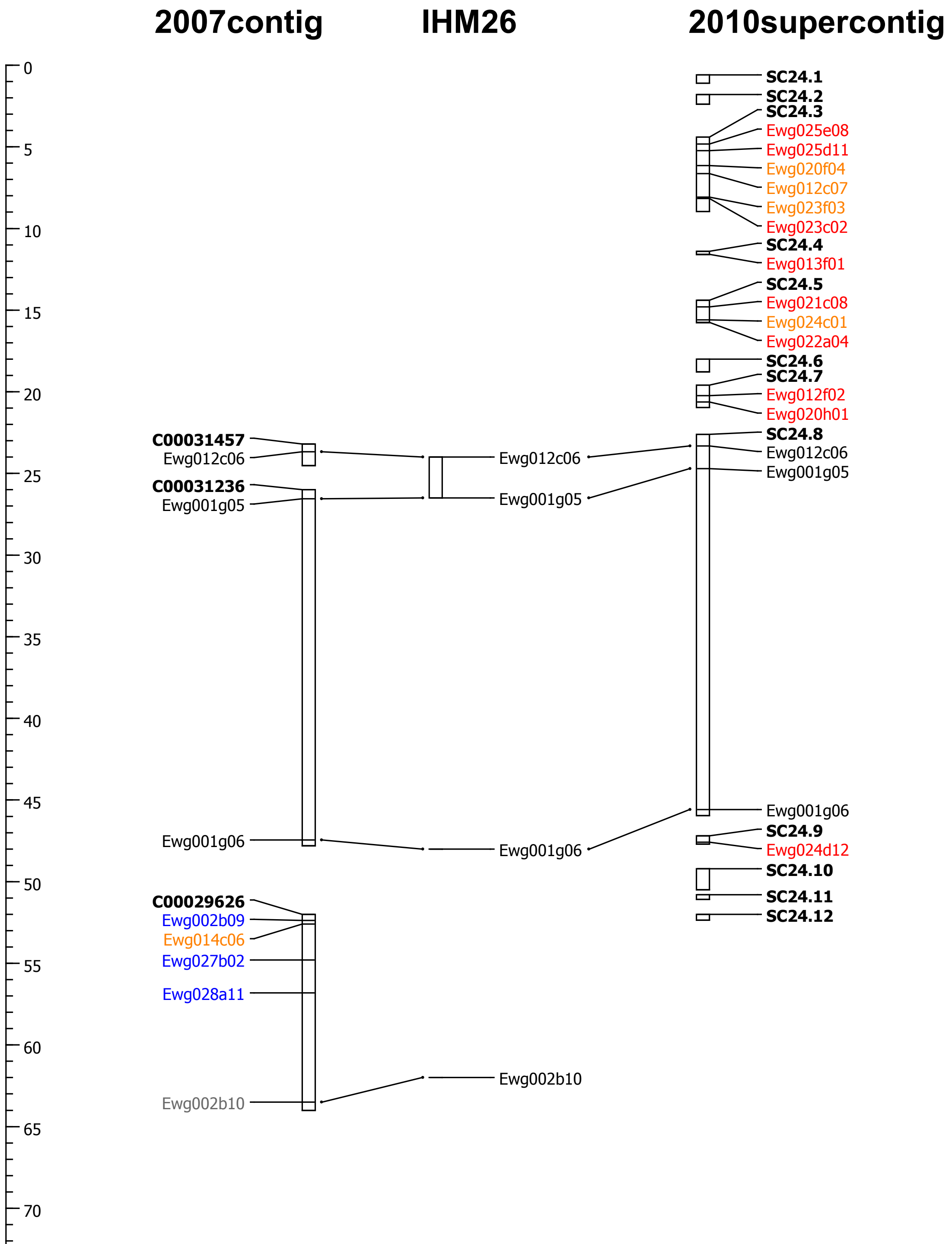

2007contig
IHM26
2010supercontig
0
5
10
15
20
25
30
35
40
45
50
55
60
65
70
C00031457
Ewg012c06
C00031236
Ewg001g05
Ewg001g06
C00029626
Ewg002b09
Ewg014c06
Ewg027b02
Ewg028a11
Ewg002b10
Ewg012c06
Ewg001g05
Ewg001g06
Ewg002b10
SC24.1
SC24.2
SC24.3
Ewg025e08
Ewg025d11
Ewg020f04
Ewg012c07
Ewg023f03
Ewg023c02
SC24.4
Ewg013f01
SC24.5
Ewg021c08
Ewg024c01
Ewg022a04
SC24.6
SC24.7
Ewg012f02
Ewg020h01
SC24.8
Ewg012c06
Ewg001g05
Ewg001g06
SC24.9
Ewg024d12
SC24.10
SC24.11
SC24.12

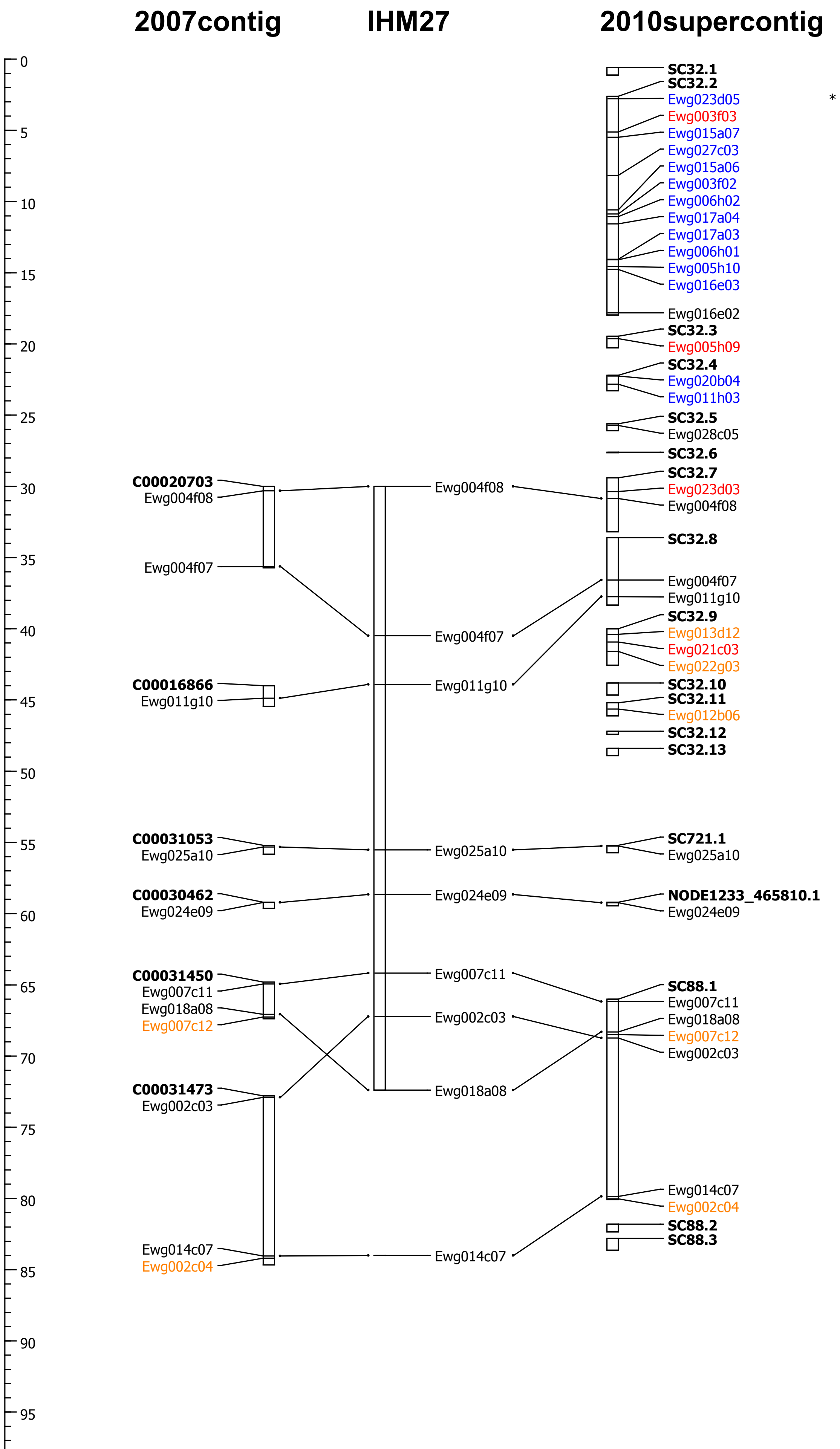
2007contig
IHM27
2010supercontig
0
5
10
15
20
25
30
35
40
45
50
55
60
65
70
75
80
85
90
95
C00020703
Ewg004f08
Ewg004f07
C00016866
Ewg011g10
C00031053
Ewg025a10
C00030462
Ewg024e09
C00031450
Ewg007c11
Ewg018a08
Ewg007c12
C00031473
Ewg002c03
Ewg014c07
Ewg002c04
Ewg004f08
Ewg004f07
Ewg011g10
Ewg025a10
Ewg024e09
Ewg007c11
Ewg002c03
Ewg018a08
Ewg014c07
SC32.1
SC32.2
Ewg023d05
*
Ewg003f03
Ewg015a07
Ewg027c03
Ewg015a06
Ewg003f02
Ewg006h02
Ewg017a04
Ewg017a03
Ewg006h01
Ewg005h10
Ewg016e03
Ewg016e02
SC32.3
Ewg005h09
SC32.4
Ewg020b04
Ewg011h03
SC32.5
Ewg028c05
SC32.6
SC32.7
Ewg023d03
Ewg004f08
SC32.8
Ewg004f07
Ewg011g10
SC32.9
Ewg013d12
Ewg021c03
Ewg022g03
SC32.10
SC32.11
Ewg012b06
SC32.12
SC32.13
SC721.1
Ewg025a10
NODE1233_465810.1
Ewg024e09
SC88.1
Ewg007c11
Ewg018a08
Ewg007c12
Ewg002c03
Ewg014c07
Ewg002c04
SC88.2
SC88.3

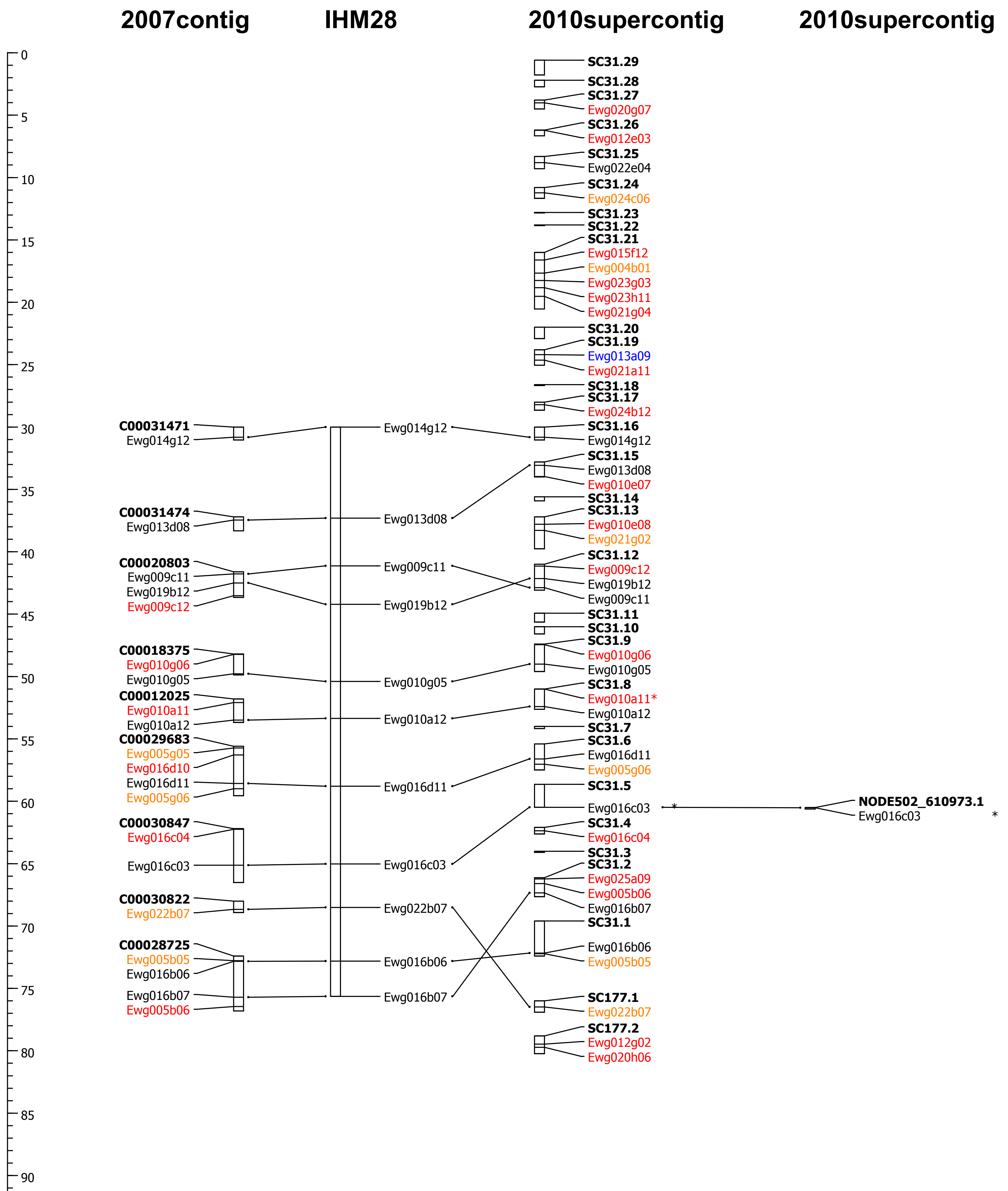

2007contig
IHM28
2010supercontig
2010supercontig
0
5
10
15
20
25
30
35
40
45
50
55
60
65
70
75
80
85
90
C00031471
Ewg014g12
C00031474
Ewg013d08
C00020803
Ewg009c11
Ewg019b12
Ewg009c12
C00018375
Ewg010g06
Ewg010g05
C00012025
Ewg010a11
Ewg010a12
C00029683
Ewg005g05
Ewg016d10
Ewg016d11
Ewg005g06
C00030847
Ewg016c04
Ewg016c03
C00030822
Ewg022b07
C00028725
Ewg005b05
Ewg016b06
Ewg016b07
Ewg005b06
Ewg014g12
Ewg013d08
Ewg009c11
Ewg019b12
Ewg010g05
Ewg010a12
Ewg016d11
Ewg016c03
Ewg022b07
Ewg016b06
Ewg016b07
SC31.29
SC31.28
SC31.27
Ewg020g07
SC31.26
Ewg012e03
SC31.25
Ewg022e04
SC31.24
Ewg024c06
SC31.23
SC31.22
SC31.21
Ewg015f12
Ewg004b01
Ewg023g03
Ewg023h11
Ewg021g04
SC31.20
SC31.19
Ewg013a09
Ewg021a11
SC31.18
SC31.17
Ewg024b12
SC31.16
Ewg014g12
SC31.15
Ewg013d08
Ewg010e07
SC31.14
SC31.13
Ewg010e08
Ewg021g02
SC31.12
Ewg009c12
Ewg019b12
Ewg009c11
SC31.11
SC31.10
SC31.9
Ewg010g06
Ewg010g05
SC31.8
Ewg010a11*
Ewg010a12
SC31.7
SC31.6
Ewg016d11
Ewg005g06
SC31.5
Ewg016c03 *
SC31.4
Ewg016c04
SC31.3
SC31.2
Ewg025a09
Ewg005b06
Ewg016b07
SC31.1
Ewg016b06
Ewg005b05
SC177.1
Ewg022b07
SC177.2
Ewg012g02
Ewg020h06
NODE502_610973.1
Ewg016c03 *

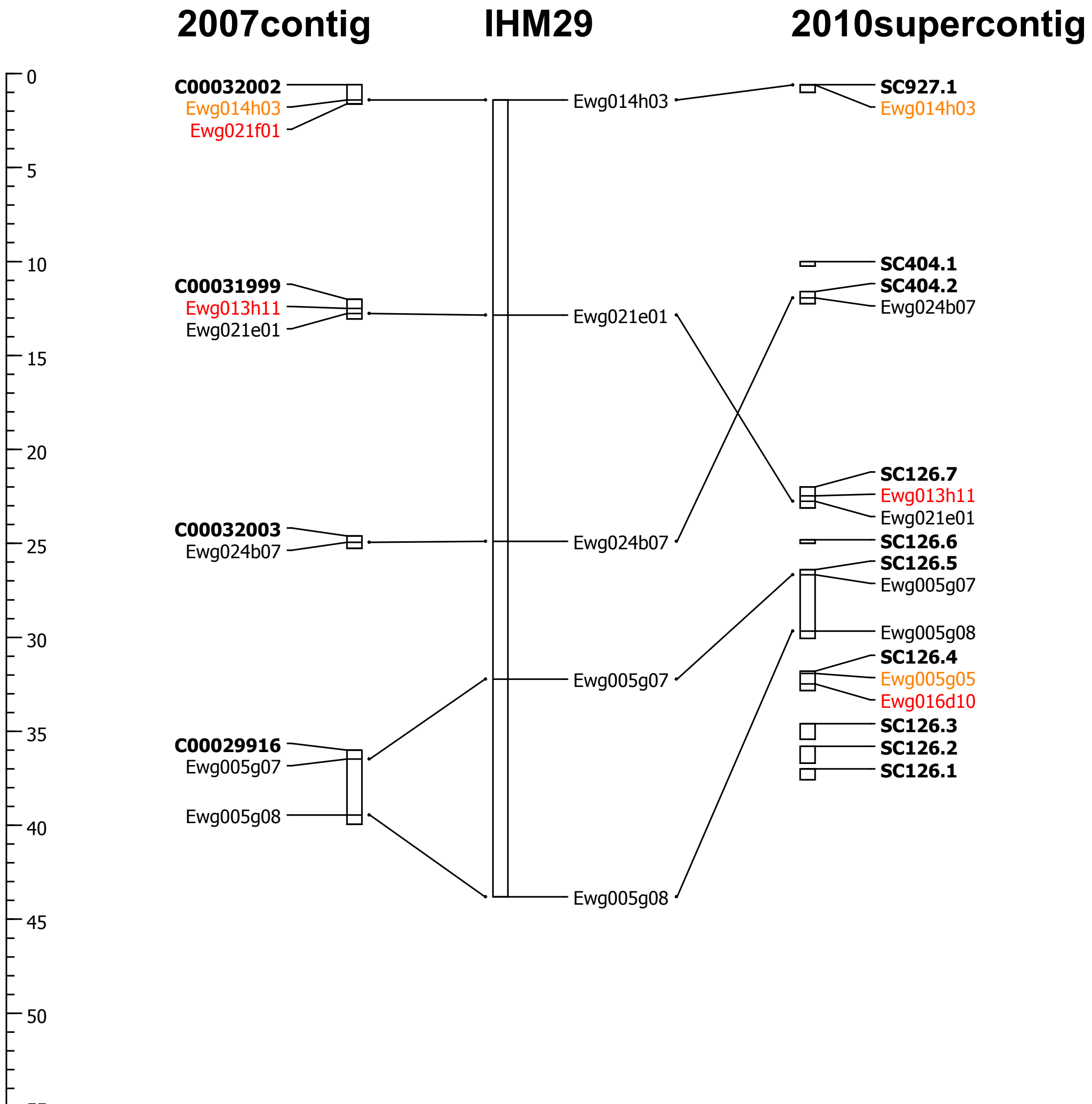
2007contig
IHM29
2010supercontig
0
5
10
15
20
25
30
35
40
45
50
C00032002
Ewg014h03
Ewg021f01
C00031999
Ewg013h11
Ewg021e01
C00032003
Ewg024b07
C00029916
Ewg005g07
Ewg005g08
Ewg014h03
Ewg021e01
Ewg024b07
Ewg005g07
Ewg005g08
SC927.1
Ewg014h03
SC404.1
SC404.2
Ewg024b07
SC126.7
Ewg013h11
Ewg021e01
SC126.6
SC126.5
Ewg005g07
Ewg005g08
SC126.4
Ewg005g05
Ewg016d10
SC126.3
SC126.2
SC126.1

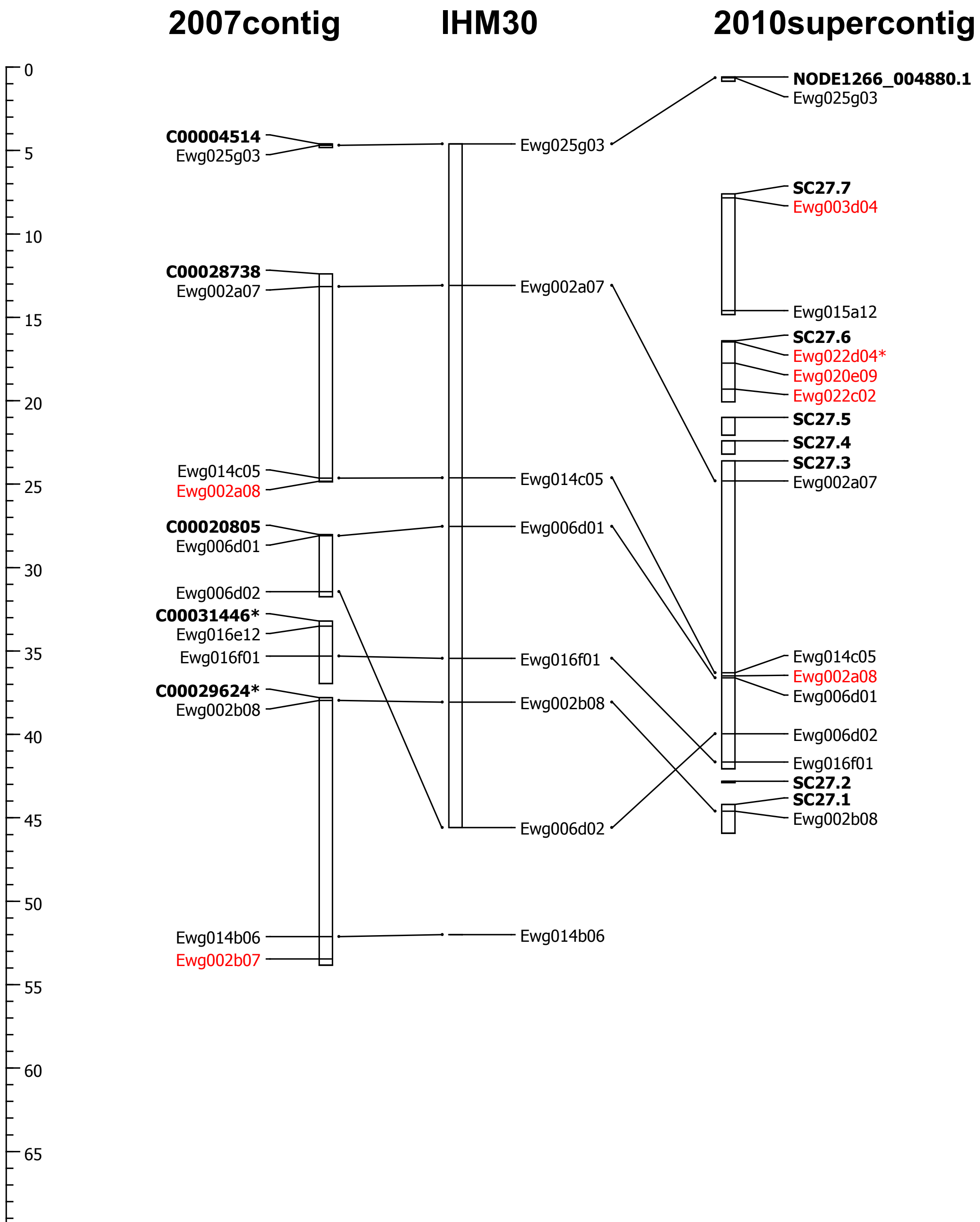
2007contig
IHM30
2010supercontig
0
5
10
15
20
25
30
35
40
45
50
55
60
65
C00004514
Ewg025g03
C00028738
Ewg002a07
Ewg014c05
Ewg002a08
C00020805
Ewg006d01
Ewg006d02
C00031446*
Ewg016e12
Ewg016f01
C00029624*
Ewg002b08
Ewg014b06
Ewg002b07
Ewg025g03
Ewg002a07
Ewg014c05
Ewg006d01
Ewg016f01
Ewg002b08
Ewg006d02
Ewg014b06
NODE1266_004880.1
Ewg025g03
SC27.7
Ewg003d04
Ewg015a12
SC27.6
Ewg022d04*
Ewg020e09
Ewg022c02
SC27.5
SC27.4
SC27.3
Ewg002a07
Ewg014c05
Ewg002a08
Ewg006d01
Ewg006d02
Ewg016f01
SC27.2
SC27.1
Ewg002b08

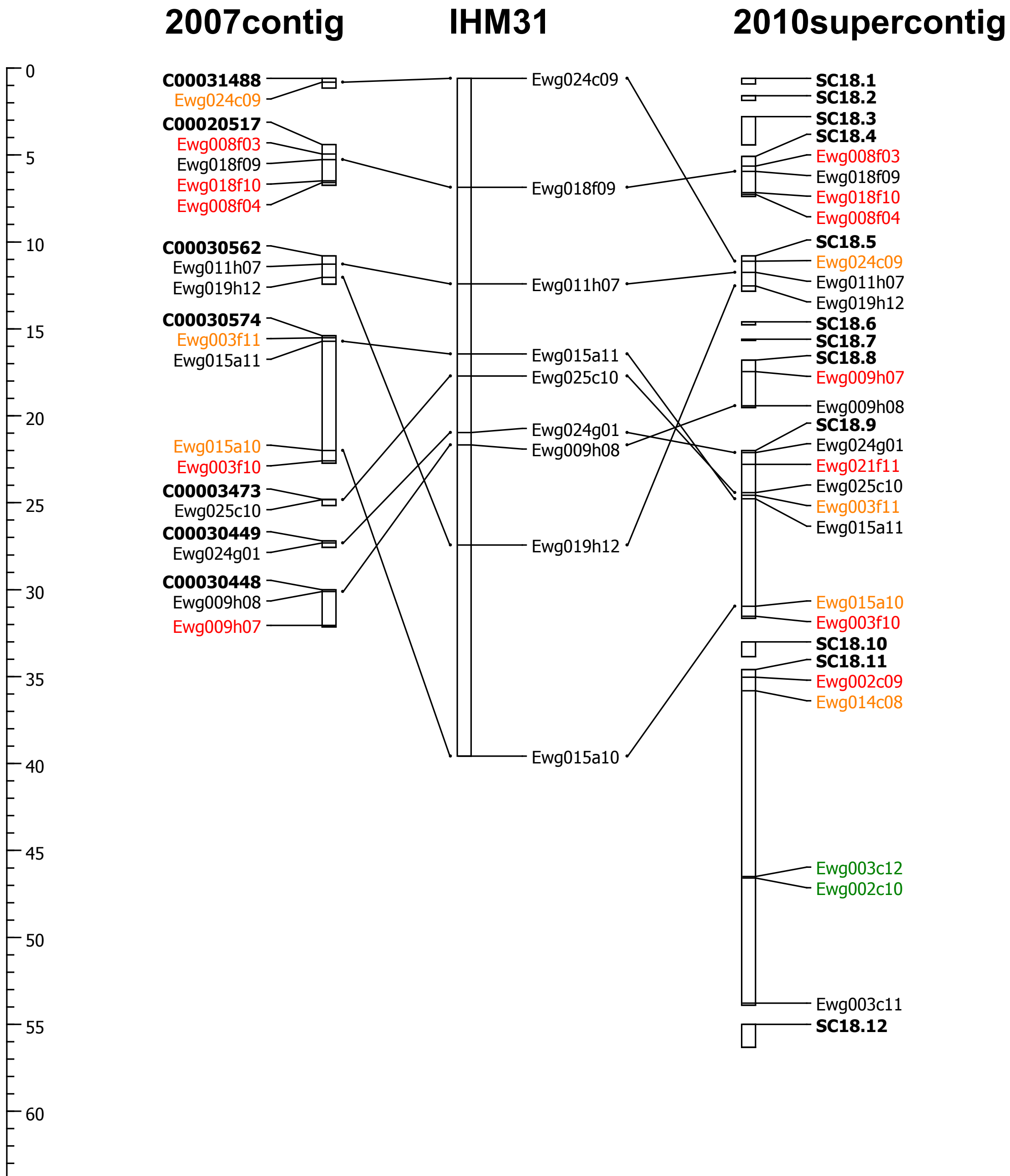

2007contig
IHM31
2010supercontig
0
5
10
15
20
25
30
35
40
45
50
55
60
C00031488
Ewg024c09
C00020517
Ewg008f03
Ewg018f09
Ewg018f10
Ewg008f04
C00030562
Ewg011h07
Ewg019h12
C00030574
Ewg003f11
Ewg015a11
Ewg015a10
Ewg003f10
C00003473
Ewg025c10
C00030449
Ewg024g01
C00030448
Ewg009h08
Ewg009h07
Ewg024c09
Ewg018f09
Ewg011h07
Ewg015a11
Ewg025c10
Ewg024g01
Ewg009h08
Ewg019h12
Ewg015a10
SC18.1
SC18.2
SC18.3
SC18.4
Ewg008f03
Ewg018f09
Ewg018f10
Ewg008f04
SC18.5
Ewg024c09
Ewg011h07
Ewg019h12
SC18.6
SC18.7
SC18.8
Ewg009h07
Ewg009h08
SC18.9
Ewg024g01
Ewg021f11
Ewg025c10
Ewg003f11
Ewg015a11
Ewg015a10
Ewg003f10
SC18.10
SC18.11
Ewg002c09
Ewg014c08
Ewg003c12
Ewg002c10
Ewg003c11
SC18.12

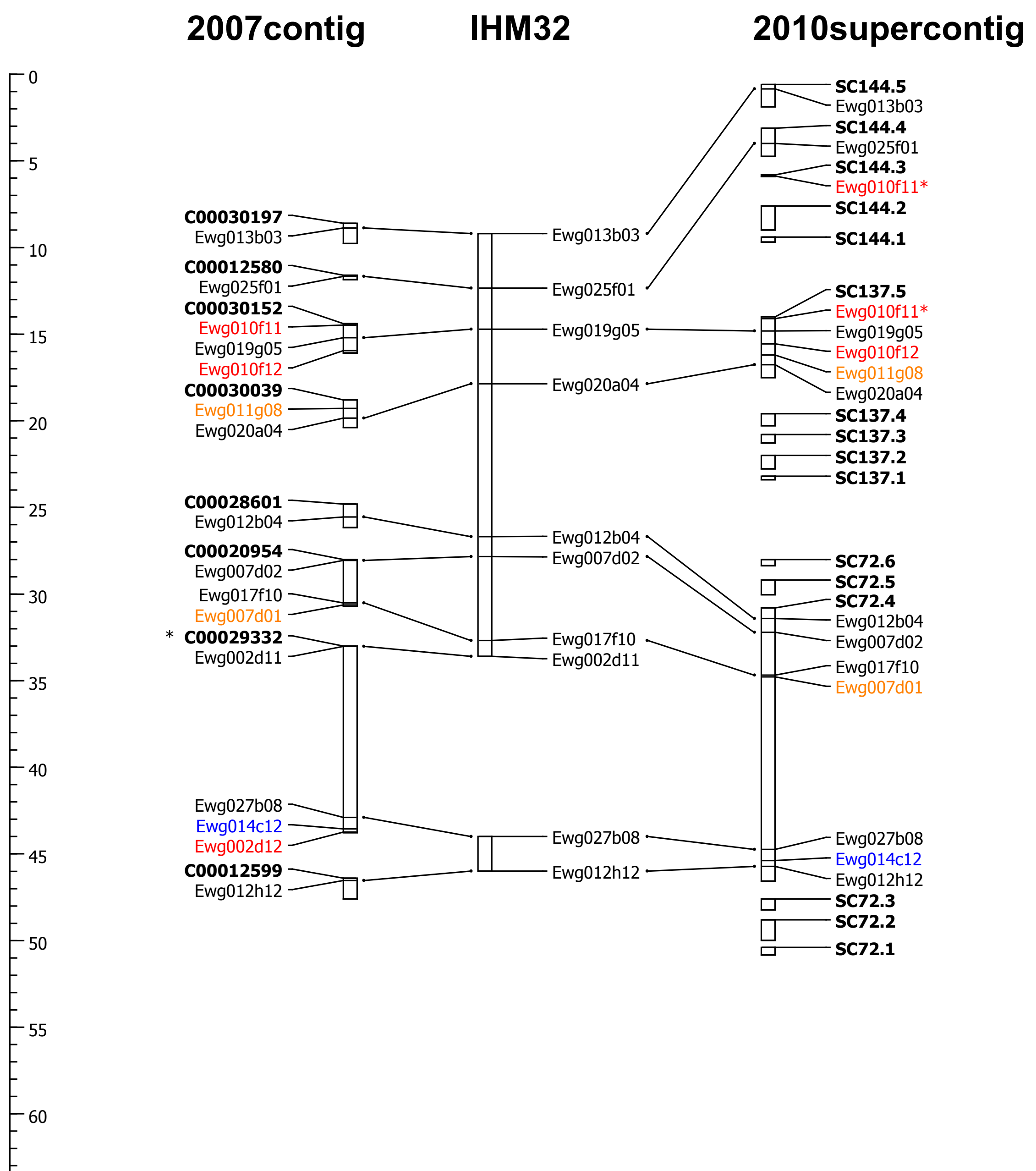

2007contig
IHM32
2010supercontig
0
5
10
15
20
25
30
35
40
45
50
55
60
C00030197
Ewg013b03
C00012580
Ewg025f01
C00030152
Ewg010f11
Ewg019g05
Ewg010f12
C00030039
Ewg011g08
Ewg020a04
C00028601
Ewg012b04
C00020954
Ewg007d02
Ewg017f10
Ewg007d01
* C00029332
Ewg002d11
Ewg027b08
Ewg014c12
Ewg002d12
C00012599
Ewg012h12
Ewg013b03
Ewg025f01
Ewg019g05
Ewg020a04
Ewg012b04
Ewg007d02
Ewg017f10
Ewg002d11
Ewg027b08
Ewg012h12
SC144.5
Ewg013b03
SC144.4
Ewg025f01
SC144.3
Ewg010f11*
SC144.2
SC144.1
SC137.5
Ewg010f11*
Ewg019g05
Ewg010f12
Ewg011g08
Ewg020a04
SC137.4
SC137.3
SC137.2
SC137.1
SC72.6
SC72.5
SC72.4
Ewg012b04
Ewg007d02
Ewg017f10
Ewg007d01
Ewg027b08
Ewg014c12
Ewg012h12
SC72.3
SC72.2
SC72.1

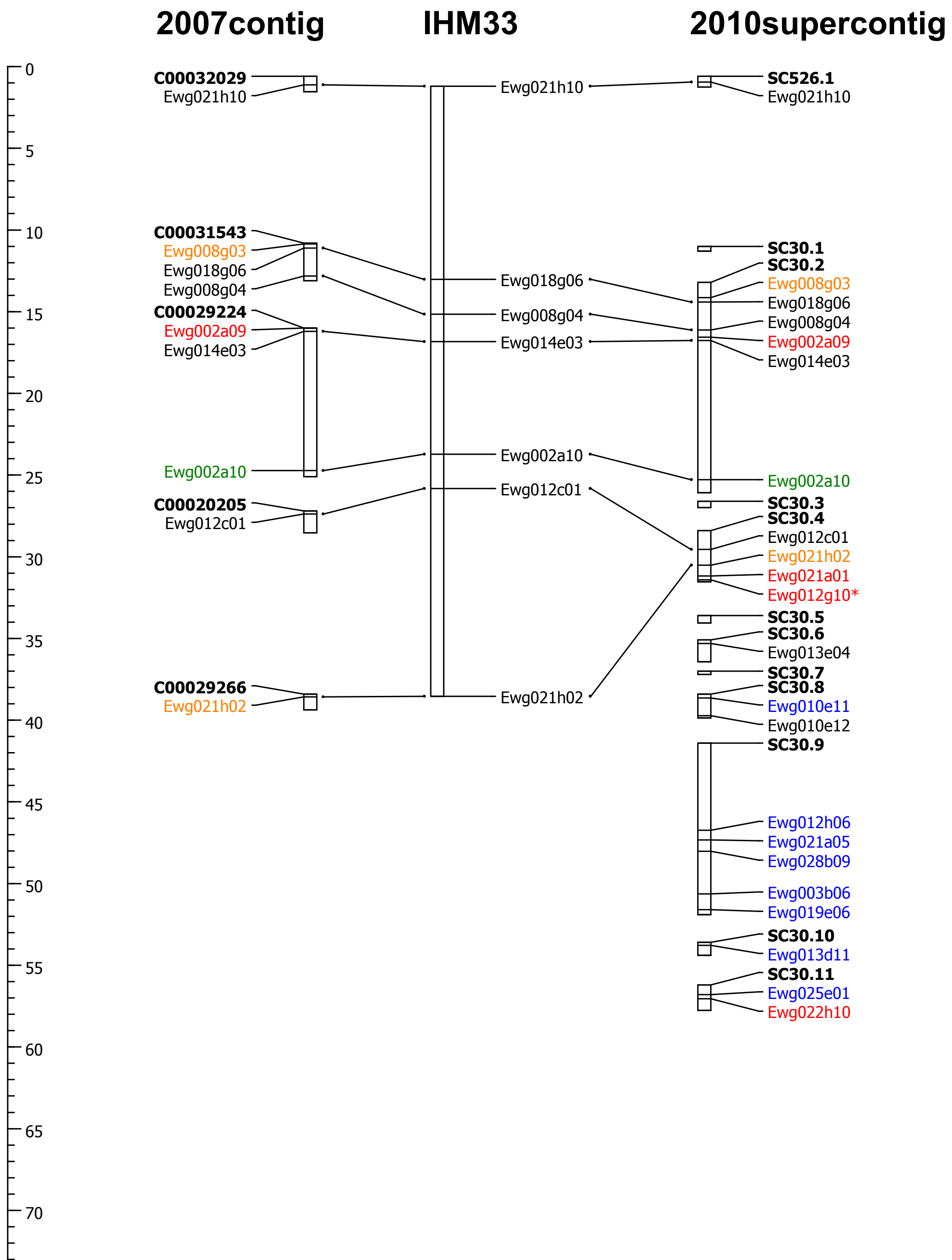

2007contig
IHM33
2010supercontig
0
5
10
15
20
25
30
35
40
45
50
55
60
65
70
C00032029
Ewg021h10
C00031543
Ewg008g03
Ewg018g06
Ewg008g04
C00029224
Ewg002a09
Ewg014e03
Ewg002a10
C00020205
Ewg012c01
C00029266
Ewg021h02
Ewg021h10
Ewg018g06
Ewg008g04
Ewg014e03
Ewg002a10
Ewg012c01
Ewg021h02
SC526.1
Ewg021h10
SC30.1
SC30.2
Ewg008g03
Ewg018g06
Ewg008g04
Ewg002a09
Ewg014e03
Ewg002a10
SC30.3
SC30.4
Ewg012c01
Ewg021h02
Ewg021a01
Ewg012g10*
SC30.5
SC30.6
Ewg013e04
SC30.7
SC30.8
Ewg010e11
Ewg010e12
SC30.9
Ewg012h06
Ewg021a05
Ewg028b09
Ewg003b06
Ewg019e06
SC30.10
Ewg013d11
SC30.11
Ewg025e01
Ewg022h10

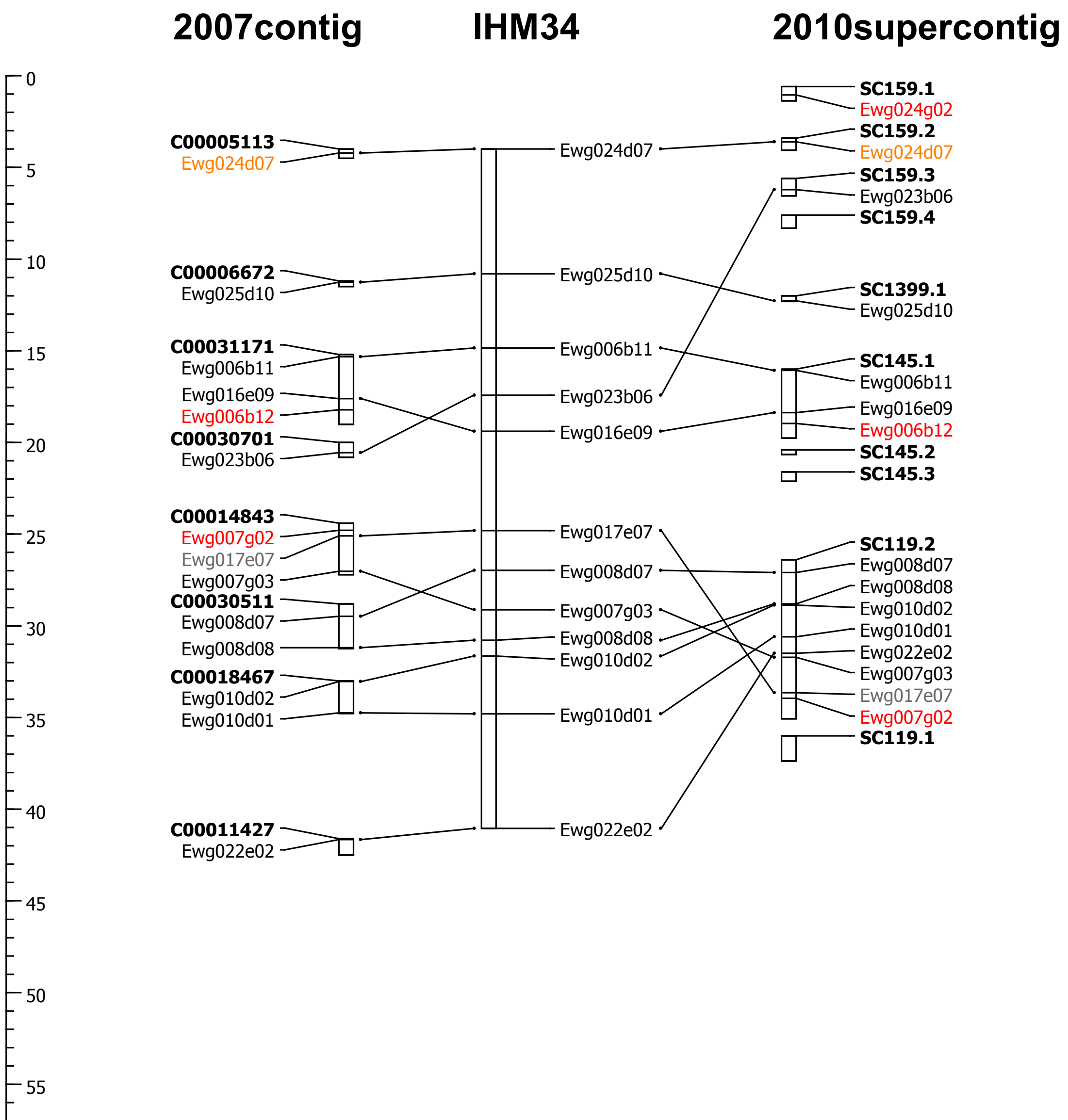

2007contig
IHM34
2010supercontig
0
5
10
15
20
25
30
35
40
45
50
55
C00005113
Ewg024d07
C00006672
Ewg025d10
C00031171
Ewg006b11
Ewg016e09
Ewg006b12
C00030701
Ewg023b06
C00014843
Ewg007g02
Ewg017e07
Ewg007g03
C00030511
Ewg008d07
Ewg008d08
C00018467
Ewg010d02
Ewg010d01
C00011427
Ewg022e02
Ewg024d07
Ewg025d10
Ewg006b11
Ewg023b06
Ewg016e09
Ewg017e07
Ewg008d07
Ewg007g03
Ewg008d08
Ewg010d02
Ewg010d01
Ewg022e02
SC159.1
Ewg024g02
SC159.2
Ewg024d07
SC159.3
Ewg023b06
SC159.4
SC1399.1
Ewg025d10
SC145.1
Ewg006b11
Ewg016e09
Ewg006b12
SC145.2
SC145.3
SC119.2
Ewg008d07
Ewg008d08
Ewg010d02
Ewg010d01
Ewg022e02
Ewg007g03
Ewg017e07
Ewg007g02
SC119.1

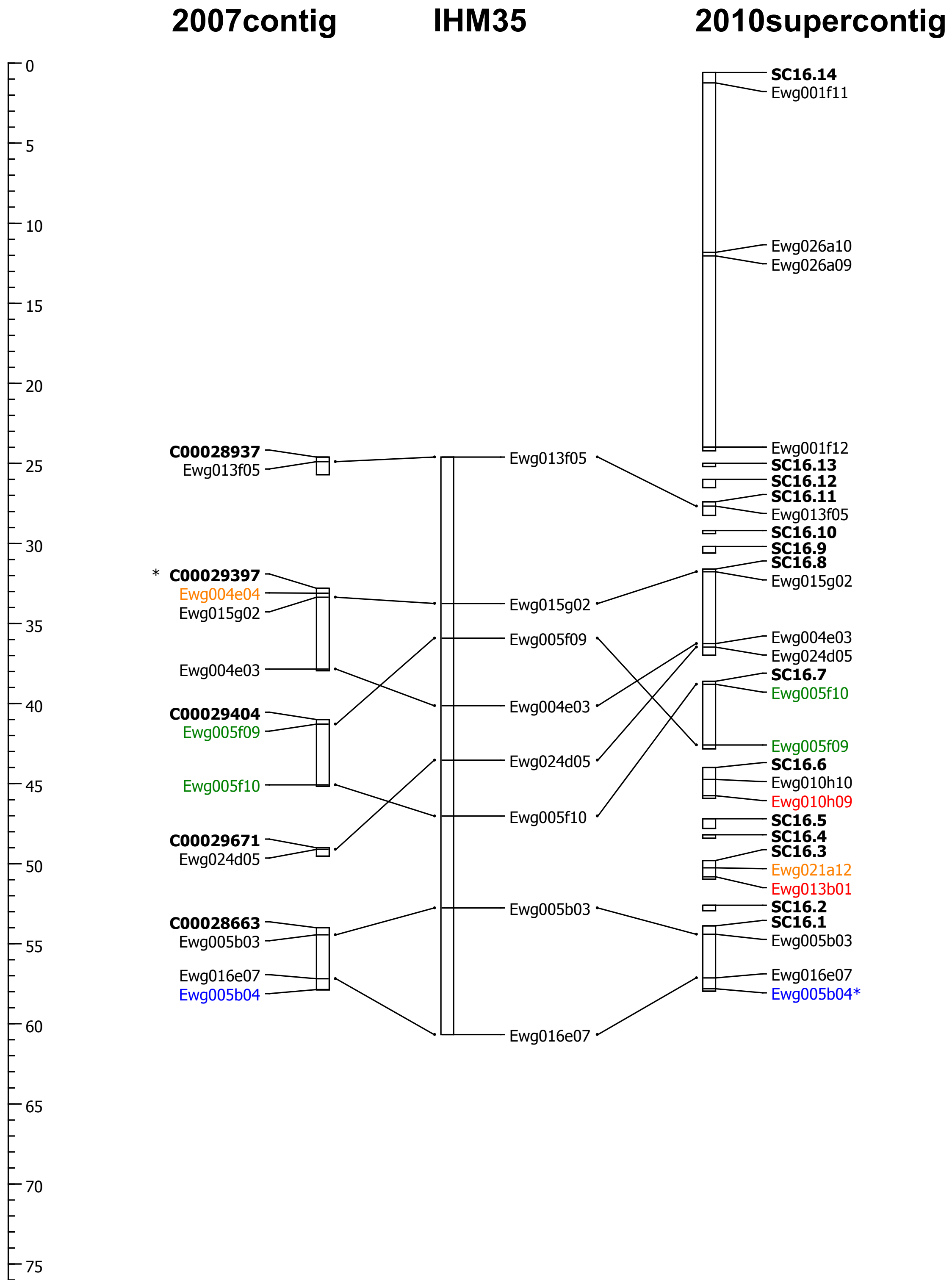

2007contig
IHM35
2010supercontig
0
5
10
15
20
25
30
35
40
45
50
55
60
65
70
75
C00028937
Ewg013f05
* C00029397
Ewg004e04
Ewg015g02
Ewg004e03
C00029404
Ewg005f09
Ewg005f10
C00029671
Ewg024d05
C00028663
Ewg005b03
Ewg016e07
Ewg005b04
Ewg013f05
Ewg015g02
Ewg005f09
Ewg004e03
Ewg024d05
Ewg005f10
Ewg005b03
Ewg016e07
SC16.14
Ewg001f11
Ewg026a10
Ewg026a09
Ewg001f12
SC16.13
SC16.12
SC16.11
Ewg013f05
SC16.10
SC16.9
SC16.8
Ewg015g02
Ewg004e03
Ewg024d05
SC16.7
Ewg005f10
Ewg005f09
SC16.6
Ewg010h10
Ewg010h09
SC16.5
SC16.4
SC16.3
Ewg021a12
Ewg013b01
SC16.2
SC16.1
Ewg005b03
Ewg016e07
Ewg005b04*

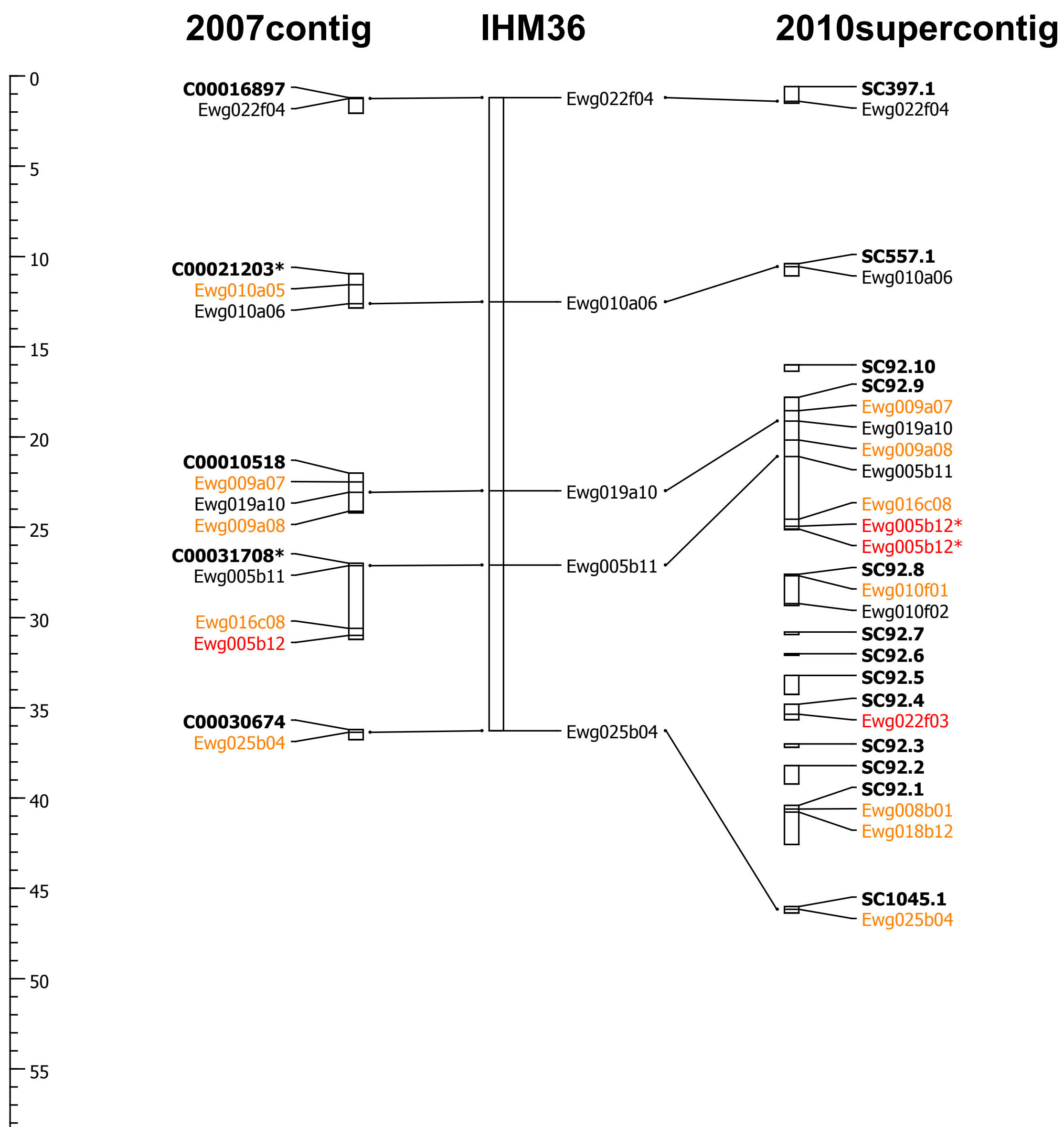

2007contig
IHM36
2010supercontig
0
5
10
15
20
25
30
35
40
45
50
55
C00016897
Ewg022f04
C00021203*
Ewg010a05
Ewg010a06
C00010518
Ewg009a07
Ewg019a10
Ewg009a08
C00031708*
Ewg005b11
Ewg016c08
Ewg005b12
C00030674
Ewg025b04
Ewg022f04
Ewg010a06
Ewg019a10
Ewg005b11
Ewg025b04
SC397.1
Ewg022f04
SC557.1
Ewg010a06
SC92.10
SC92.9
Ewg009a07
Ewg019a10
Ewg009a08
Ewg005b11
Ewg016c08
Ewg005b12*
Ewg005b12*
SC92.8
Ewg010f01
Ewg010f02
SC92.7
SC92.6
SC92.5
SC92.4
Ewg022f03
SC92.3
SC92.2
SC92.1
Ewg008b01
Ewg018b12
SC1045.1
Ewg025b04

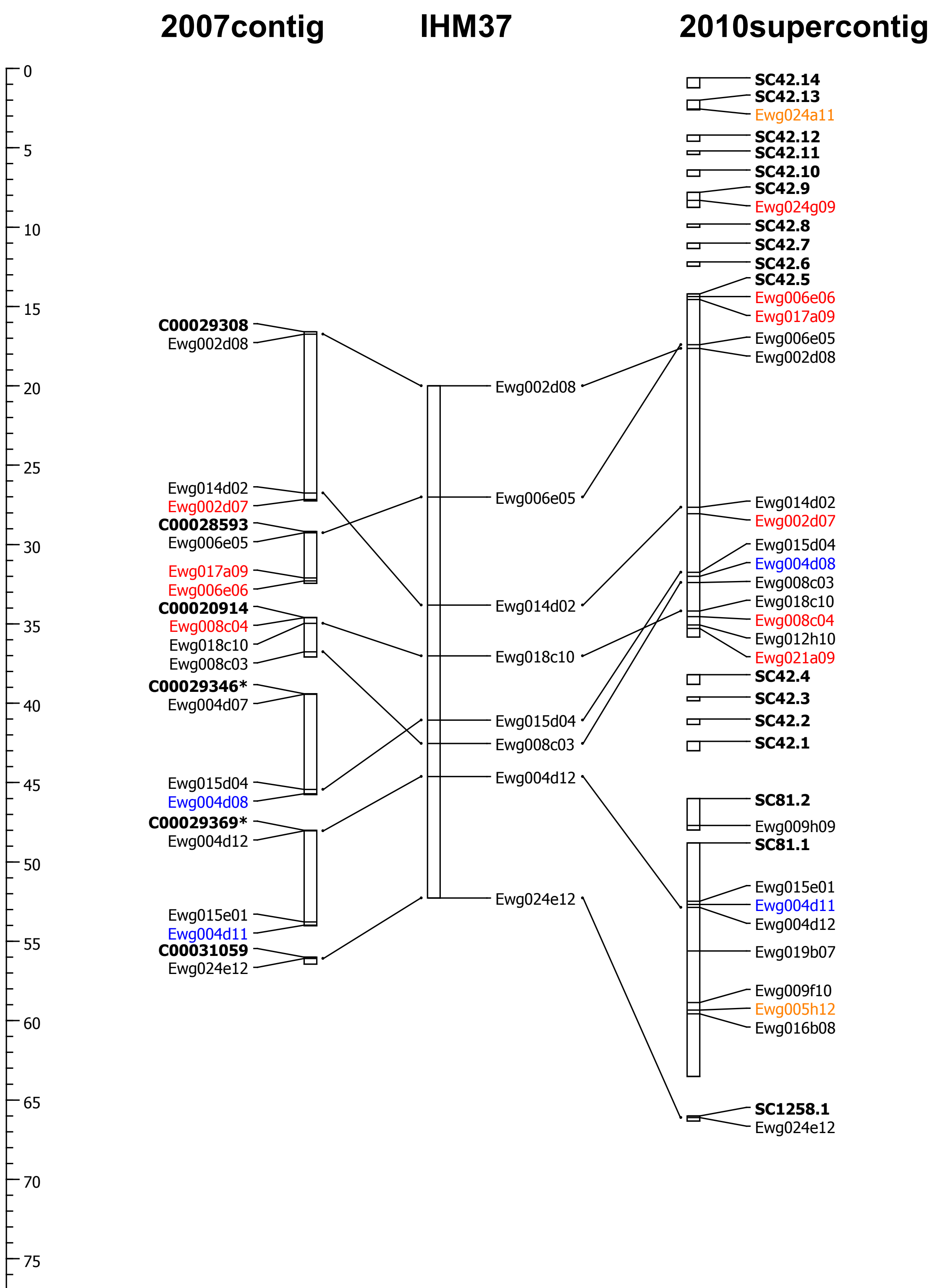

2007contig
IHM37
2010supercontig
0
5
10
15
20
25
30
35
40
45
50
55
60
65
70
75
C00029308
Ewg002d08
Ewg014d02
Ewg002d07
C00028593
Ewg006e05
Ewg017a09
Ewg006e06
C00020914
Ewg008c04
Ewg018c10
Ewg008c03
C00029346*
Ewg004d07
Ewg015d04
Ewg004d08
C00029369*
Ewg004d12
Ewg015e01
Ewg004d11
C00031059
Ewg024e12
Ewg002d08
Ewg006e05
Ewg014d02
Ewg018c10
Ewg015d04
Ewg008c03
Ewg004d12
Ewg024e12
SC42.14
SC42.13
Ewg024a11
SC42.12
SC42.11
SC42.10
SC42.9
Ewg024g09
SC42.8
SC42.7
SC42.6
SC42.5
Ewg006e06
Ewg017a09
Ewg006e05
Ewg002d08
Ewg014d02
Ewg002d07
Ewg015d04
Ewg004d08
Ewg008c03
Ewg018c10
Ewg008c04
Ewg012h10
Ewg021a09
SC42.4
SC42.3
SC42.2
SC42.1
SC81.2
Ewg009h09
SC81.1
Ewg015e01
Ewg004d11
Ewg004d12
Ewg019b07
Ewg009f10
Ewg005h12
Ewg016b08
SC1258.1
Ewg024e12

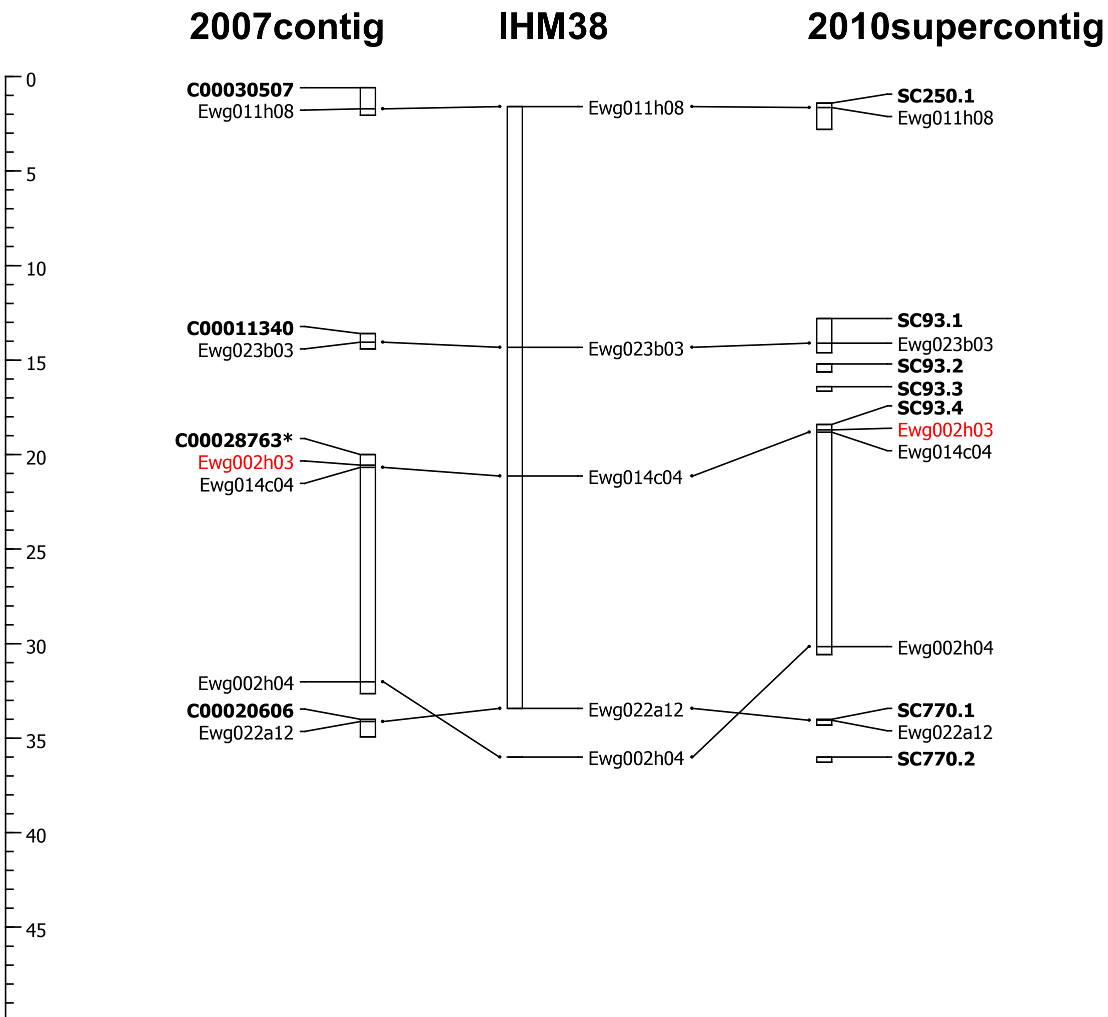
2007contig
IHM38
2010supercontig
0
5
10
15
20
25
30
35
40
45
C00030507
Ewg011h08
C00011340
Ewg023b03
C00028763*
Ewg002h03
Ewg014c04
Ewg002h04
C00020606
Ewg022a12
Ewg011h08
Ewg023b03
Ewg014c04
Ewg022a12
Ewg002h04
SC250.1
Ewg011h08
SC93.1
Ewg023b03
SC93.2
SC93.3
SC93.4
Ewg002h03
Ewg014c04
Ewg002h04
SC770.1
Ewg022a12
SC770.2

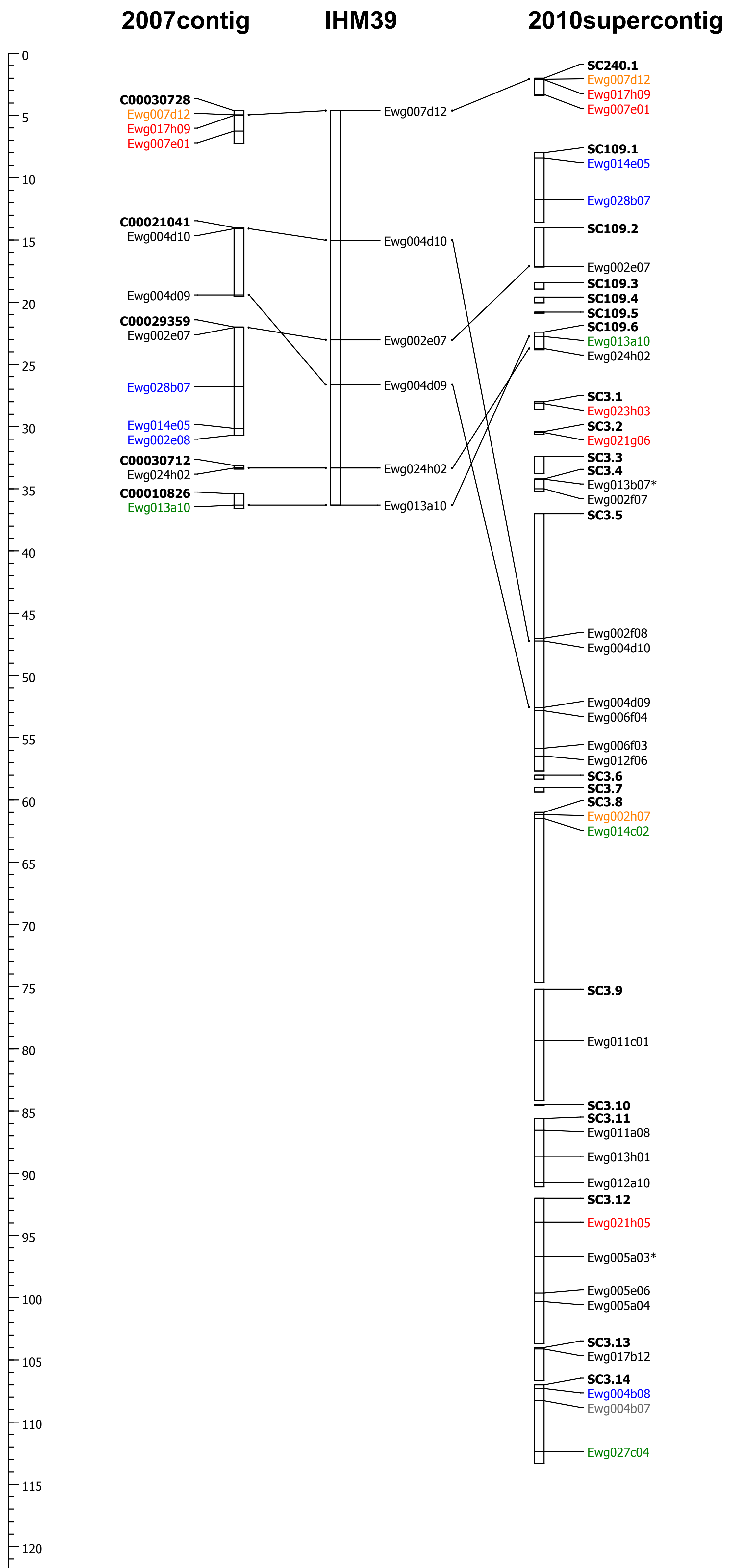

2007contig
IHM39
2010supercontig
0
5
10
15
20
25
30
35
40
45
50
55
60
65
70
75
80
85
90
95
100
105
110
115
120
C00030728
Ewg007d12
Ewg017h09
Ewg007e01
C00021041
Ewg004d10
Ewg004d09
C00029359
Ewg002e07
Ewg028b07
Ewg014e05
Ewg002e08
C00030712
Ewg024h02
C00010826
Ewg013a10
Ewg007d12
Ewg004d10
Ewg002e07
Ewg004d09
Ewg024h02
Ewg013a10
SC240.1
Ewg007d12
Ewg017h09
Ewg007e01
SC109.1
Ewg014e05
Ewg028b07
SC109.2
Ewg002e07
SC109.3
SC109.4
SC109.5
SC109.6
Ewg013a10
Ewg024h02
SC3.1
Ewg023h03
SC3.2
Ewg021g06
SC3.3
SC3.4
Ewg013b07*
Ewg002f07
SC3.5
Ewg002f08
Ewg004d10
Ewg004d09
Ewg006f04
Ewg006f03
Ewg012f06
SC3.6
SC3.7
SC3.8
Ewg002h07
Ewg014c02
SC3.9
Ewg011c01
SC3.10
SC3.11
Ewg011a08
Ewg013h01
Ewg012a10
SC3.12
Ewg021h05
Ewg005a03*
Ewg005e06
Ewg005a04
SC3.13
Ewg017b12
SC3.14
Ewg004b08
Ewg004b07
Ewg027c04

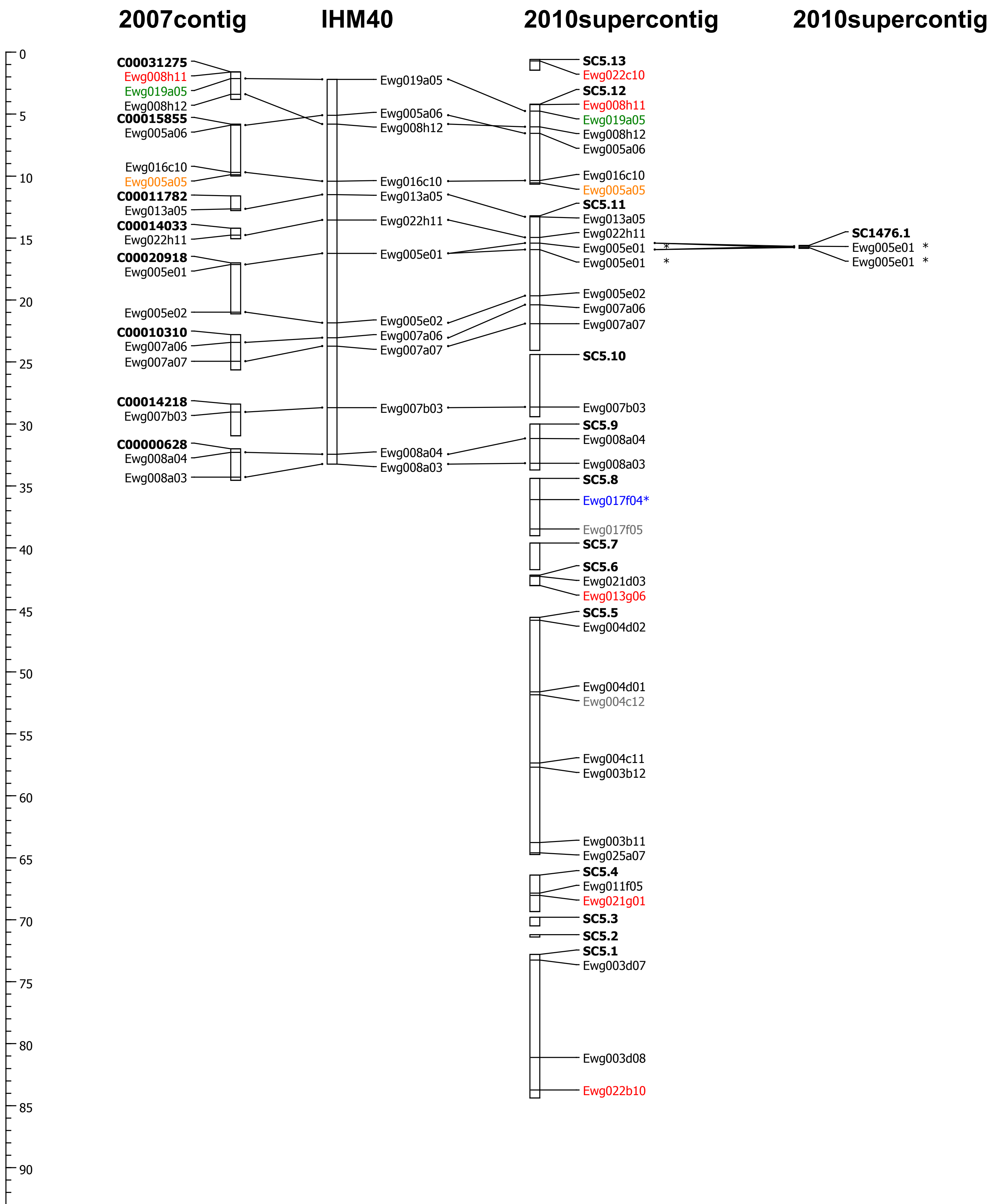
2007contig
IHM40
2010supercontig
2010supercontig
C00031275
Ewg008h11
Ewg019a05
Ewg008h12
C00015855
Ewg005a06
Ewg016c10
Ewg005a05
C00011782
Ewg013a05
C00014033
Ewg022h11
C00020918
Ewg005e01
Ewg005e02
C00010310
Ewg007a06
Ewg007a07
C00014218
Ewg007b03
C00000628
Ewg008a04
Ewg008a03
Ewg019a05
Ewg005a06
Ewg008h12
Ewg016c10
Ewg013a05
Ewg022h11
Ewg005e01
Ewg005e02
Ewg007a06
Ewg007a07
Ewg007b03
Ewg008a04
Ewg008a03
SC5.13
Ewg022c10
SC5.12
Ewg008h11
Ewg019a05
Ewg008h12
Ewg005a06
Ewg016c10
Ewg005a05
SC5.11
Ewg013a05
Ewg022h11
Ewg005e01 *
Ewg005e01 *
Ewg005e02
Ewg007a06
Ewg007a07
SC5.10
Ewg007b03
SC5.9
Ewg008a04
Ewg008a03
SC5.8
Ewg017f04*
Ewg017f05
SC5.7
SC5.6
Ewg021d03
Ewg013g06
SC5.5
Ewg004d02
Ewg004d01
Ewg004c12
Ewg004c11
Ewg003b12
Ewg003b11
Ewg025a07
SC5.4
Ewg011f05
Ewg021g01
SC5.3
SC5.2
SC5.1
Ewg003d07
Ewg003d08
Ewg022b10
SC1476.1
Ewg005e01 *
Ewg005e01 *
0
5
10
15
20
25
30
35
40
45
50
55
60
65
70
75
80
85
90

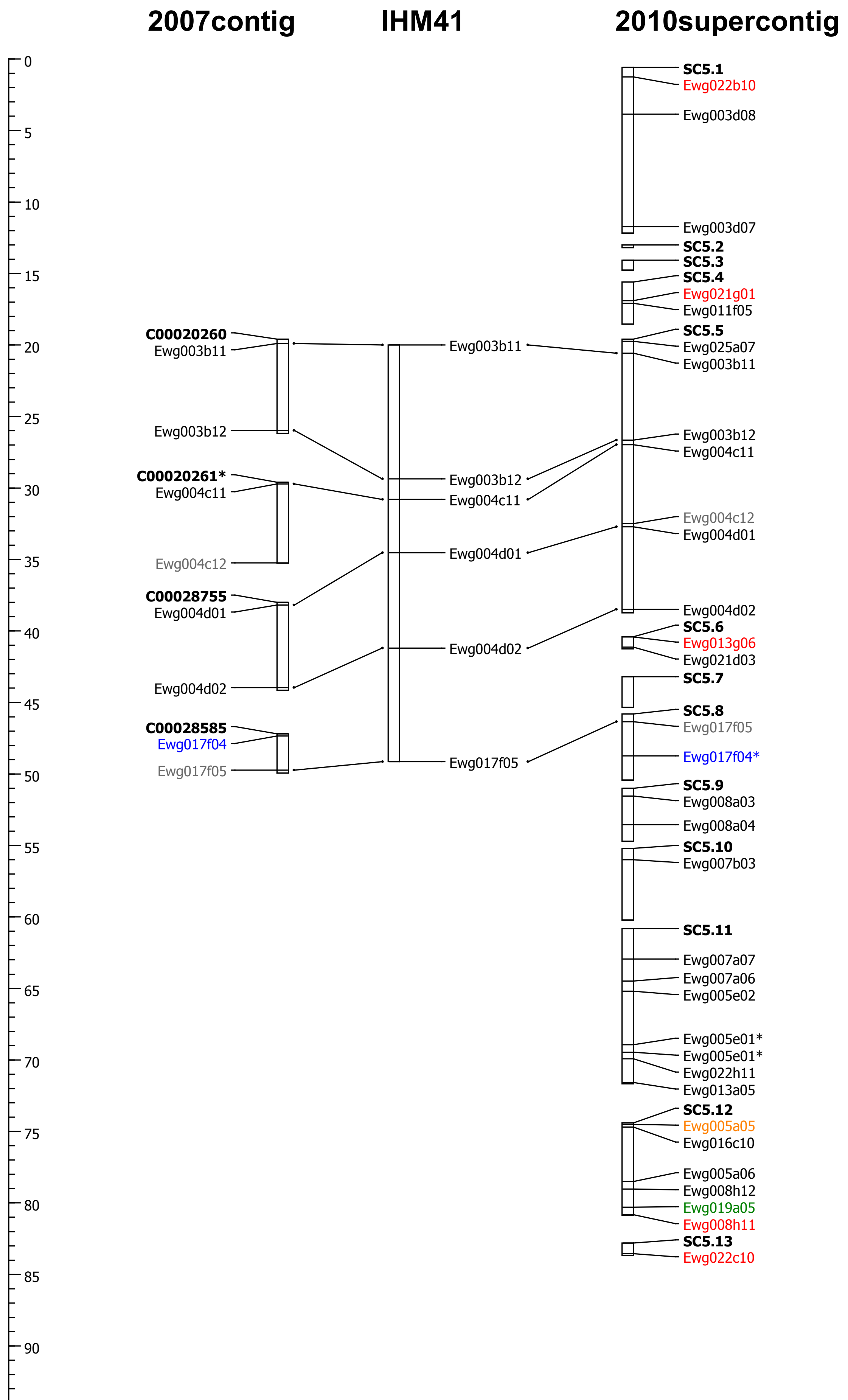

2007contig
IHM41
2010supercontig
0
5
10
15
20
25
30
35
40
45
50
55
60
65
70
75
80
85
90
C00020260
Ewg003b11
Ewg003b12
C00020261*
Ewg004c11
Ewg004c12
C00028755
Ewg004d01
Ewg004d02
C00028585
Ewg017f04
Ewg017f05
Ewg003b11
Ewg003b12
Ewg004c11
Ewg004d01
Ewg004d02
Ewg017f05
SC5.1
Ewg022b10
Ewg003d08
Ewg003d07
SC5.2
SC5.3
SC5.4
Ewg021g01
Ewg011f05
SC5.5
Ewg025a07
Ewg003b11
Ewg003b12
Ewg004c11
Ewg004c12
Ewg004d01
Ewg004d02
SC5.6
Ewg013g06
Ewg021d03
SC5.7
SC5.8
Ewg017f05
Ewg017f04*
SC5.9
Ewg008a03
Ewg008a04
SC5.10
Ewg007b03
SC5.11
Ewg007a07
Ewg007a06
Ewg005e02
Ewg005e01*
Ewg005e01*
Ewg022h11
Ewg013a05
SC5.12
Ewg005a05
Ewg016c10
Ewg005a06
Ewg008h12
Ewg019a05
Ewg008h11
SC5.13
Ewg022c10

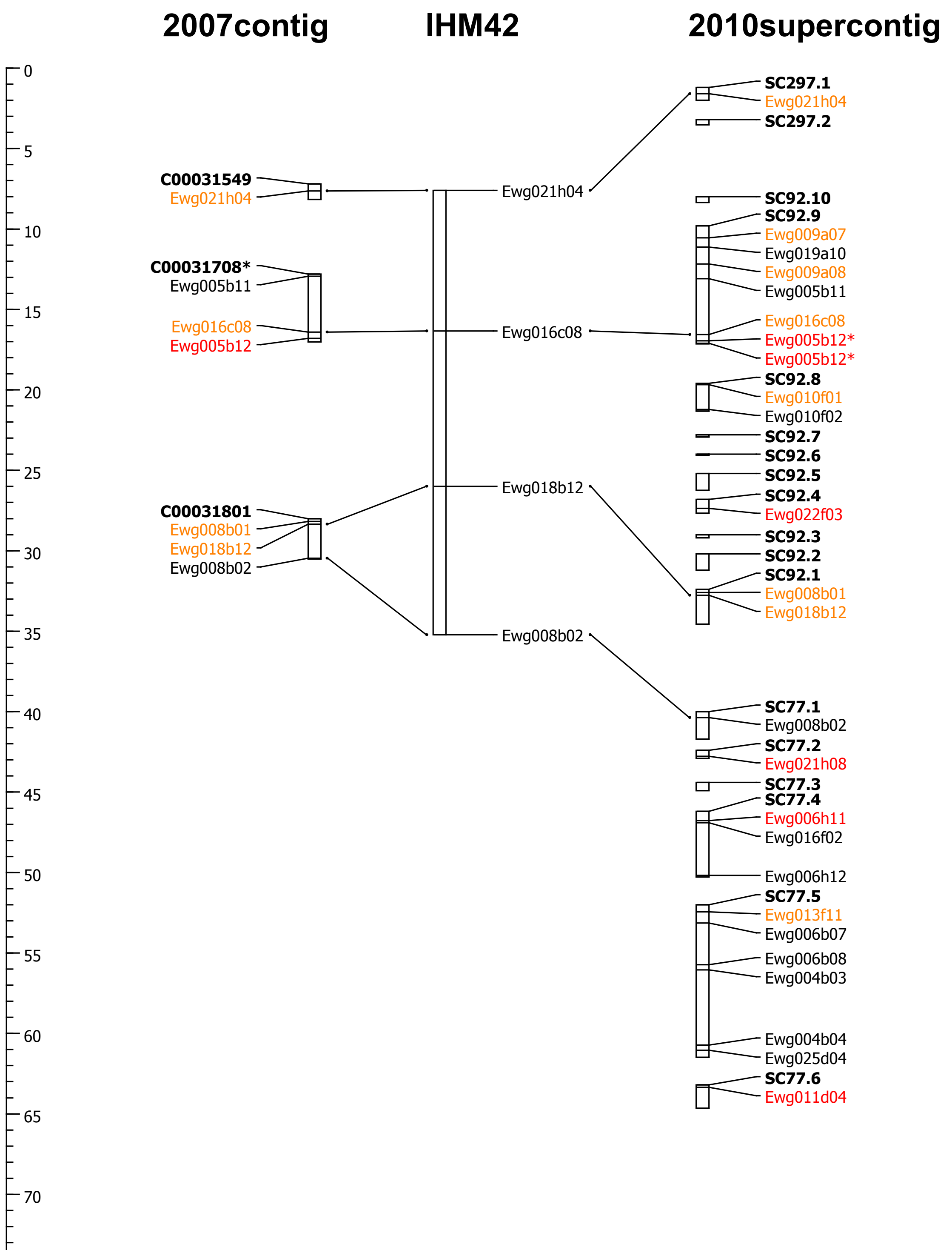
2007contig
IHM42
2010supercontig
0
5
10
15
20
25
30
35
40
45
50
55
60
65
70
C00031549
Ewg021h04
C00031708*
Ewg005b11
Ewg016c08
Ewg005b12
C00031801
Ewg008b01
Ewg018b12
Ewg008b02
Ewg021h04
Ewg016c08
Ewg018b12
Ewg008b02
SC297.1
Ewg021h04
SC297.2
SC92.10
SC92.9
Ewg009a07
Ewg019a10
Ewg009a08
Ewg005b11
Ewg016c08
Ewg005b12*
Ewg005b12*
SC92.8
Ewg010f01
Ewg010f02
SC92.7
SC92.6
SC92.5
SC92.4
Ewg022f03
SC92.3
SC92.2
SC92.1
Ewg008b01
Ewg018b12
SC77.1
Ewg008b02
SC77.2
Ewg021h08
SC77.3
SC77.4
Ewg006h11
Ewg016f02
Ewg006h12
SC77.5
Ewg013f11
Ewg006b07
Ewg006b08
Ewg004b03
Ewg004b04
Ewg025d04
SC77.6
Ewg011d04

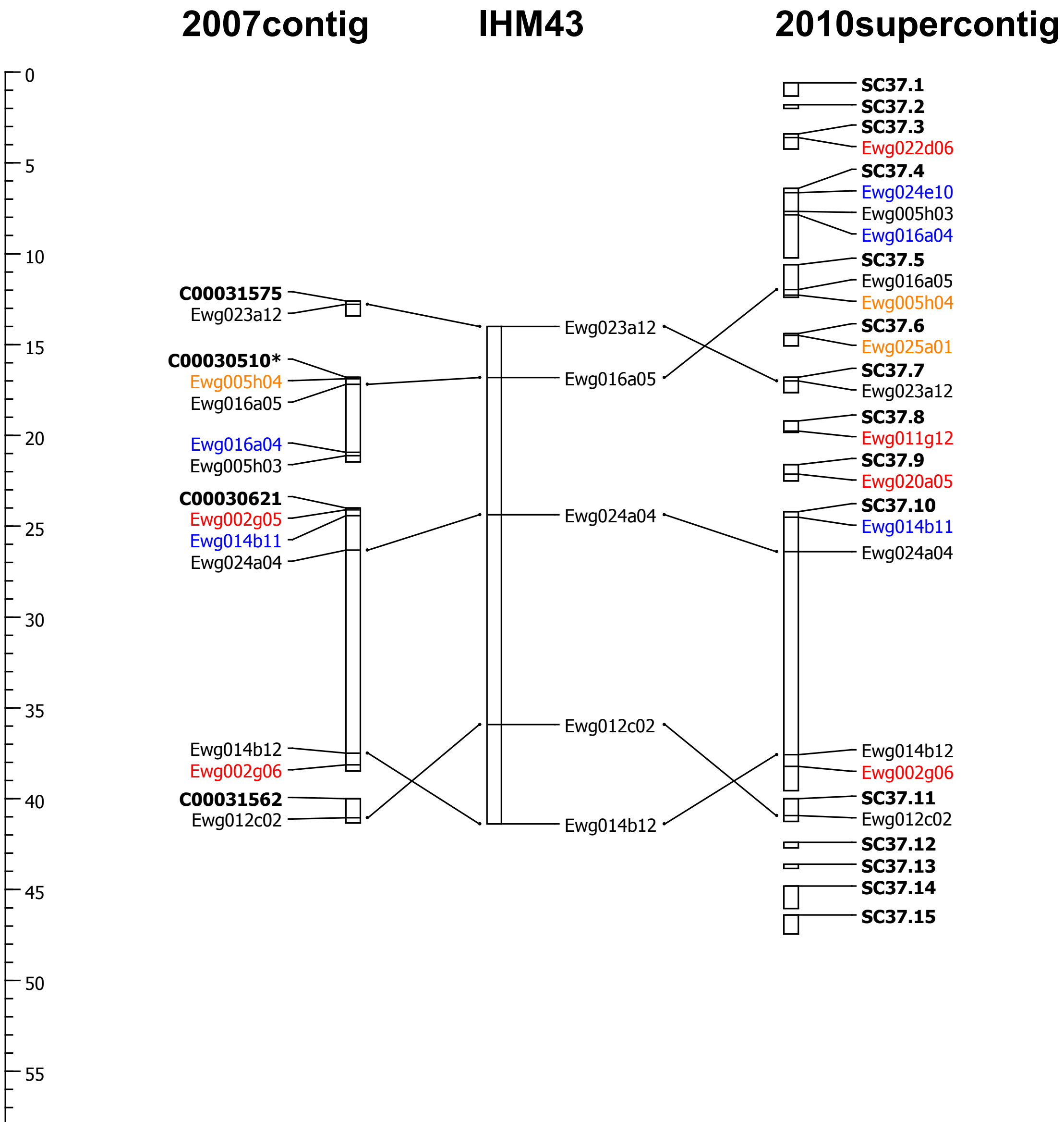
2007contig
IHM43
2010supercontig
0
5
10
15
20
25
30
35
40
45
50
55
C00031575
Ewg023a12
C00030510*
Ewg005h04
Ewg016a05
Ewg016a04
Ewg005h03
C00030621
Ewg002g05
Ewg014b11
Ewg024a04
Ewg014b12
Ewg002g06
C00031562
Ewg012c02
Ewg023a12
Ewg016a05
Ewg024a04
Ewg012c02
Ewg014b12
SC37.1
SC37.2
SC37.3
Ewg022d06
SC37.4
Ewg024e10
Ewg005h03
Ewg016a04
SC37.5
Ewg016a05
Ewg005h04
SC37.6
Ewg025a01
SC37.7
Ewg023a12
SC37.8
Ewg011g12
SC37.9
Ewg020a05
SC37.10
Ewg014b11
Ewg024a04
Ewg014b12
Ewg002g06
SC37.11
Ewg012c02
SC37.12
SC37.13
SC37.14
SC37.15

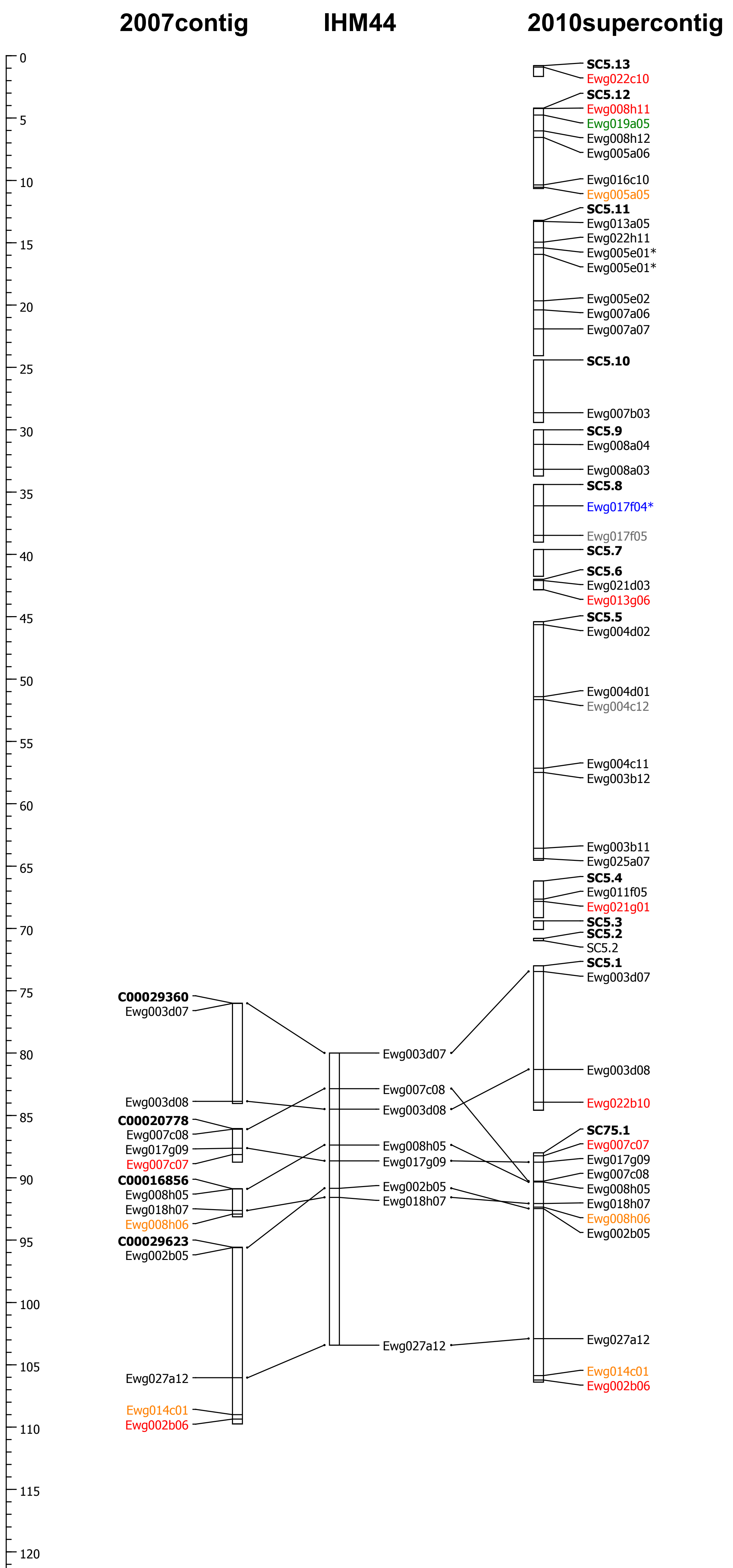

2007contig
IHM44
2010supercontig
0
5
10
15
20
25
30
35
40
45
50
55
60
65
70
75
80
85
90
95
100
105
110
115
120
SC5.13
Ewg022c10
SC5.12
Ewg008h11
Ewg019a05
Ewg008h12
Ewg005a06
Ewg016c10
Ewg005a05
SC5.11
Ewg013a05
Ewg022h11
Ewg005e01*
Ewg005e01*
Ewg005e02
Ewg007a06
Ewg007a07
SC5.10
Ewg007b03
SC5.9
Ewg008a04
Ewg008a03
SC5.8
Ewg017f04*
Ewg017f05
SC5.7
SC5.6
Ewg021d03
Ewg013g06
SC5.5
Ewg004d02
Ewg004d01
Ewg004c12
Ewg004c11
Ewg003b12
Ewg003b11
Ewg025a07
SC5.4
Ewg011f05
Ewg021g01
SC5.3
SC5.2
SC5.2
SC5.1
Ewg003d07
Ewg003d08
Ewg022b10
SC75.1
Ewg007c07
Ewg017g09
Ewg007c08
Ewg008h05
Ewg018h07
Ewg008h06
Ewg002b05
Ewg027a12
Ewg014c01
Ewg002b06
Ewg003d07
Ewg007c08
Ewg003d08
Ewg008h05
Ewg017g09
Ewg002b05
Ewg018h07
Ewg027a12
C00029360
Ewg003d07
Ewg003d08
C00020778
Ewg007c08
Ewg017g09
Ewg007c07
C00016856
Ewg008h05
Ewg018h07
Ewg008h06
C00029623
Ewg002b05
Ewg027a12
Ewg014c01
Ewg002b06

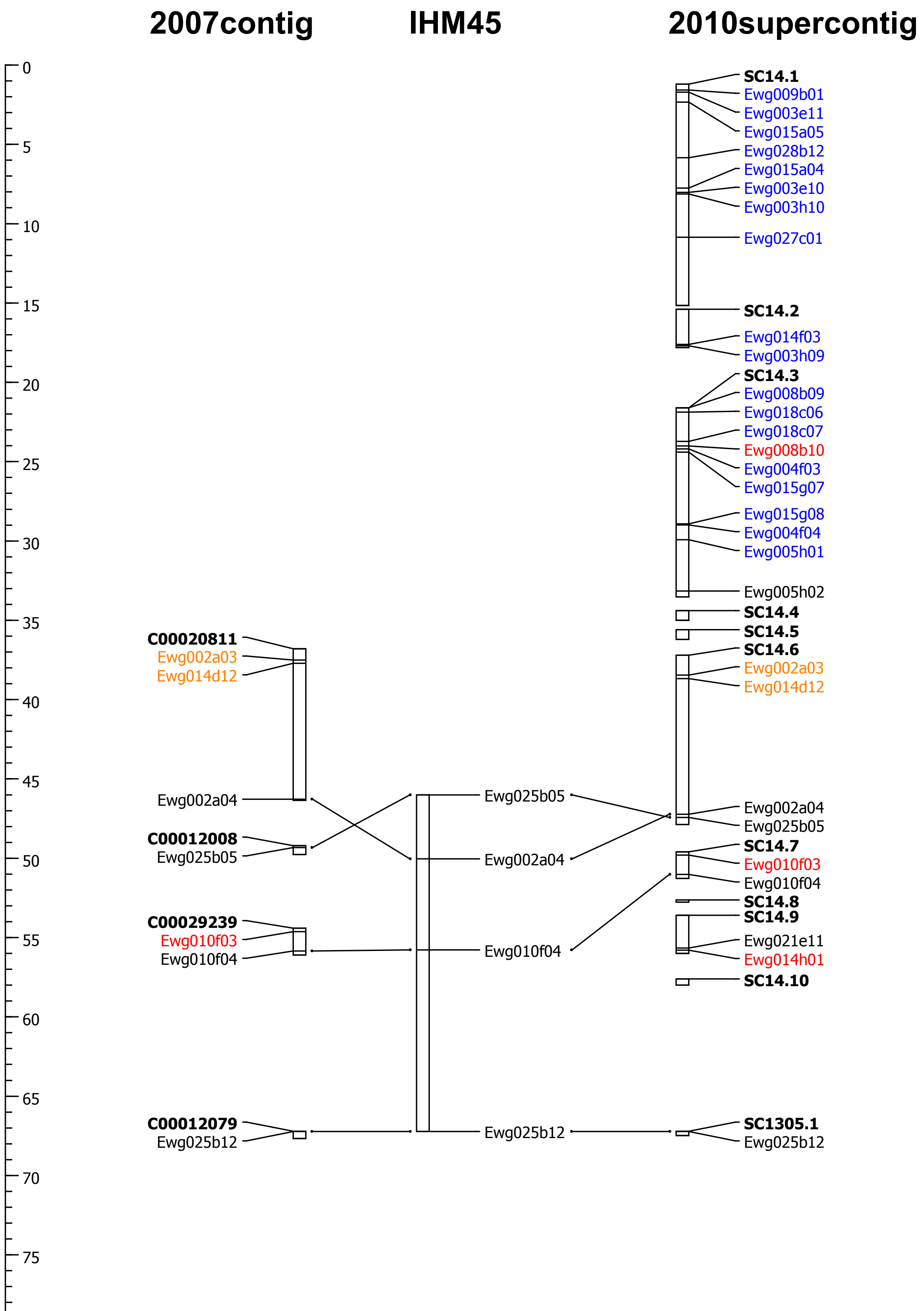

2007contig
IHM45
2010supercontig
0
5
10
15
20
25
30
35
40
45
50
55
60
65
70
75
C00020811
Ewg002a03
Ewg014d12
Ewg002a04
C00012008
Ewg025b05
C00029239
Ewg010f03
Ewg010f04
C00012079
Ewg025b12
Ewg025b05
Ewg002a04
Ewg010f04
Ewg025b12
SC14.1
Ewg009b01
Ewg003e11
Ewg015a05
Ewg028b12
Ewg015a04
Ewg003e10
Ewg003h10
Ewg027c01
SC14.2
Ewg014f03
Ewg003h09
SC14.3
Ewg008b09
Ewg018c06
Ewg018c07
Ewg008b10
Ewg004f03
Ewg015g07
Ewg015g08
Ewg004f04
Ewg005h01
Ewg005h02
SC14.4
SC14.5
SC14.6
Ewg002a03
Ewg014d12
Ewg002a04
Ewg025b05
SC14.7
Ewg010f03
Ewg010f04
SC14.8
SC14.9
Ewg021e11
Ewg014h01
SC14.10
SC1305.1
Ewg025b12

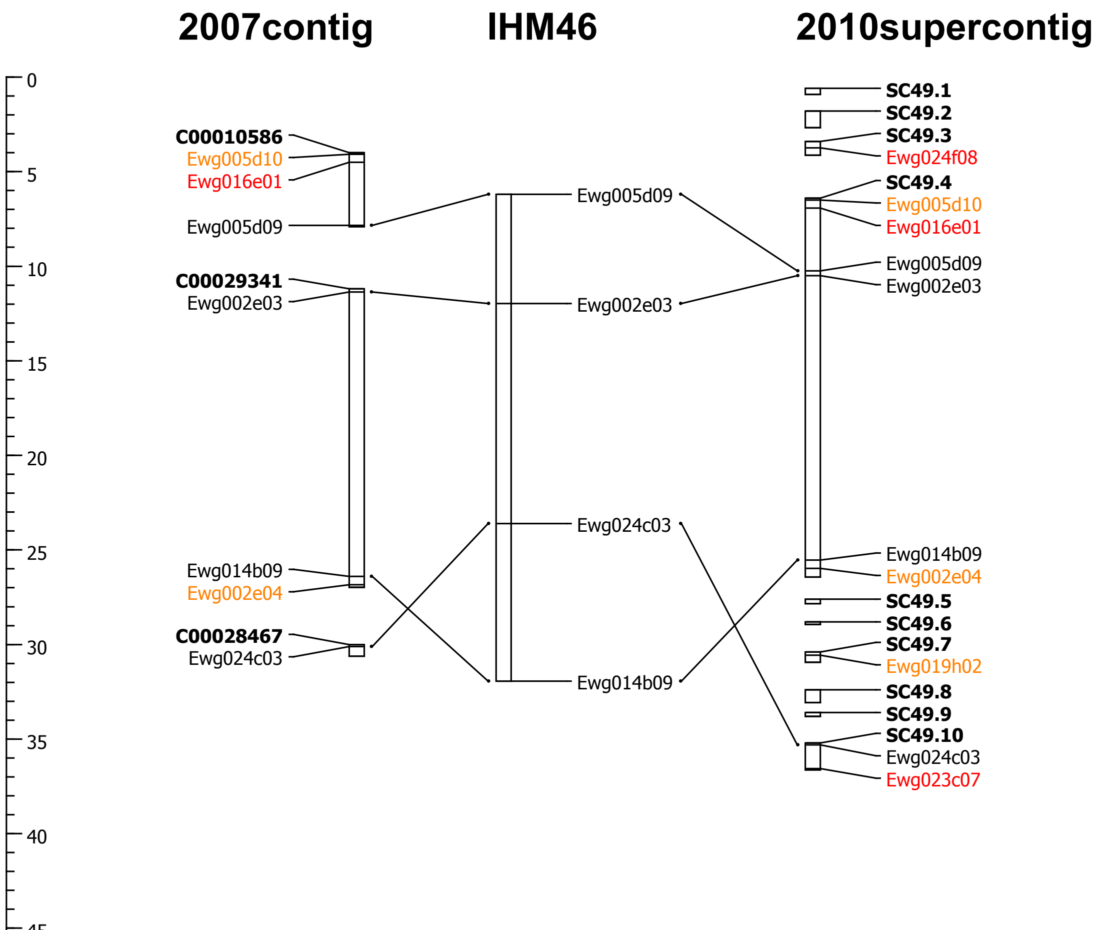
2007contig
IHM46
2010supercontig
0
5
10
15
20
25
30
35
40
C00010586
Ewg005d10
Ewg016e01
Ewg005d09
C00029341
Ewg002e03
Ewg014b09
Ewg002e04
C00028467
Ewg024c03
Ewg005d09
Ewg002e03
Ewg024c03
Ewg014b09
SC49.1
SC49.2
SC49.3
Ewg024f08
SC49.4
Ewg005d10
Ewg016e01
Ewg005d09
Ewg002e03
Ewg014b09
Ewg002e04
SC49.5
SC49.6
SC49.7
Ewg019h02
SC49.8
SC49.9
SC49.10
Ewg024c03
Ewg023c07

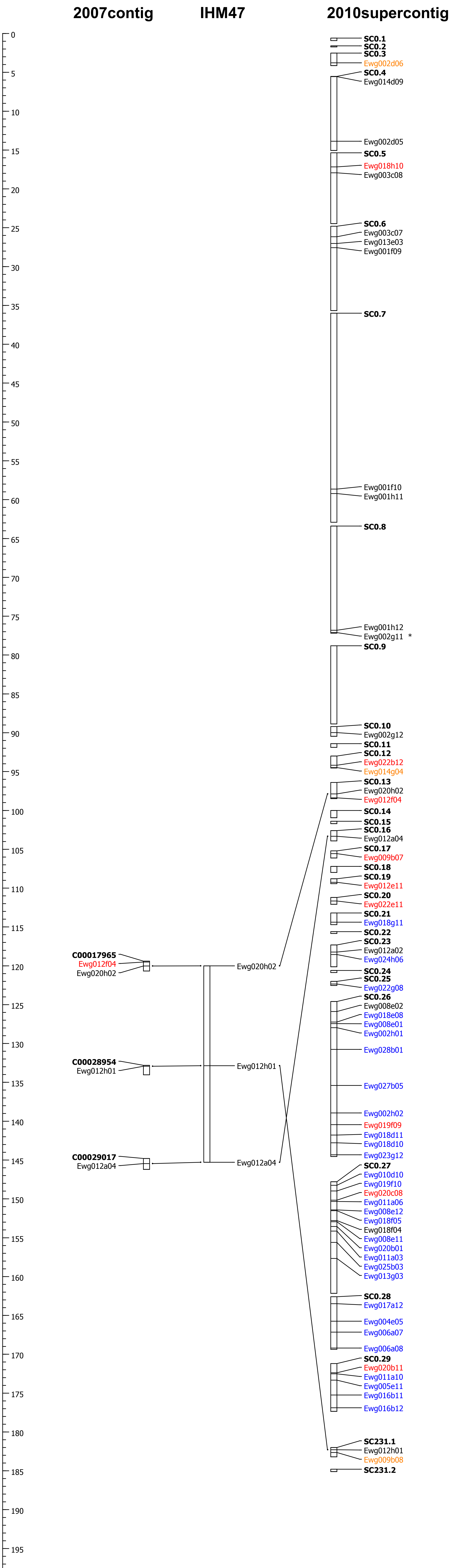
2007contig
IHM47
2010supercontig
SC0.1
SC0.2
SC0.3
Ewg002d06
SC0.4
Ewg014d09
Ewg002d05
SC0.5
Ewg018h10
Ewg003c08
SC0.6
Ewg003c07
Ewg013e03
Ewg001f09
SC0.7
Ewg001f10
Ewg001h11
SC0.8
Ewg001h12
Ewg002g11 *
SC0.9
SC0.10
Ewg002g12
SC0.11
SC0.12
Ewg022b12
Ewg014g04
SC0.13
Ewg020h02
Ewg012f04
SC0.14
SC0.15
SC0.16
Ewg012a04
SC0.17
Ewg009b07
SC0.18
SC0.19
Ewg012e11
SC0.20
Ewg022e11
SC0.21
Ewg018g11
SC0.22
SC0.23
Ewg012a02
Ewg024h06
SC0.24
SC0.25
Ewg022g08
SC0.26
Ewg008e02
Ewg018e08
Ewg008e01
Ewg002h01
Ewg028b01
Ewg027b05
Ewg002h02
Ewg019f09
Ewg018d11
Ewg018d10
Ewg023g12
SC0.27
Ewg010d10
Ewg019f10
Ewg020c08
Ewg011a06
Ewg008e12
Ewg018f05
Ewg018f04
Ewg008e11
Ewg020b01
Ewg011a03
Ewg025b03
Ewg013g03
SC0.28
Ewg017a12
Ewg004e05
Ewg006a07
Ewg006a08
SC0.29
Ewg020b11
Ewg011a10
Ewg005e11
Ewg016b11
Ewg016b12
SC231.1
Ewg012h01
Ewg009b08
SC231.2
C00017965
Ewg012f04
Ewg020h02
C00028954
Ewg012h01
C00029017
Ewg012a04
Ewg020h02
Ewg012h01
Ewg012a04
0
5
10
15
20
25
30
35
40
45
50
55
60
65
70
75
80
85
90
95
100
105
110
115
120
125
130
135
140
145
150
155
160
165
170
175
180
185
190
195

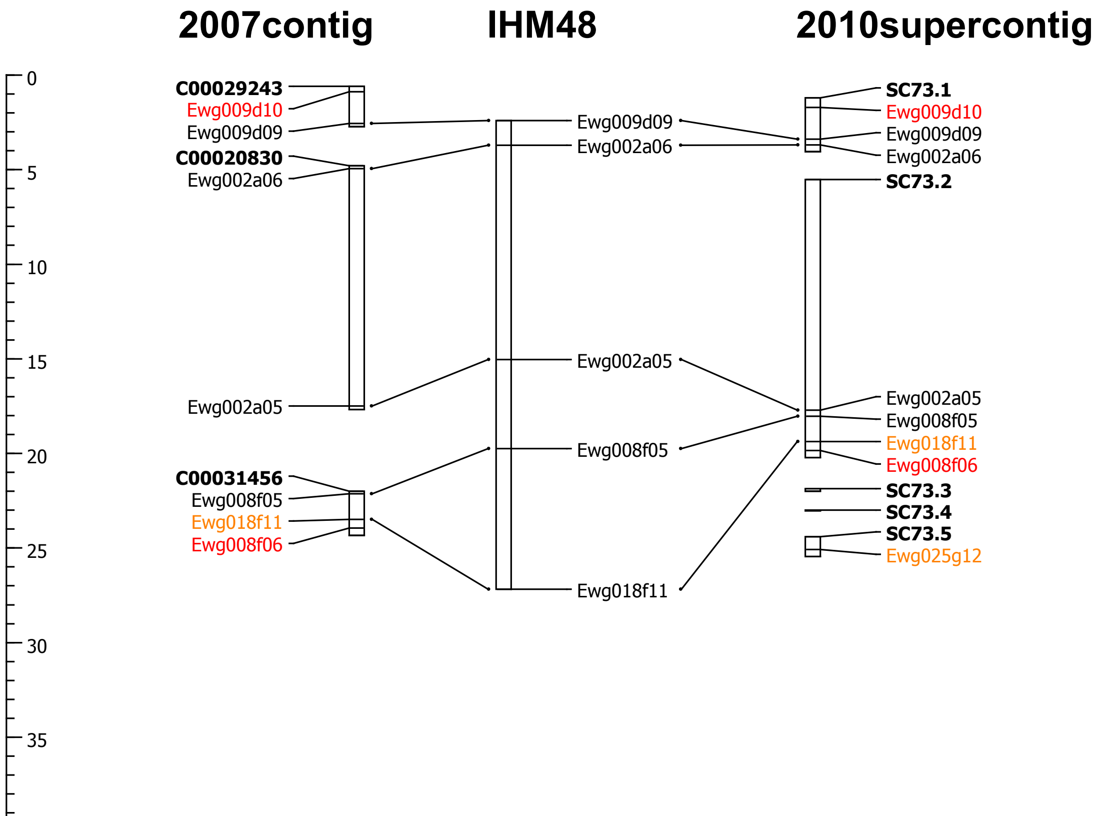

2007contig
IHM48
2010supercontig
0
5
10
15
20
25
30
35
C00029243
Ewg009d10
Ewg009d09
C00020830
Ewg002a06
Ewg002a05
C00031456
Ewg008f05
Ewg018f11
Ewg008f06
Ewg009d09
Ewg002a06
Ewg002a05
Ewg008f05
Ewg018f11
SC73.1
Ewg009d10
Ewg009d09
Ewg002a06
SC73.2
Ewg002a05
Ewg008f05
Ewg018f11
Ewg008f06
SC73.3
SC73.4
SC73.5
Ewg025g12

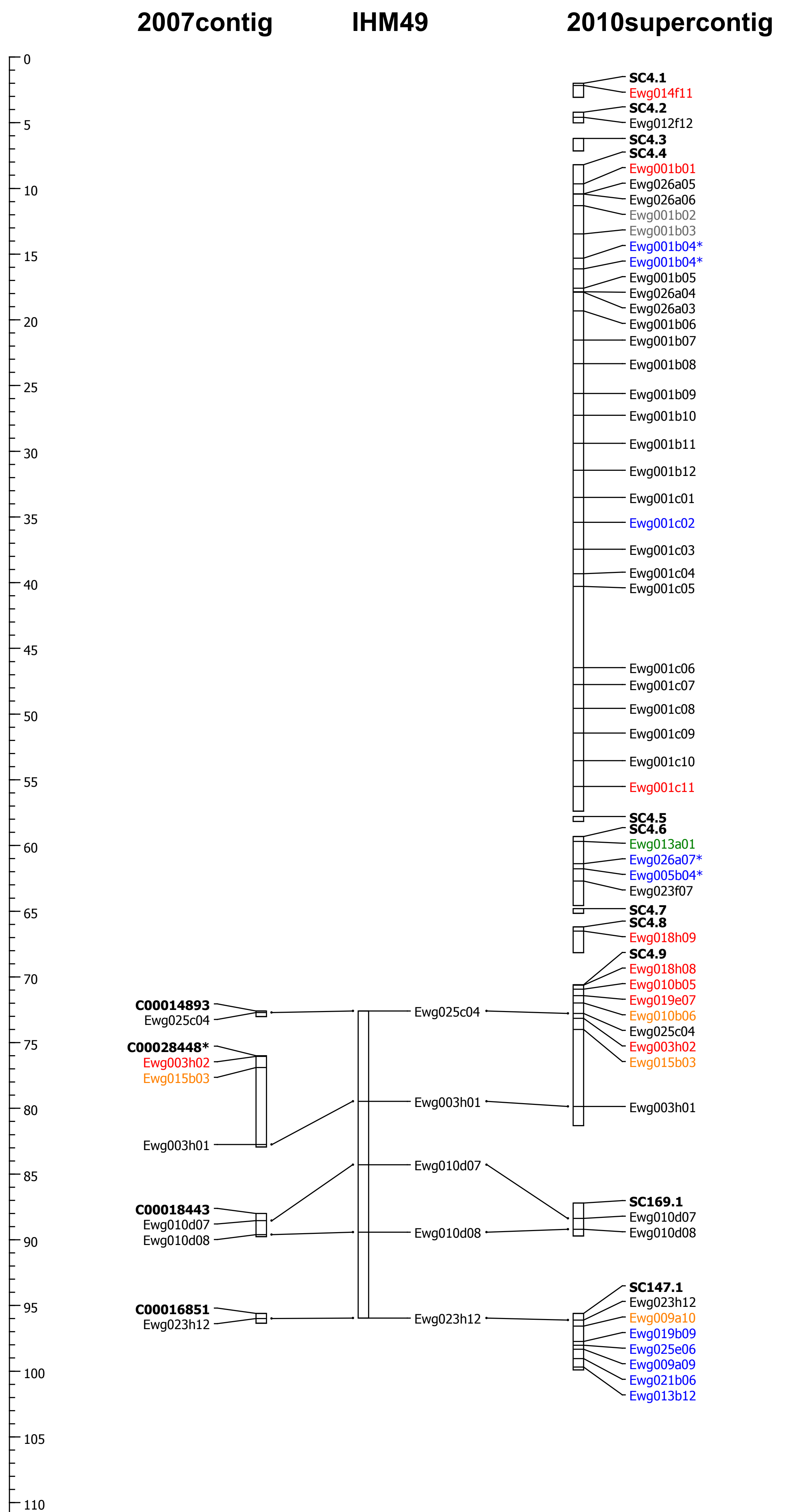
2007contig
IHM49
2010supercontig
0
5
10
15
20
25
30
35
40
45
50
55
60
65
70
75
80
85
90
95
100
105
110
SC4.1
Ewg014f11
SC4.2
Ewg012f12
SC4.3
SC4.4
Ewg001b01
Ewg026a05
Ewg026a06
Ewg001b02
Ewg001b03
Ewg001b04*
Ewg001b04*
Ewg001b05
Ewg026a04
Ewg026a03
Ewg001b06
Ewg001b07
Ewg001b08
Ewg001b09
Ewg001b10
Ewg001b11
Ewg001b12
Ewg001c01
Ewg001c02
Ewg001c03
Ewg001c04
Ewg001c05
Ewg001c06
Ewg001c07
Ewg001c08
Ewg001c09
Ewg001c10
Ewg001c11
SC4.5
SC4.6
Ewg013a01
Ewg026a07*
Ewg005b04*
Ewg023f07
SC4.7
SC4.8
Ewg018h09
SC4.9
Ewg018h08
Ewg010b05
Ewg019e07
Ewg010b06
Ewg025c04
Ewg003h02
Ewg015b03
Ewg003h01
SC169.1
Ewg010d07
Ewg010d08
SC147.1
Ewg023h12
Ewg009a10
Ewg019b09
Ewg025e06
Ewg009a09
Ewg021b06
Ewg013b12
C00014893
Ewg025c04
C00028448*
Ewg003h02
Ewg015b03
Ewg003h01
C00018443
Ewg010d07
Ewg010d08
C00016851
Ewg023h12
Ewg025c04
Ewg003h01
Ewg010d07
Ewg010d08
Ewg023h12

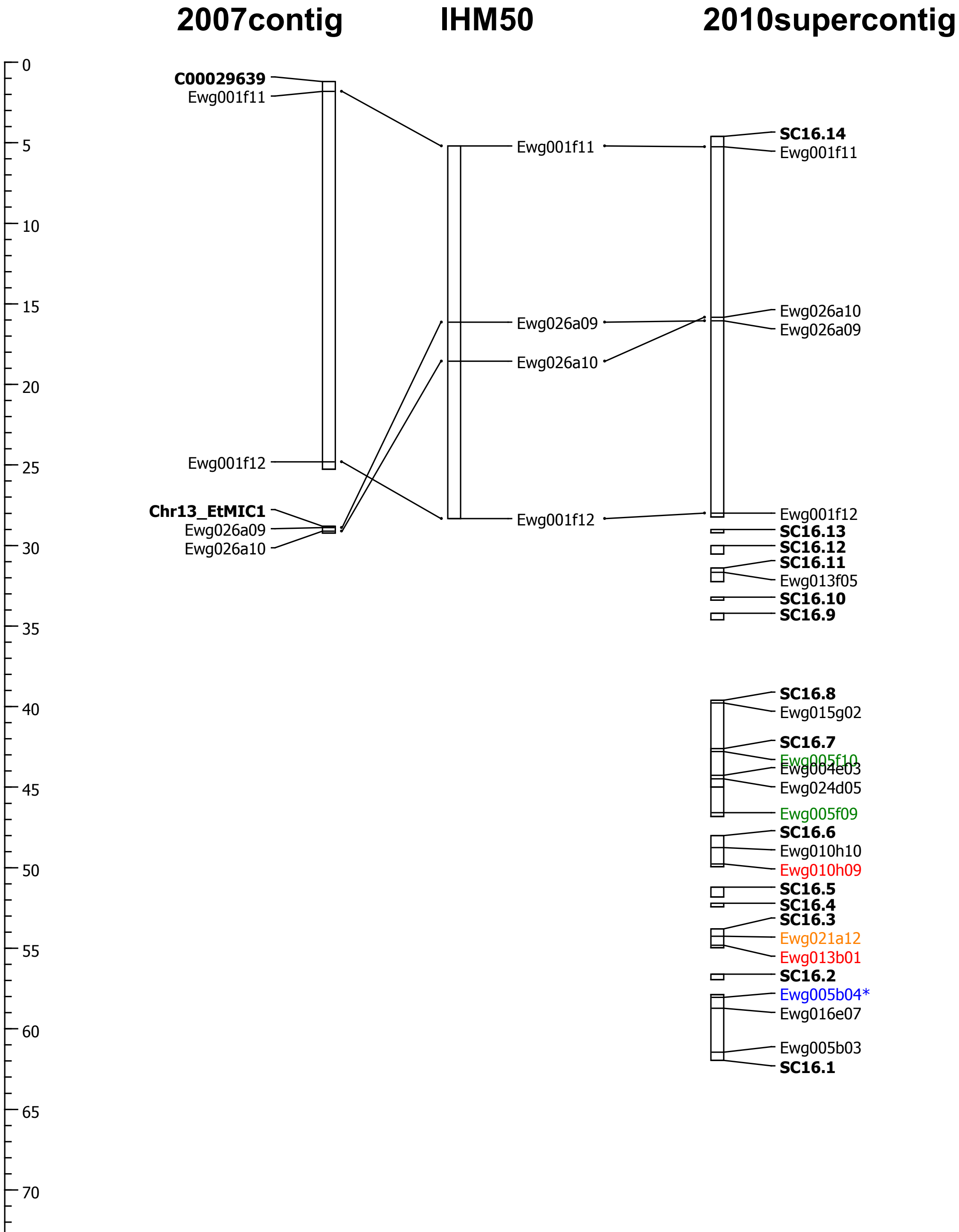
2007contig
IHM50
2010supercontig
0
5
10
15
20
25
30
35
40
45
50
55
60
65
70
C00029639
Ewg001f11
Ewg001f12
Chr13_EtMIC1
Ewg026a09
Ewg026a10
Ewg001f11
Ewg026a09
Ewg026a10
Ewg001f12
SC16.14
Ewg001f11
Ewg026a10
Ewg026a09
Ewg001f12
SC16.13
SC16.12
SC16.11
Ewg013f05
SC16.10
SC16.9
SC16.8
Ewg015g02
SC16.7
Ewg005f10
Ewg004e03
Ewg024d05
Ewg005f09
SC16.6
Ewg010h10
Ewg010h09
SC16.5
SC16.4
SC16.3
Ewg021a12
Ewg013b01
SC16.2
Ewg005b04*
Ewg016e07
Ewg005b03
SC16.1

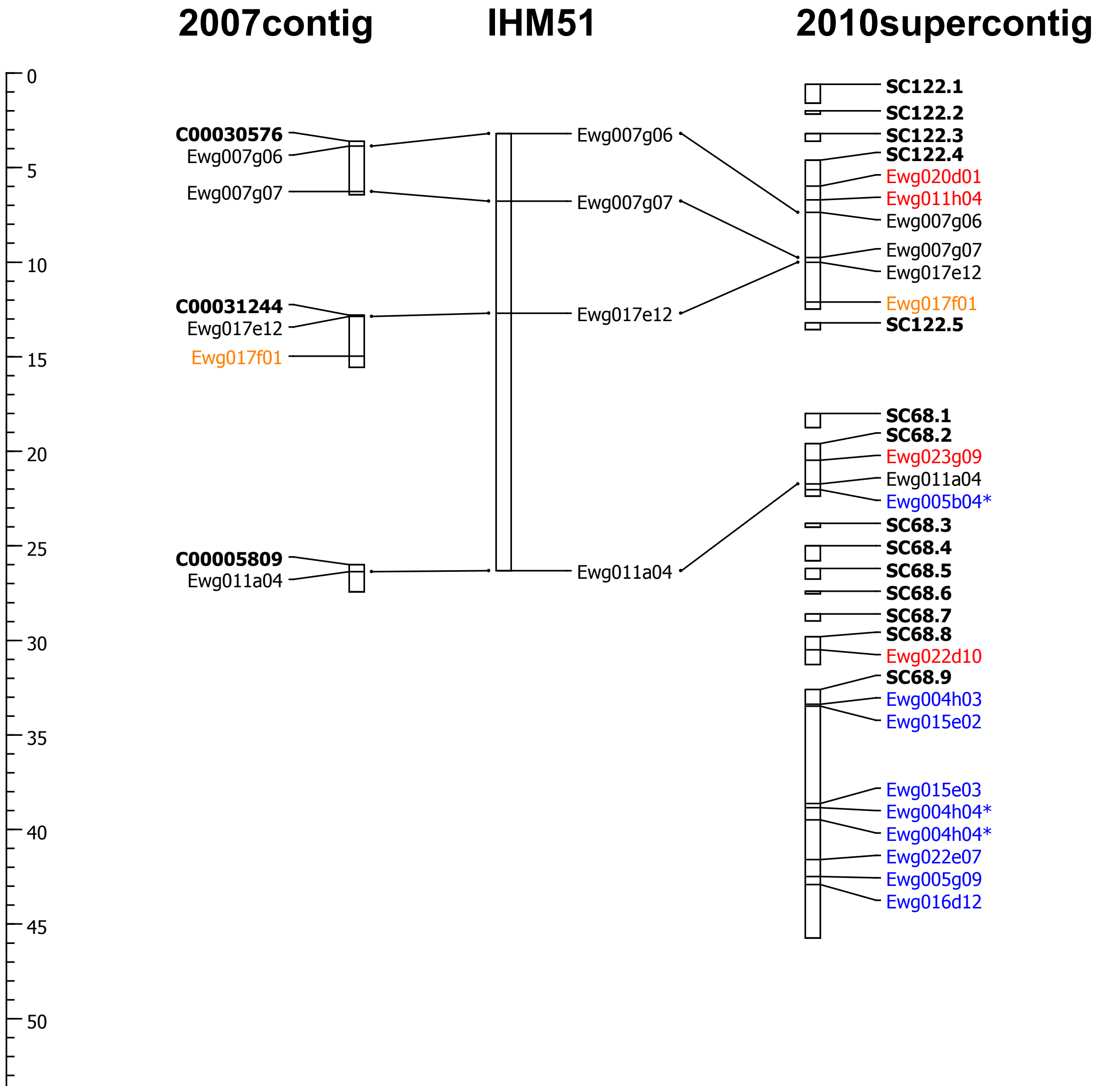

2007contig
IHM51
2010supercontig
0
5
10
15
20
25
30
35
40
45
50
C00030576
Ewg007g06
Ewg007g07
C00031244
Ewg017e12
Ewg017f01
C00005809
Ewg011a04
Ewg007g06
Ewg007g07
Ewg017e12
Ewg011a04
SC122.1
SC122.2
SC122.3
SC122.4
Ewg020d01
Ewg011h04
Ewg007g06
Ewg007g07
Ewg017e12
Ewg017f01
SC122.5
SC68.1
SC68.2
Ewg023g09
Ewg011a04
Ewg005b04*
SC68.3
SC68.4
SC68.5
SC68.6
SC68.7
SC68.8
Ewg022d10
SC68.9
Ewg004h03
Ewg015e02
Ewg015e03
Ewg004h04*
Ewg004h04*
Ewg022e07
Ewg005g09
Ewg016d12

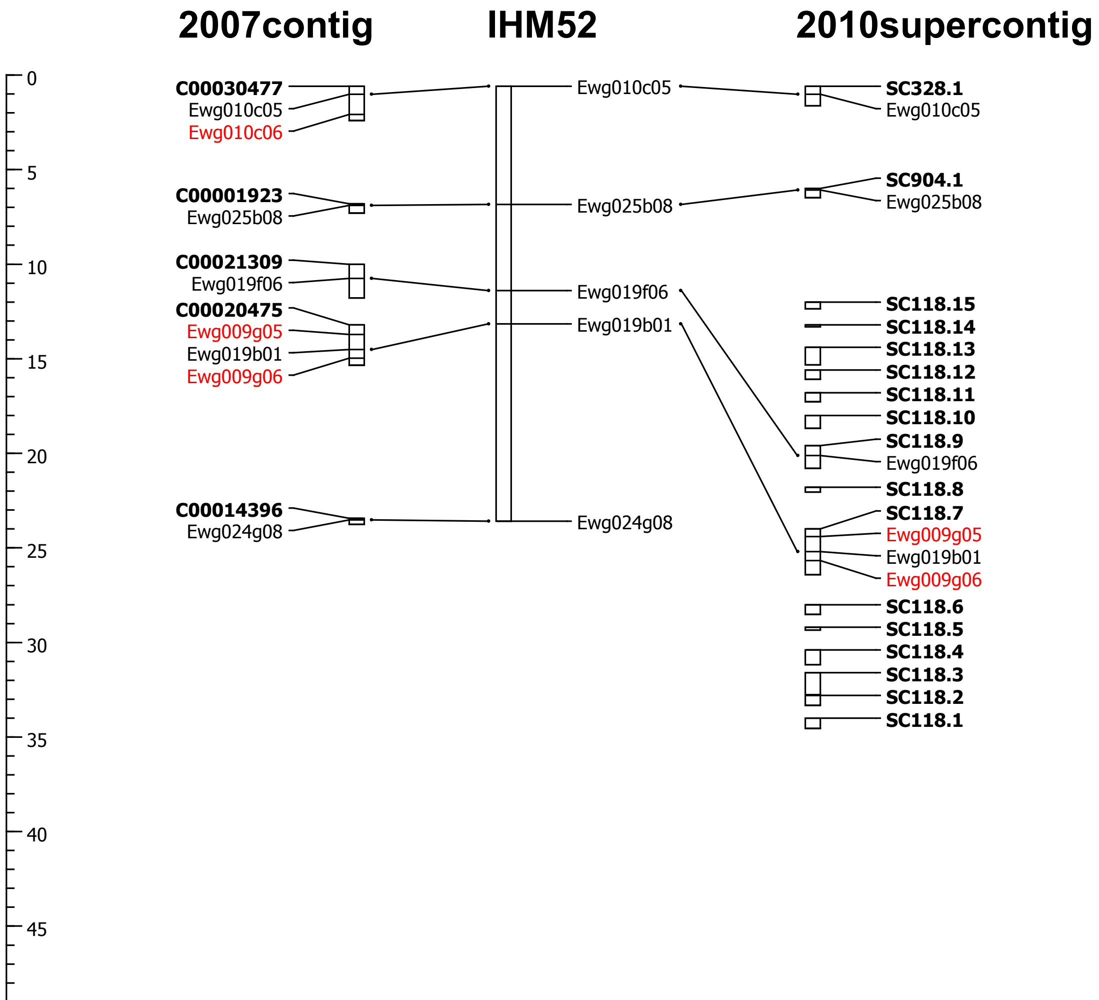

2007contig
IHM52
2010supercontig
0
5
10
15
20
25
30
35
40
45
C00030477
Ewg010c05
Ewg010c06
C00001923
Ewg025b08
C00021309
Ewg019f06
C00020475
Ewg009g05
Ewg019b01
Ewg009g06
C00014396
Ewg024g08
Ewg010c05
Ewg025b08
Ewg019f06
Ewg019b01
Ewg024g08
SC328.1
Ewg010c05
SC904.1
Ewg025b08
SC118.15
SC118.14
SC118.13
SC118.12
SC118.11
SC118.10
SC118.9
Ewg019f06
SC118.8
SC118.7
Ewg009g05
Ewg019b01
Ewg009g06
SC118.6
SC118.5
SC118.4
SC118.3
SC118.2
SC118.1

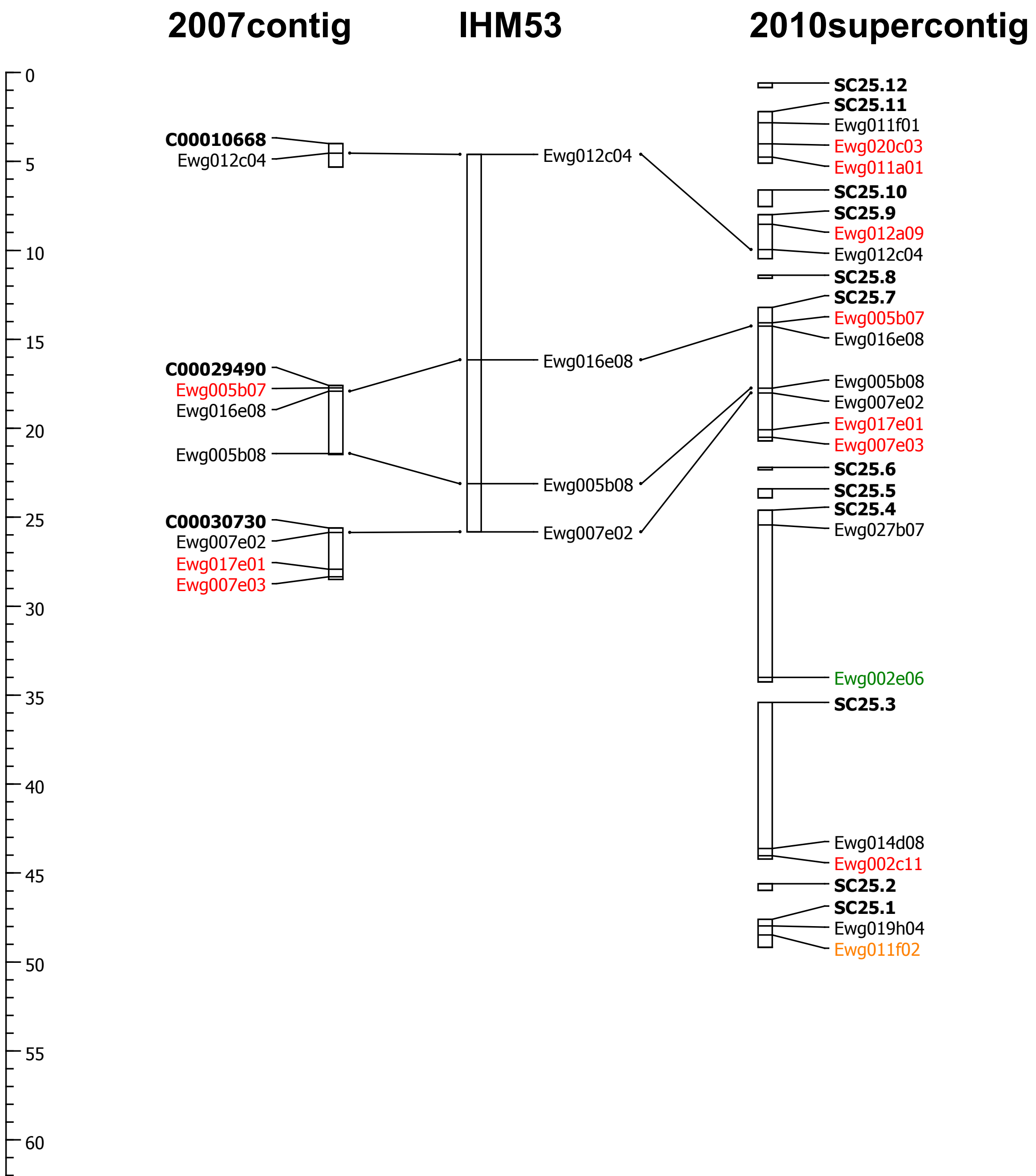

2007contig
IHM53
2010supercontig
0
5
10
15
20
25
30
35
40
45
50
55
60
C00010668
Ewg012c04
C00029490
Ewg005b07
Ewg016e08
Ewg005b08
C00030730
Ewg007e02
Ewg017e01
Ewg007e03
Ewg012c04
Ewg016e08
Ewg005b08
Ewg007e02
SC25.12
SC25.11
Ewg011f01
Ewg020c03
Ewg011a01
SC25.10
SC25.9
Ewg012a09
Ewg012c04
SC25.8
SC25.7
Ewg005b07
Ewg016e08
Ewg005b08
Ewg007e02
Ewg017e01
Ewg007e03
SC25.6
SC25.5
SC25.4
Ewg027b07
Ewg002e06
SC25.3
Ewg014d08
Ewg002c11
SC25.2
SC25.1
Ewg019h04
Ewg011f02

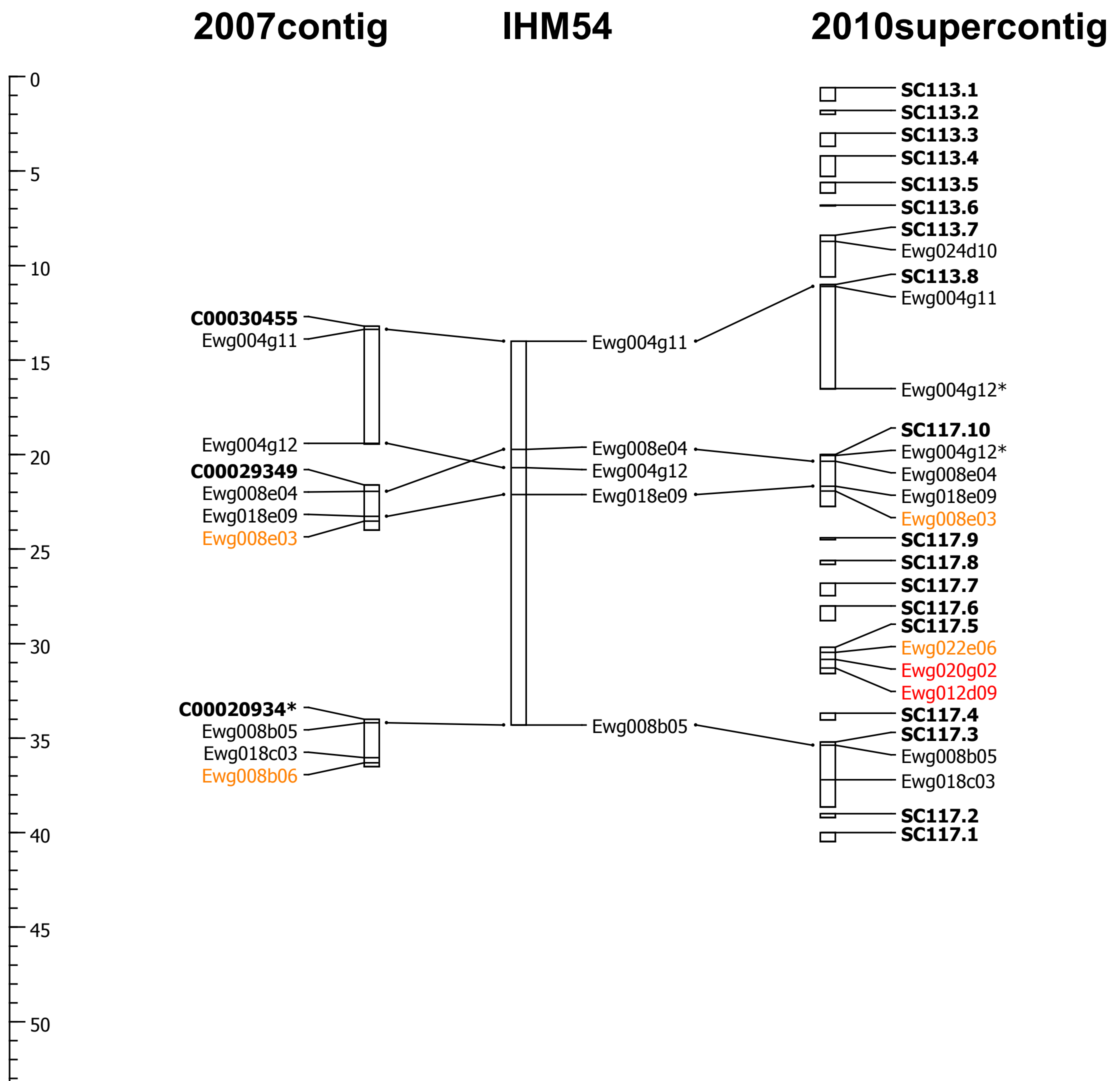
2007contig
IHM54
2010supercontig
0
5
10
15
20
25
30
35
40
45
50
C00030455
Ewg004g11
Ewg004g12
C00029349
Ewg008e04
Ewg018e09
Ewg008e03
C00020934*
Ewg008b05
Ewg018c03
Ewg008b06
Ewg004g11
Ewg008e04
Ewg004g12
Ewg018e09
Ewg008b05
SC113.1
SC113.2
SC113.3
SC113.4
SC113.5
SC113.6
SC113.7
Ewg024d10
SC113.8
Ewg004g11
Ewg004g12*
SC117.10
Ewg004g12*
Ewg008e04
Ewg018e09
Ewg008e03
SC117.9
SC117.8
SC117.7
SC117.6
SC117.5
Ewg022e06
Ewg020g02
Ewg012d09
SC117.4
SC117.3
Ewg008b05
Ewg018c03
SC117.2
SC117.1

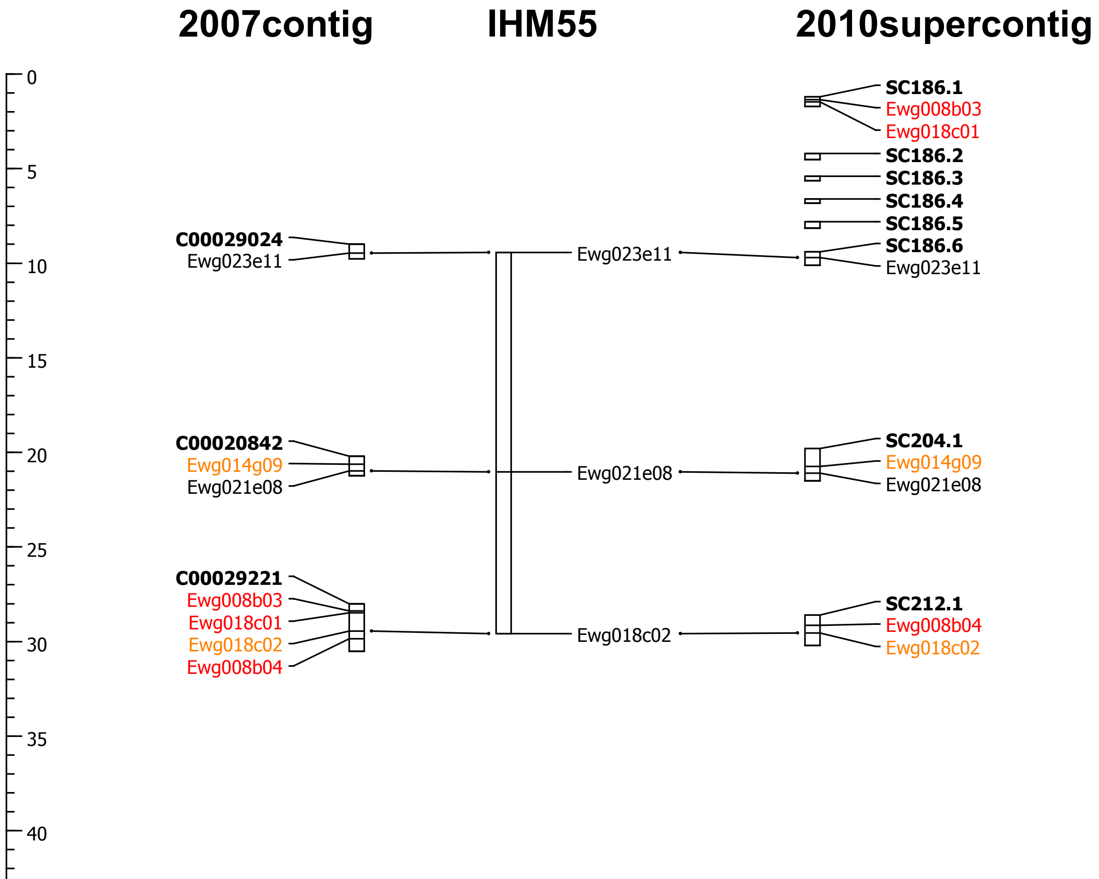
2007contig
IHM55
2010supercontig
0
5
10
15
20
25
30
35
40
C00029024
Ewg023e11
C00020842
Ewg014g09
Ewg021e08
C00029221
Ewg008b03
Ewg018c01
Ewg018c02
Ewg008b04
Ewg023e11
Ewg021e08
Ewg018c02
SC186.1
Ewg008b03
Ewg018c01
SC186.2
SC186.3
SC186.4
SC186.5
SC186.6
Ewg023e11
SC204.1
Ewg014g09
Ewg021e08
SC212.1
Ewg008b04
Ewg018c02

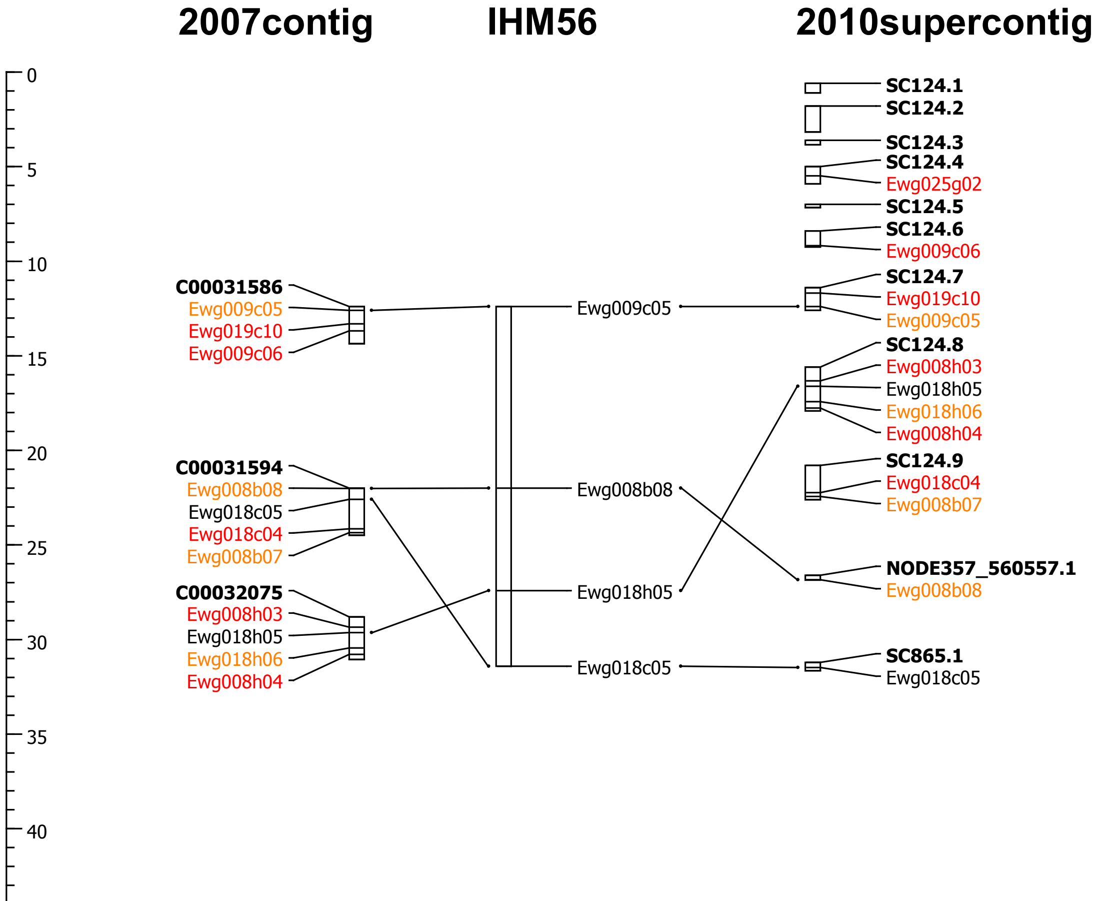
2007contig
IHM56
2010supercontig
0
5
10
15
20
25
30
35
40
C00031586
Ewg009c05
Ewg019c10
Ewg009c06
C00031594
Ewg008b08
Ewg018c05
Ewg018c04
Ewg008b07
C00032075
Ewg008h03
Ewg018h05
Ewg018h06
Ewg008h04
Ewg009c05
Ewg008b08
Ewg018h05
Ewg018c05
SC124.1
SC124.2
SC124.3
SC124.4
Ewg025g02
SC124.5
SC124.6
Ewg009c06
SC124.7
Ewg019c10
Ewg009c05
SC124.8
Ewg008h03
Ewg018h05
Ewg018h06
Ewg008h04
SC124.9
Ewg018c04
Ewg008b07
NODE357_560557.1
Ewg008b08
SC865.1
Ewg018c05

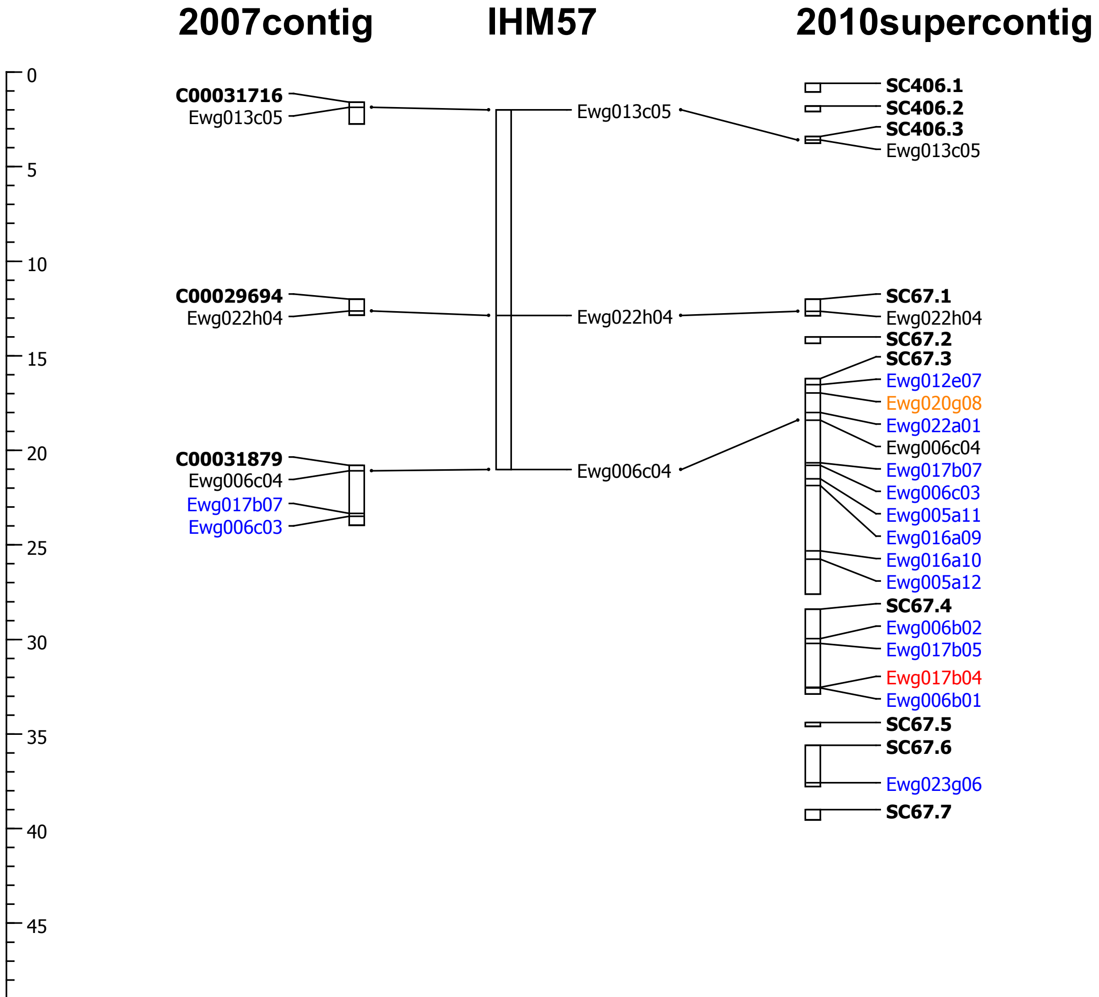

2007contig
IHM57
2010supercontig
0
5
10
15
20
25
30
35
40
45
C00031716
Ewg013c05
C00029694
Ewg022h04
C00031879
Ewg006c04
Ewg017b07
Ewg006c03
Ewg013c05
Ewg022h04
Ewg006c04
SC406.1
SC406.2
SC406.3
Ewg013c05
SC67.1
Ewg022h04
SC67.2
SC67.3
Ewg012e07
Ewg020g08
Ewg022a01
Ewg006c04
Ewg017b07
Ewg006c03
Ewg005a11
Ewg016a09
Ewg016a10
Ewg005a12
SC67.4
Ewg006b02
Ewg017b05
Ewg017b04
Ewg006b01
SC67.5
SC67.6
Ewg023g06
SC67.7

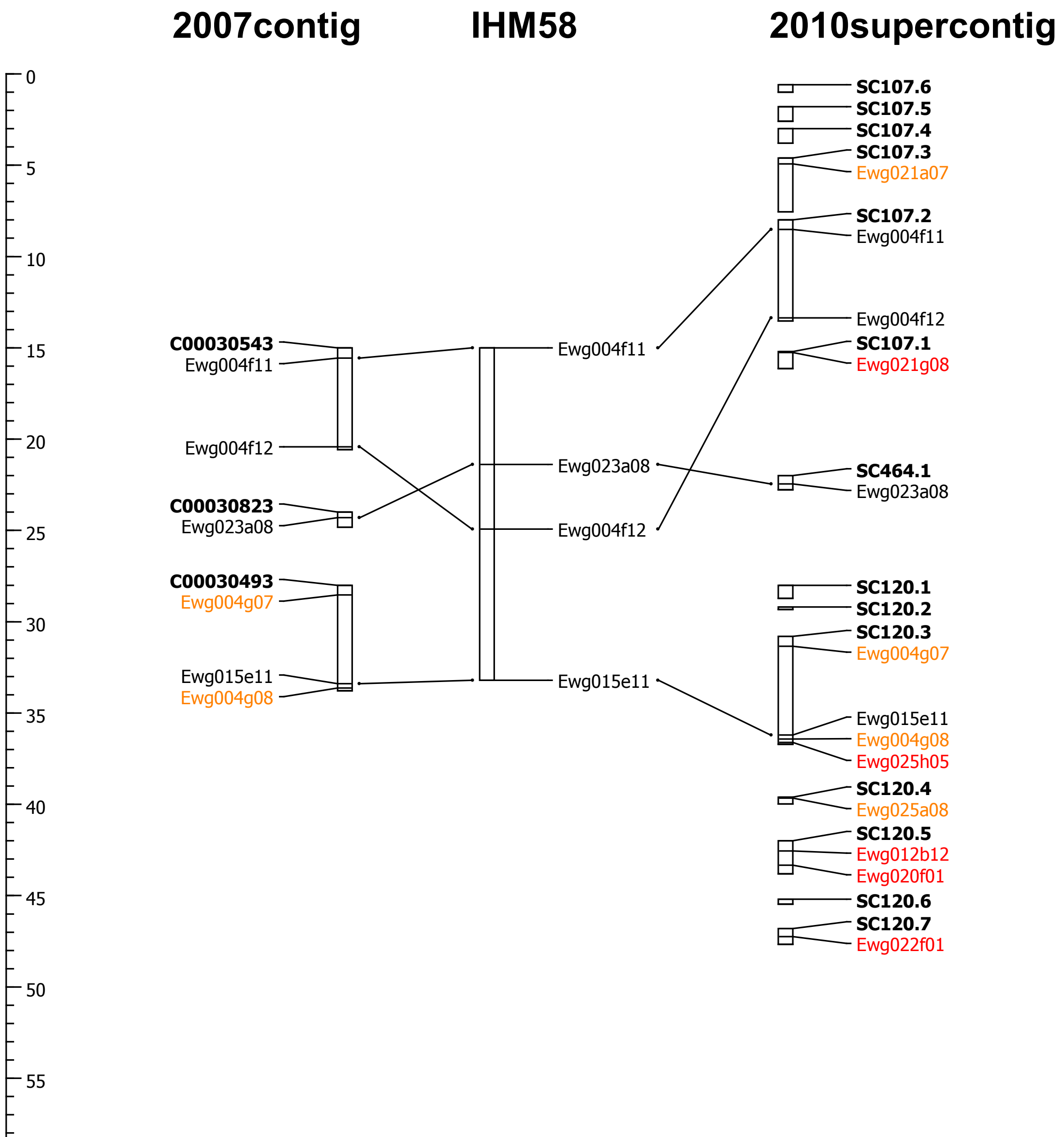

2007contig
IHM58
2010supercontig
0
5
10
15
20
25
30
35
40
45
50
55
C00030543
Ewg004f11
Ewg004f12
C00030823
Ewg023a08
C00030493
Ewg004g07
Ewg015e11
Ewg004g08
Ewg004f11
Ewg023a08
Ewg004f12
Ewg015e11
SC107.6
SC107.5
SC107.4
SC107.3
Ewg021a07
SC107.2
Ewg004f11
Ewg004f12
SC107.1
Ewg021g08
SC464.1
Ewg023a08
SC120.1
SC120.2
SC120.3
Ewg004g07
Ewg015e11
Ewg004g08
Ewg025h05
SC120.4
Ewg025a08
SC120.5
Ewg012b12
Ewg020f01
SC120.6
SC120.7
Ewg022f01

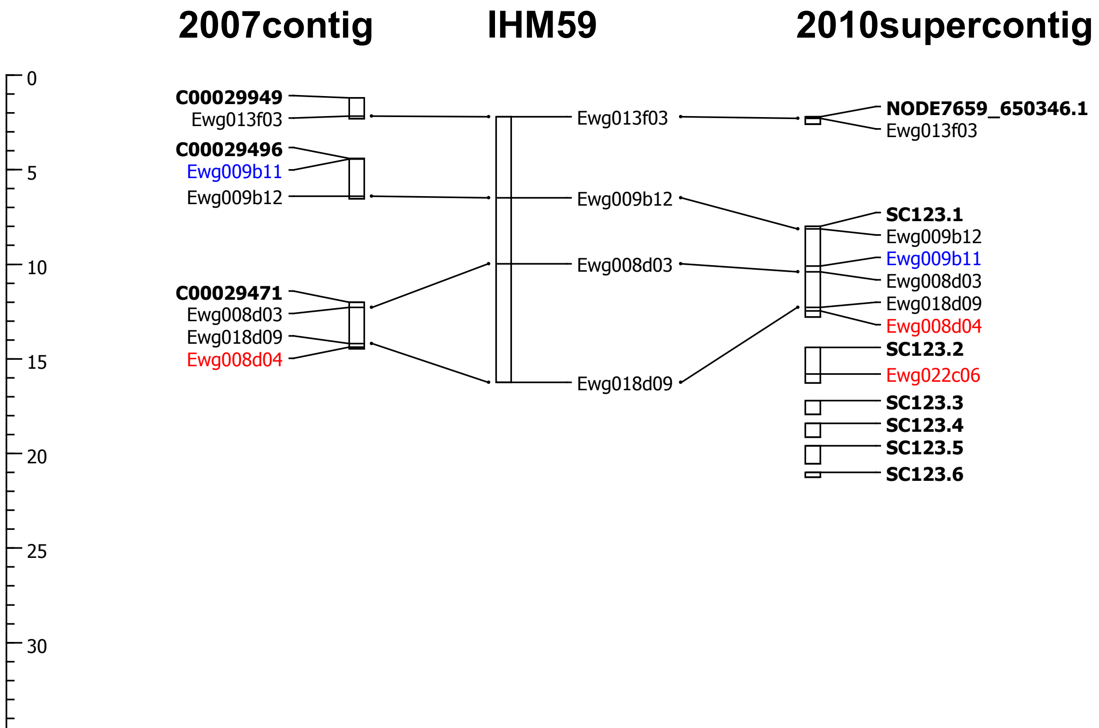
2007contig
IHM59
2010supercontig
0
5
10
15
20
25
30
C00029949
Ewg013f03
C00029496
Ewg009b11
Ewg009b12
C00029471
Ewg008d03
Ewg018d09
Ewg008d04
Ewg013f03
Ewg009b12
Ewg008d03
Ewg018d09
NODE7659_650346.1
Ewg013f03
SC123.1
Ewg009b12
Ewg009b11
Ewg008d03
Ewg018d09
Ewg008d04
SC123.2
Ewg022c06
SC123.3
SC123.4
SC123.5
SC123.6

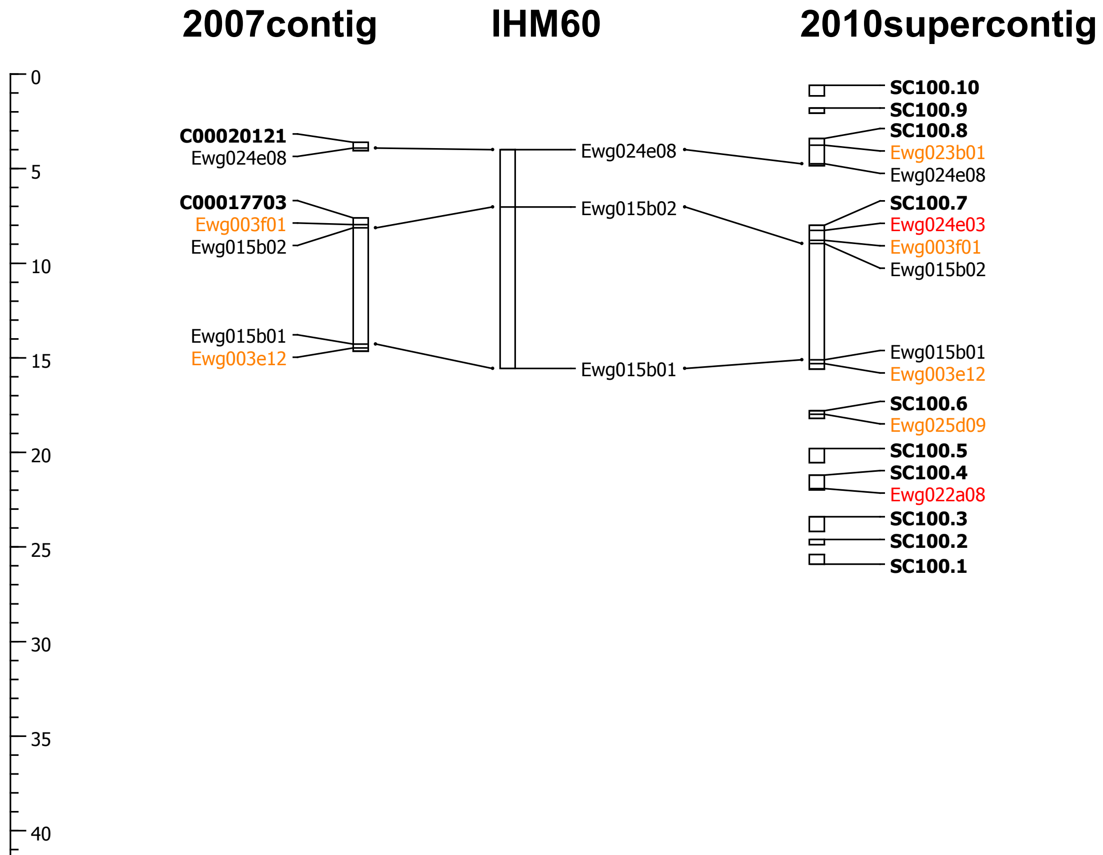

2007contig
IHM60
2010supercontig
0
5
10
15
20
25
30
35
40
C00020121
Ewg024e08
C00017703
Ewg003f01
Ewg015b02
Ewg015b01
Ewg003e12
Ewg024e08
Ewg015b02
Ewg015b01
SC100.10
SC100.9
SC100.8
Ewg023b01
Ewg024e08
SC100.7
Ewg024e03
Ewg003f01
Ewg015b02
Ewg015b01
Ewg003e12
SC100.6
Ewg025d09
SC100.5
SC100.4
Ewg022a08
SC100.3
SC100.2
SC100.1
